# Multivalent Rhamnose‐Modified EGFR‐Targeting Nanobody Gains Enhanced Innate Fc Effector Immunity and Overcomes Cetuximab Resistance via Recruitment of Endogenous Antibodies

**DOI:** 10.1002/advs.202307613

**Published:** 2024-01-29

**Authors:** Yanchun Li, Han Lin, Haofei Hong, Dan Li, Liang Gong, Jie Zhao, Zheng Wang, Zhimeng Wu

**Affiliations:** ^1^ The Key Laboratory of Carbohydrate Chemistry & Biotechnology Ministry of Education School of Biotechnology Jiangnan University Wuxi 214122 China

**Keywords:** antibody recruiting, cetuximab resistance, Fc effector functions, multivalent rhamnose, nanobody

## Abstract

Cetuximab resistance is a significant challenge in cancer treatment, requiring the development of novel therapeutic strategies. In this study, a series of multivalent rhamnose (Rha)‐modified nanobody conjugates are synthesized and their antitumor activities and their potential to overcome cetuximab resistance are investigated. Structure‐activity relationship studies reveal that the multivalent conjugate **D5**, bearing sixteen Rha haptens, elicits the most potent innate fragment crystallizable (Fc) effector immunity in vitro and exhibits an excellent in vivo pharmacokinetics by recruiting endogenous antibodies. Notably, it is found that the optimal conjugate **D5** represents a novel entity capable of reversing cetuximab‐resistance induced by serine protease (PRSS). Moreover, in a xenograft mouse model, conjugate **D5** exhibits significantly improved antitumor efficacy compared to unmodified nanobodies and cetuximab. The findings suggest that Rha‐Nanobody (Nb) conjugates hold promise as a novel therapeutic strategy for the treatment of cetuximab‐resistant tumors by enhancing the innate Fc effector immunity and enhancing the recruitment of endogenous antibodies to promote cancer cell clearance by innate immune cells.

## Graphical abstract

Multivalent rhamnose(Rha)‐7D12 conjugates are developed to investigate the recruitment of anti‐Rha antibody capability and the induction of Fc‐mediated immunity against EGFR‐positive cancer cells. The multivalent conjugates are completely resistant to serine protease PRSS degradation secreted by cetuximab‐resistant tumor cells and trigger a stronger Fc‐mediated immunity through the engagement of endogenous anti‐Rha antibodies both in vitro and in vivo.

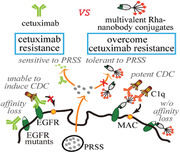

## 1 Introduction

The epidermal growth factor receptor (EGFR) is a member of the ErbB family or receptor tyrosine kinases that includes ErbB2 (HER2), ErbB3 (HER3), and ErbB4 (HER4). EGFR is a transmembrane glycoprotein that plays a crucial role in regulating cell differentiation and proliferation.^[^ ^1^ ^]^ EGFR overexpression is aberrantly activated in various cancers, including lung, breast, and colorectal cancers. Upon the binding of specific ligands such as epidermal growth factor (EGF), transforming growth factor α (TGF‐α), amphiregulin, and epigen, EGFR undergoes homologous or heterogenous dimerization, and phosphorylation of the tyrosine residue in the intracellular domain. The phosphorylated EGFR activates downstream signaling pathways, such as the Ras/Raf/MAPK, PI3K/AKT, PLC/PKC, and STAT pathways, that promote tumor cell growth and progression.^[^ ^2^ ^]^ As a result, EGFR has emerged as a clinically validated target for cancer treatment and diagnosis, with various anti‐EGFR monoclonal antibodies (mAbs), such as cetuximab, panitumumab, nimotuzumab, and necitumumab having obtained FDA regulatory approval for multiple cancer treatments.^[^ ^3^ ^]^ Mechanistic studies have demonstrated that anti‐EGFR mAbs exert their therapeutic effects by blocking ligand binding, inhibiting receptor dimerization, and promoting receptor internalization and degradation.^[^ ^4^ ^]^ Furthermore, anti‐EGFR mAbs can trigger potent innate fragment crystallizable (Fc) effector functions through antibody‐mediated immune response mechanisms such as antibody‐dependent cell‐mediated cytotoxicity (ADCC), complement‐mediated cytotoxicity (CDC), and antibody‐dependent cellular phagocytosis (ADCP), to promote efficient cancer cell death.^[^ ^5^ ^]^

However, the clinical therapeutic efficacy of anti‐EGFR mAb, including cetuximab, is significantly limited by the emergence of acquired resistance, which is commonly observed in clinical settings.^[^ ^6^ ^]^ One of the critical underlying molecular mechanisms responsible for cetuximab resistance is a mutation in the extracellular domain (ECD) of EGFR.^[^ ^7^ ^]^ This mutation can abolish the binding of anti‐EGFR mAbs and result in the development of acquired resistance in patients. Approximately 16% of patients with metastatic colorectal cancer (mCRC), have developed the EGFR S492R mutation following treatment with cetuximab.^[^ ^8^ ^]^ Mutations of various other residues of EGFR (e.g., V441, I462, S464, G465, K467, K489, I491) have also been associated with the ability of the receptor to confer clinical resistance to cetuximab.^[^ ^7^, ^9^ ^]^ Efforts to circumvent the cetuximab resistance resulting from EGFR ECD mutations have been primarily focused on developing new anti‐EGFR mAbs or utilizing combinatorial strategies. Examples of such approaches include necitumumab (targeting EGFR S468R),^[^ ^10^ ^]^ newly discovered cetuximab variants to reverse EGFRS492R or EGFRG465R‐driven resistance,^[^ ^11^ ^]^ Sym004 (a 1:1 mixture of mAbs against distinct EGFR epitopes),^[^ ^12^ ^]^ and MM‐151 (targeting multiple regions of the EGFR ECD).^[^ ^13^ ^]^ Additionally, enhancing antibody Fc effector functions has been shown to be an effective approach for overcoming cetuximab resistance, even when the binding affinity is impaired due to EGFR ECD mutations.^[^ ^14^ ^]^ Most recently, a new cetuximab resistance mechanism in colorectal cancer, which is mediated by a serine protease PRSS, has been identified. This mechanism involves the cleavage of cetuximab by tumor cells secreted PRSS, ultimately leading to reduced therapeutic efficacy.^[^ ^15^ ^]^ Detailed investigation has demonstrated that aberrant PRSS expression was closely associated with poor cetuximab therapy in mCRC patients and led to lower progression‐free survival. Howerver, circumventing cetuximab resistance caused by PRSS requires the design of new cetuximab variants. Despite the success of various anti‐EGFR mAb‐based strategies for overcoming cetuximab resistance in EGFR‐positive cancer treatment, the development of a novel strategy that incorporate multiple molecular mechanisms to overcome cetuximab resistance may improve therapeutic efficacy and index in cancer treatment.

Nanobodies are a unique type of heavy‐chain‐only antibody that are derived from camelids and exhibit improved physiochemical properties, thermal stability, solubility, and proteolytic resistance compared to conventional antibodies.^[^ ^16^ ^]^ As the smallest antigen‐binding fragment, the long protruding complementarity‐determining region 3 (CDR3) loop in nanobodies allows them to access small or hidden epitopes that are difficult to target by full‐length mAbs. However, the lack of an Fc fragment and poor pharmacokinetics are two key limitations that hinder the antitumor efficacy and clinical translation of nanobody‐based therapeutics.^[^ ^17^ ^]^ Human serum naturally contains high levels of hapten‐specific endogenous antibodies against 2,4‐dinitrophenyl (DNP),^[^ ^18^ ^]^ galactose‐α‐(1,3)‐galactose (αGal),^[^ ^19^ ^]^ and rhamnose (Rha).^[^ ^20^ ^]^ Recently, a novel strategy utilizing nanobody‐hapten conjugates, which could simultaneously restore the deficient Fc effector functions and improve the poor pharmacokinetics of nanobodies, was developed.^[^ ^21^ ^]^ This strategy has the ability to redirect hapten‐specific endogenous antibodies to the surface of cancer cells, thereby promoting cell lysis through Fc‐mediated immune responses such as ADCC, CDC, and ADCP. The half‐lives of nanobody‐hapten conjugates are significantly prolonged through the formation of immunocomplexes with endogenous antibodies. However, monovalent nanobody‐hapten conjugates have been found to have lower antitumor efficacy. This is primarily attributed to the limited capability of the conjugates to recruit anti‐hapten antibodies and the low affinity of monovalent hapten for their corresponding antibodies.^[^ ^22^ ^]^ To address these limitations, the multivalent effect, which are pervasive throughout nature,^[^ ^23^ ^]^ was utilized to design multivalent antibody‐recruiting molecules containing multivalent haptens. These multivalent antibody‐recruiting molecules were able to promote endogenous antibody recruiting and enhance the cancer cell killing activity mediated by immune cells.^[^ ^24^ ^]^

**Figure 1:**
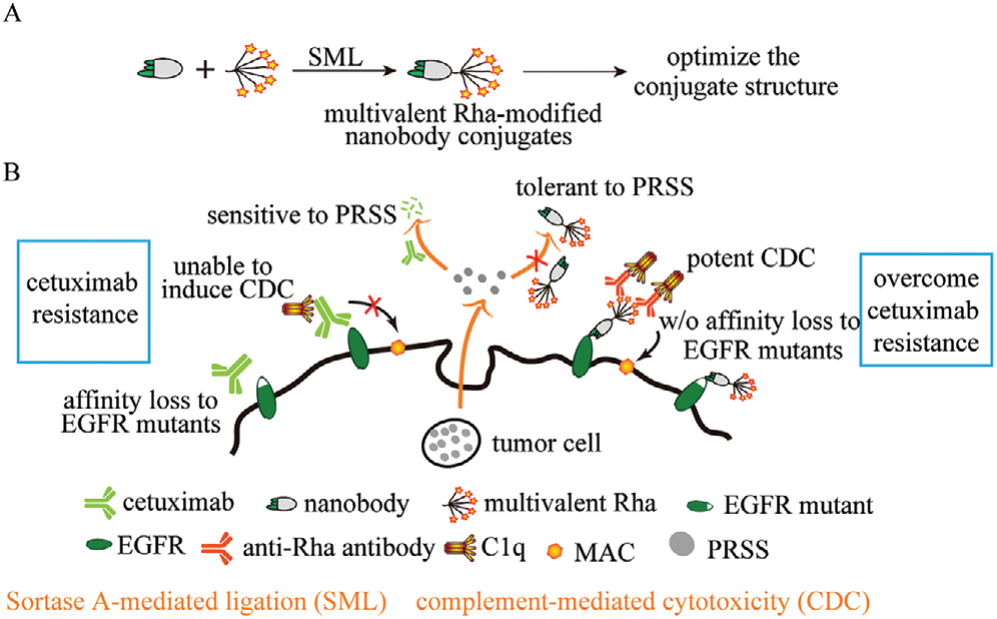
A) Design and construction of multivalent Rha‐modified nanobody conjugates to enhance immune response and B) overcome the cetuximab resistance in cancer immunotherapy.

In this study, a series of multivalent Rha haptens, ranging from monomeric‐ to hexadecameric, were designed and site‐specifically conjugated to the EGFR‐targeting nanobody 7D12 using sortase A‐mediated ligation (SML). The resulting multivalent Rha‐7D12 conjugates were used to investigate the structure‐activity relationship in terms of recruiting endogenous anti‐Rha antibody capability and eliciting Fc effector functions against EGFR‐positive cancer cells (**Figure** **1**A). Importantly, we found that the multivalent Rha‐7D12 conjugates were completely resistant to degradation by PRSS secreted by cetuximab‐resistant tumor cells (Figure 1B). The pharmacokinetics of the optimized multivalent conjugate and its antitumor efficacy in cetuximab‐resistant xenograft mice, were evaluated in vivo. Given that the anti‐EGFR nanobody 7D12 can bind and block EGFR with all known acquired resistance mutations,^[^ ^25^ ^]^ our findings suggest that the multivalent Rha‐7D12 conjugate, with its enhanced Fc effector functions and proteolytic resistance, represents a promising modality for overcoming cetuximab resistance in cancer immunotherapy.

## 2 Results and Discussion

**Scheme 1:**
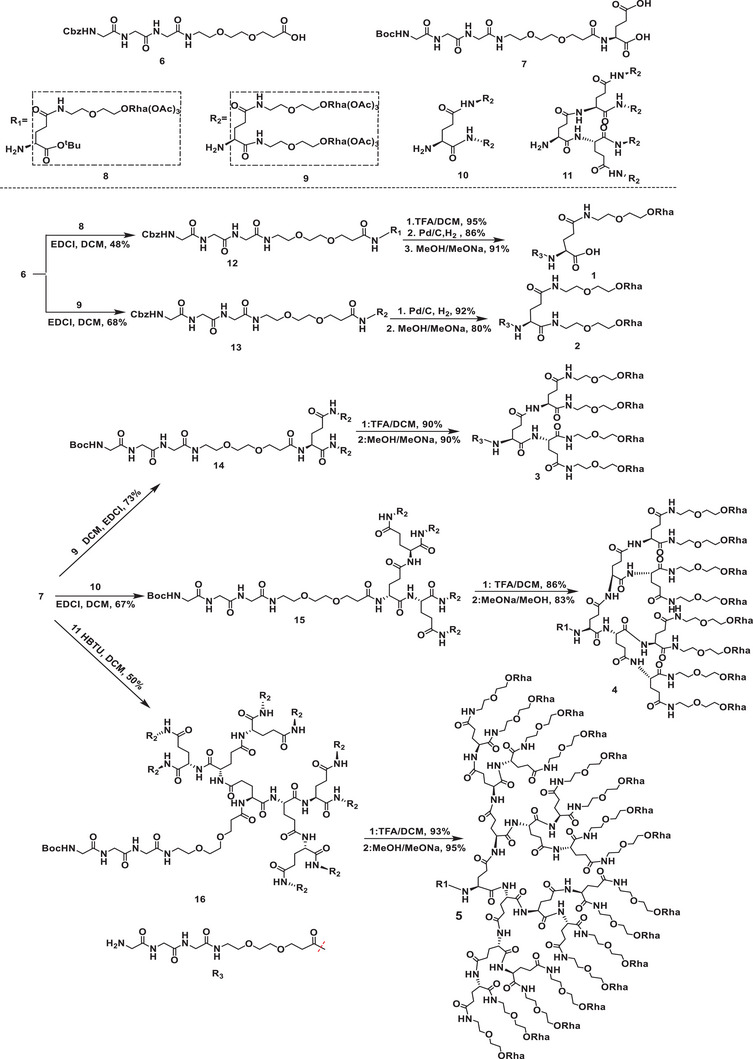
Synthesis of oligoglycine‐containing multivalent Rha haptens **1**–**5**.

SML is a powerful tool for the site‐specific modification of proteins and peptides.^[^ ^21a,c^ ^]^ To conjugate the multivalent Rha haptens to nanobodies using SML, we designed and synthesized a series of oligoglycine‐containing Rha derivatives, which included monomer **1**, dimer **2**, tetramer **3**, octamer **4**, and hexadecamer **5**. The synthesis of these compounds was carried out according to the procedure outlined in **Scheme** **1**. The key intermediates **6**–**11** were synthesized through multiple steps as described in Supplementary Information. The multivalent Rha haptens were then assembled from these intermediates. Compound **6** was coupled with either **8** or **9** using 1‐(3‐Dimethylaminopropyl)−3‐ethylcarbodiimide hydrochloride (EDCI·HCl) as a condensation reagent to afford **12** and **13** in moderate yields. The protecting groups were then removed to yield the monomeric‐ and dimeric haptens **1** and **2**. Likewise, compound **7** was coupled to **9**, **10**, or **11** to yield **14**, **15**, and **16**, respectively. Subsequently, the *tert*‐butyloxycarbonyl (Boc) and acetyl protecting groups were removed using 50% trifluoroacetic acid (TFA), and a Zemplén transesterification reaction to obtain tetramer **3**, octamer **4**, and hexadecamer **5**. All of the compounds were fully characterized by ^1^H and ^13^C‐NMR as well as mass spectrometry.

**Scheme 2:**
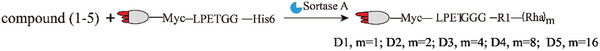
Chemoenzymatic synthesis of mono‐ and multivalent Rha‐modified nanobody 7D12 conjugates (**D1**‐**D5**).

The EGFR‐targeting nanobody 7D12, which contained a myc tag, an LPETG signal peptide, and a His tag at the C‐terminus, was recombinantly expressed in *E. coli* in a good yield. Site‐specific conjugation of multivalent Rha haptens **1**–**5** with nanobody 7D12 was achieved via SML as outlined in **Scheme** **2**. In brief, the reaction was performed by incubating the oligoglycine‐modified Rha haptens, nanobody 7D12, and eSrtA in a reaction buffer (50 mM Tris/HCl, 150 mM NaCl, 5 mM CaCl_2_, pH 7.5) at 16 °C for 2 h with constant shaking at 200 rpm. Nickel‐magnetic beads were then added to the reaction mixture to capture and remove the eSrtA and unreacted nanobody 7D12 bearing a 6xHis tag. Following further purification over a Sephadex G25 column or ultrafiltration, the Rha‐nanobody conjugates (**D1**–**D5**) were obtained in yields ranging from 70% to 83%. Sodium Dodecyl Sulphate‐Polyacrylamide Gel Electrophoresis (SDS‐PAGE) and Western blot analysis (detected using anti‐Rha and anti‐myc antibodies) demonstrated that conjugates **D1‐**‐**D5** were successfully formed (Figure S1, Supporting Information).

**Figure 2:**
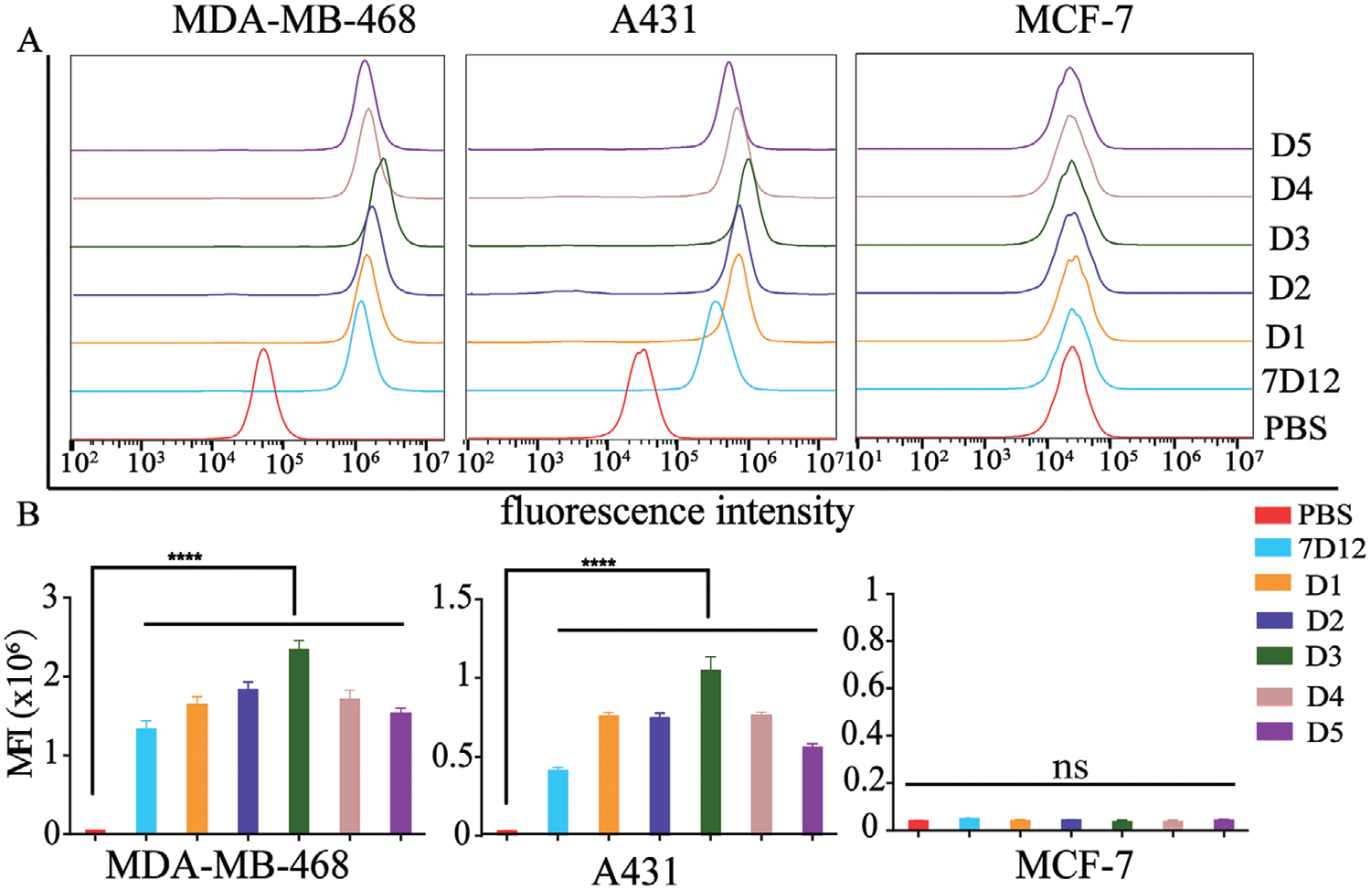
Binding specificity evaluation assays. A) Histograms and B) the corresponding MFI of cells treated with PBS, 7D12, conjugates (**D1**–**D5**). Data are reported as mean ± SD (*n* = 3) and the statistical significance is determined using Student's *t*‐test (two‐tailed). ^****^: *P*< 0.0001.

To assess the binding specificity of the Rha‐nanobody conjugates **D1**–**D5** to cancer cells, we employed EGFR‐positive human triple‐negative breast cancer MDA‐MB‐468 and human squamous carcinoma A431 cells, as well as EGFR‐negative human breast MCF‐7 cells as model cells (Figure S2, Supporting Information). All cells were incubated with phosphate buffered saline (PBS), 7D12, and Rha‐nanobody conjugates and subsequently treated with anti‐myc rabbit polyclonal antibodies and Dylight 488‐conjugated goat anti‐rabbit secondary antibodies. The cells were then analyzed using flow cytometry. Histograms (**Figure** **2**A) and the corresponding mean fluorescence intensities (MFIs) (Figure 2B) demonstrated that the EGFR‐positive MDA‐MB‐468 and A431 cancer cells treated with 7D12 or conjugates **D1**–**D5** displayed a stronger fluorescence signal than that of PBS‐treated cells. Conversely, no increases in the fluorescence intensity were observed in EGFR‐negative MCF‐7 cells treated with PBS, 7D12, or the conjugates. These results clearly demonstrated that the site‐specific rhamnosylation of nanobody 7D12 did not affect its ability to recognize target cells and that it maintained excellent binding specificity to EGFR‐positive cells.

**Figure 3:**
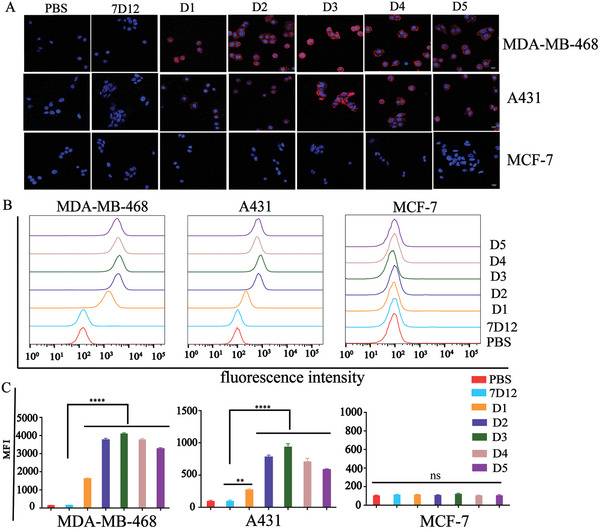
Anti‐Rha antibody recruiting assays. A) Confocal laser scanning microscopy images, B) histograms, and C) corresponding MFI of the cells treated with PBS, 7D12, monovalent conjugate **D1,** and multivalent conjugates (**D2**–**D5**). Red = Alexa Fluor 647 channel, blue = DAPI channel. Scale bar: 50 µm. Data are reported as mean ± SD (*n* = 3) and the statistical significance is determined using Student's t‐test (two‐tailed). ^**^: *P* < 0.01, ^****^: *P* < 0.0001.

We next investigated whether Rha‐nanobody conjugates **D1**–**D5** could recruit anti‐Rha antibodies onto the EGFR‐positive cell surface. Three cell lines—MDA‐MB‐468, A431, and MCF‐7—were treated with the conjugates and incubated with 1% rabbit serum containing a high level of anti‐Rha antibodies that were obtained by immunization with Rha‐ovalbumin (OVA) and Alexa Fluor 647‐conjugated goat anti‐rabbit IgG (H+L) secondary antibody. The cells were then imaged by confocal laser scanning microscopy (CLSM). Parallel experiments were performed using 7D12 or PBS as controls. As shown in **Figure** **3**A and Figures S3–S5 (Supporting Information), the surface of all EGFR‐positive cells (MDA‐MB‐468 and A431) were demonstrated bright red fluorescence signals when treated with **D1**–**D5** conjugates, indicating that the EGFR receptors on the surface of the cells were successfully labeled with the conjugates. However, no such labeling was observed in the 7D12‐treated cells due to the lack of Rha modification (Figures S3–S4, Supporting Information). As expected, no such labeling was observed on the surface of MCF‐7 cells because of the absence of EGFR expression (Figure S5, Supporting Information). These results demonstrated that the Rha‐7D12 conjugates were capable of recruiting and displaying anti‐Rha antibodies on the cells expressing EGFR.

To further quantitatively evaluate the ability of the Rha‐7D12 conjugates to recruit anti‐Rha antibodies, we conducted the above experiments and analysed the cells using flow cytometry. The histograms (Figure 3B) and MFIs results (Figure 3C) further confirmed that the EGFR‐positive cells (MDA‐MB‐468 and A431) exhibited a significant increase in MFI when incubated with conjugates **D1**–**D5**, while the 7D12 or PBS‐treated cells did not. The EGFR‐negative MCF‐7 cells did not exhibit any significant increase in MFI. The MFI measured from the A431 cells treated with **D1**–**D5** was 3.3, 9.6, 12.3, 8.8, and 7.1‐fold higher than that of the negative control 7D12 or PBS group, respectively. In the MDA‐MB‐468 cancer cells treated with conjugates **D1**–**D5,** the MFI increased 10, 22.5, 24.6, 22.6, and 20.1‐fold compared to that of the control group.

Notably, the MFI observed on the surface of EGFR‐positive cancer cells were associated with the structure of Rha‐nanobody conjugates, with the multivalent Rha‐7D12 conjugates (**D2**–**D5**) generally able to recruit more anti‐Rha antibodies than the monovalent conjugate **D1**. Similar results were observed in other EGFR‐positive cells, including A549 human non‐small cell lung cancer cells, SW480 human colorectal cancer cells, and HT‐29 cancer cells (Figure S6, Supporting Information). These results suggested that the introduction of multivalent Rha haptens into an antibody‐recruiting molecule can significantly improve its antibody‐recruiting capability. The MFI on the cell surface induced by the tetramer conjugate **D3** was slightly higher than those of the octamer and hexadecamer conjugates **D4** and **D5**. This phenomenon was likely attributed to the tetramer conjugate **D3** possessing an optimal stoichiometry for the interaction between the Rha haptens and anti‐Rha antibodies, thereby enabling the formation of highly stable immune complexes. These immune complexes might have had the ability to withstand multiple washing and centrifugation steps in the fluorescence‐activated cell sorting (FACS) assays, leading to the highest MFI signals amongst the multivalent Rha‐7D12 conjugates.^[^ ^26^ ^]^ Overall, the results of the CLSM and FACS assay demonstrated that multivalent Rha‐7D12 conjugates displayed a greater potential for recruiting anti‐Rha antibodies than monovalent conjugates, indicating that they may potentially elicit stronger Fc effector functions to kill cancer cells.

**Figure 4:**
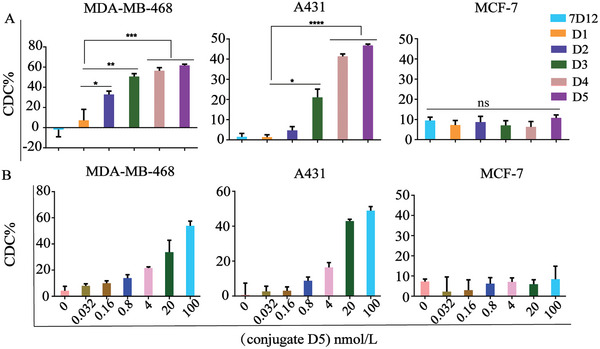
In vitro CDC assay. A) CDC mediated by 7D12, monovalent conjugate **D1**, multivalent conjugates (**D2**–**D5**); B) CDC mediated by different concentrations of conjugate **D5**. Data are reported as mean ± SD (*n* = 3) and the statistical significance is determined using Student's *t*‐test (two‐tailed). ^*^: *P*< 0.05, ^**^: *P*< 0.01, ^***^: *P*< 0.001, ^****^: *P* < 0.0001.

After demonstrating that the multivalent Rha‐7D12 conjugates had a high binding specificity for EGFR‐positive cancer cells and could effectively recruit anti‐Rha antibodies to the cancer cells, we sought to investigate whether these conjugates could induce Fc‐mediated immune responses in vitro, such as CDC and ADCP. To this end, we utilized EGFR‐positive MDA‐MB‐468 and A431cells, and EGFR‐negative MCF‐7 cells to assess CDC cytotoxicity. These cells were incubated with either 7D12, Rha‐7D12 conjugates (**D1–D5**) and cultured with 20% pooled human serum (HS) and 1% commercial human complement (HC) to provide anti‐Rha antibodies and a complement system for the CDC experiment to mimic in vivo conditions. The cell viability was evaluated using a commercial CCK8 kit. As shown in **Figure** **4**A, the percentage of the MDA‐MB‐468 cells treated with Rha‐7D12 conjugates **D1–D5** that underwent cell lysis was ≈7.3%, 32.8%, 50.7%, 56.5%, and 61.7%, respectively, while no obvious cell death was observed for the cells treated with 7D12. Similarly, the percentage of A431 cells treated with conjugates **D1–D5** that underwent cell lysis was 1.3%, 4.7%, 21.1%, 41.3%, and 46.7%, respectively. However, no significant cell lysis was observed in the EGFR‐negative MCF‐7 cells treatment with either 7D12 or the Rha‐7D12 conjugates. These results demonstrated that the CDC potency was closely associated with the Rha valence in conjugates as the multivalent Rha‐7D12 conjugates generally displayed more efficient cancer cell killing activity than monovalent conjugate. Furthermore, the extent of cell lysis mediated by CDC increased with the number of Rha haptens in conjugates, highlighting the importance of multivalency. Conjugate **D5** carrying sixteen Rha haptens displayed the most potent CDC activity among the conjugates, although it exhibited a slightly weaker antibody‐recruiting ability in vitro compared with the tetrameric conjugate **D3**. Moreover, a dose‐dependent CDC activity of conjugate **D5** was observed in EGFR‐positive cells (Figure 4B). Similar trends of CDC activity mediated by these conjugates were also observed in other EGFR‐positive cells, including A549 and SW480 cells (Figure S7, Supporting Information).

**Figure 5:**
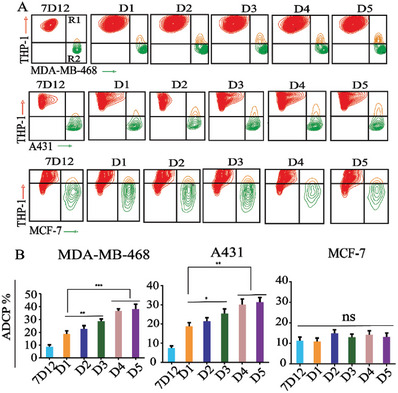
In vitro ADCP assays. A) Flow cytometry assays and B) the corresponding phagocytosis efficiency of target cells treated with 7D12, monovalent conjugate **D1** and multivalent conjugates (**D2**–**D5**). Data are reported as mean ± SD (*n* = 3) and the statistical significance is determined using Student's *t*‐test (two‐tailed). ^*^: *P* < 0.05, ^**^: *P* < 0.01, ^***^: *P* < 0.001.

ADCP is another essential mechanism for killing cancer cells and is mediated by macrophages and therapeutic antibodies. This process is initiated by the interaction of the Fc region of the antibody with the Fc gamma receptors (FcγR) on macrophages, leading to phagocytosis and lysosomal degradation of the target cells. The THP‐1 human monocyte cell line is commonly used in phagocytosis experiments; thus, we employed it as a macrophage cell model to investigate the ADCP activity of the Rha‐7D12 conjugates. In this experiment, the target cells (pre‐stained with green DiO dye) were incubated with the conjugates and subsequently incubated with THP‐1 cells (pre‐stained with orange‐red DiI dye) and pooled human serum (HS), with unmodified 7D12 serving as the control. The phagocytosis efficiency was evaluated by counting the percentage of double‐positive cells using flow cytometry. As presented in **Figure** **5**A, significant numbers of double‐positive cells were observed in the groups treated with the Rha‐7D12 conjugates **D1–D5**, while no such phenomenon was observed in the 7D12‐treated cell group. The phagocytosis efficiency of MDA‐MB‐468 cells induced by Rha‐7D12 conjugates **D1–D5** was 18.6%, 22.7%, 28.6%, 36.6%, and 40.3%, respectively. Similarly, in the A431 cell groups, ≈17.3%, 20.2%, 25.6%, 29.7%, and 31.9% of the cells were phagocytized in the presence of conjugates **D1–D5** (Figure 5B). No significant double‐positive cells were detected in the EGFR‐negative MCF‐7 cells treated with 7D12 or the Rha‐7D12 conjugates. ADCP activities induced by Rha‐7D12 conjugates were also observed in other EGFR‐overexpressing cancer cells, such as A549, SW480, and HT‐29 cells (Figure S8, Supporting Information). Similar to the CDC activity, the ADCP efficiency of the multivalent Rha‐7D12 conjugates **D1–D5** was significantly higher than that mediated by the monovalent conjugate **D1**. Furthermore, the ADCP activity was positively correlated with the number of Rha haptens in the conjugates, with **D5** exhibiting the most potent ADCP efficiency among the conjugates. Therefore, we selected conjugate **D5** for further studies due to its tumor‐targeting ability and superior CDC and ADCP activities in vitro.

**Figure 6:**
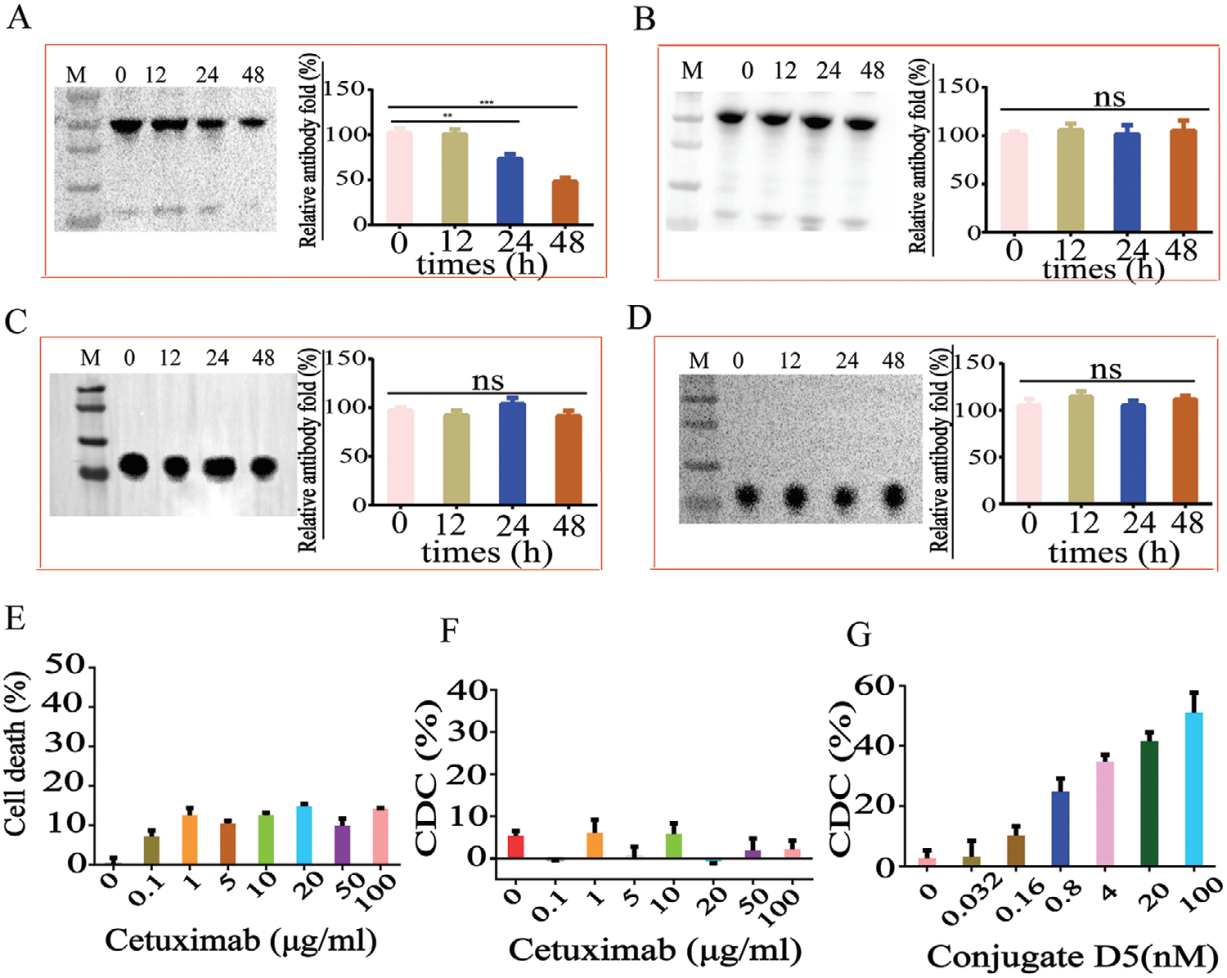
PRSS degradation assays in cetuximab‐resistant tumor cells; Immunoblotting characterization at different time points (left) and the corresponding gray level quantitative analysis (right). A) levels of cetuximab in the HT‐29 cell culture supernatant; B) levels of cetuximab in fresh medium; C) levels of multivalent conjugate **D5** in the HT‐29 cell culture supernatant; D) levels of multivalent conjugate **D5** in fresh medium; E) percentage of HT‐29 cells death after treatment with different concentrations of cetuximab; F,G) percentage of HT‐29 cells death under CDC condition after treating the cells with different concentrations of cetuximab or conjugate **D5**; Data are reported as mean ± SD (*n* = 3).

Cetuximab was the first approved anti‐EGFR antibody used to treat EGFR positive colorectal cancer (CRC) and squamous cell carcinoma of the head and neck (SCCHN) in clinics.^[^ ^10^ ^]^ Recently, PRSS1‐mediated cetuximab resistance has been discovered as a new mechanism in the treatment of colorectal cancer.^[^ ^15^ ^]^ PRSS is secreted by tumor cells and can cleave the mAbs at a site between the variable heavy chain region and constant heavy chain region. Due to the molecular structure difference between nanobodies and full‐length antibodies, nanobody‐based therapeutics may be resistant to this proteolytic degradation. To test this hypothesis, we selected the Rha‐7D12 conjugate **D5** for this study, using cetuximab‐resistant EGFR‐positive HT‐29 cells with aberrant PRSS expression as a model. We collected the supernatant derived from HT‐29 cell culture and co‐cultured it with cetuximab and conjugate **D5**, respectively. The samples were collected at different time points for immunoblotting characterization. Meanwhile, fresh medium without PRSS was incubated with cetuximab or conjugate **D5** as a control. As shown in **Figure** **6**A,B, ≈50% of the cetuximab present in the HT‐29 cell culture supernatant had been cleaved in 48 h, whereas the percentage of cleaved cetuximab in fresh medium was negligible. By contrast, no obvious degradation of conjugate **D5** was observed either in HT‐29 cell culture supernatant (Figure 6C) or fresh medium (Figure 6D). This result demonstrated that conjugate **D5** was resistant to PRSS cleavage.

Complement regulatory proteins expressed on tumor cells, such as CD46, CD55, and CD35, can inhibit the CDC activity of anti‐tumor antibodies, which is another obstacle restricting the clinical efficacy of antibody‐based immunotherapy for cancer treatment.^[^ ^5^, ^27^ ^]^ For this reason, cetuximab is unable to trigger CDC cytotoxicity, one of the most powerful cancer cell killing mechanisms, to promote cancer cell death. As confirmed in Figure 6E,F, cetuximab exhibited no cell‐killing activity in HT‐29 cells under CDC conditions. By contrast, conjugate **D5** displayed dose‐dependent CDC activity in the HT‐29 cell model (Figure 6G). At 100 nm, conjugate **D5** was capable of triggering powerful CDC cytotoxicity to promote cancer cell death up to 49.3%. These results verified that the multivalent Rha‐nanobody conjugate **D5** was a new entity capable of overcoming cetuximab resistance induced by PRSS. Notably, the multivalent Rha haptens allowed conjugate **D5** to recruit more anti‐Rha antibodies to promote CDC cytotoxicity to destroy cancer cells, which cetuximab lacks.

**Figure 7:**
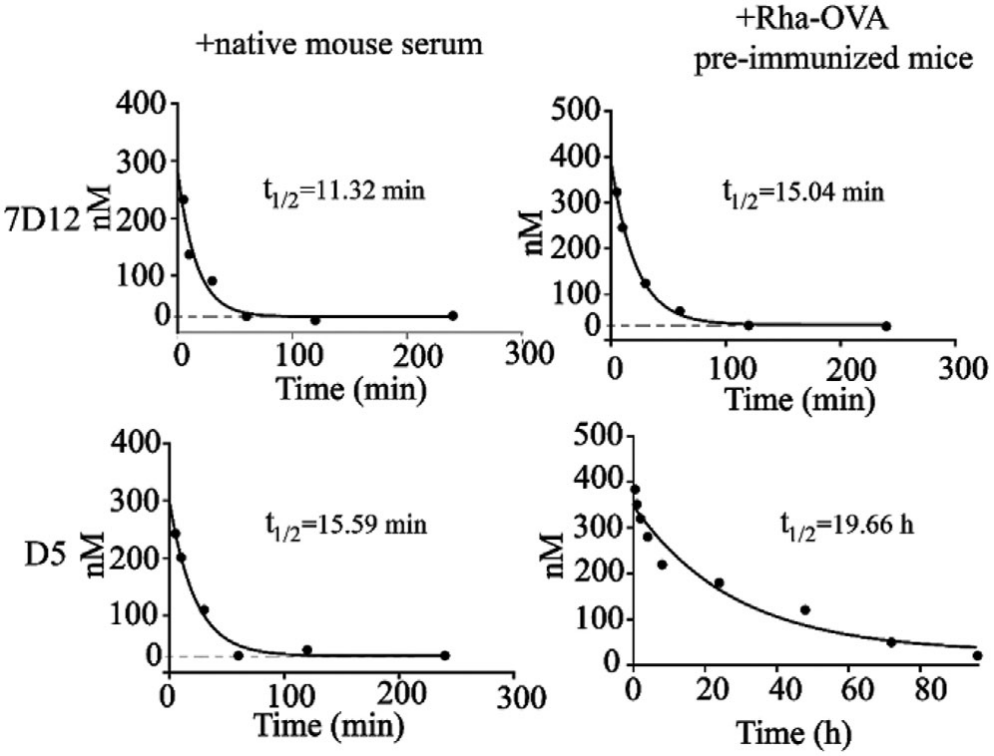
Pharmacokinetics evaluation of conjugate **D5** and 7D12 in native normal mice and in mice containing high titers of endogenous anti‐Rha antibodies.

The poor pharmacokinetics of nanobodies have hindered their clinical application as a therapeutic agent in cancer immunotherapy. The small size and lack of an Fc domain result in rapid clearance from systemic circulation, leading to short half‐lives. To address this issue, we investigated the pharmacokinetic profile of conjugate **D5** in a mouse model. We injected native nanobody 7D12 or conjugate **D5** intravenously into the tail vein of Balb/c mice or Balb/c mice pre‐immunized with Rha‐OVA conjugate (Figure S9, Supporting Information). Serum samples were collected at different time points, and the nanobody concentration was determined by enzyme‐linked immunosorbent assays (ELISAs) according to the standard curves (Figure S10, Supporting Information). As shown in **Figure** **7**, in native mouse serum, both 7D12 and conjugate **D5** showed similar and very short half‐lives, which were 11.32 and 15.59 min, respectively. However, in Balb/c mice containing high levels of endogenous anti‐Rha antibodies, the half‐life of conjugate **D5** reached 19.66 h, which was 104‐fold higher than the unmodified nanobody 7D12. This significant improvement in half‐life was attributed to the ability of conjugate **D5** to effectively bind with anti‐Rha antibodies in vivo and form a large immunocomplex that could avoid rapid renal clearance, leading to dramatically improved half‐life. Additionally, we presumed that that the Fc‐domain of anti‐Rha antibodies displayed on the immunocomplex might bind to the neonatal Fc receptor (FcRn) and utilize the FcRn salvage recycling pathway, similar to full‐length mAb, to improve their pharmacokinetics. This result was consistent with previous observations, which showed that endogenous anti‐DNP antibodies were able to improve the half‐lives of nanobodies or therapeutic peptides.^[^ ^21^, ^28^ ^]^

**Figure 8:**
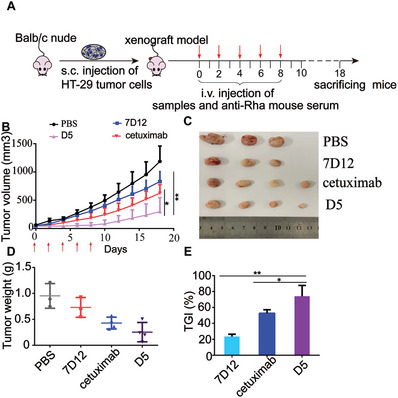
Evaluation of the in vivo antitumor efficacy of cetuximab and conjugate **D5**. A) Experimental design for antitumor activity of xenograft HT‐29 tumors in Balb/c nude mice. B) The tumor volumes of the mice in each group, with each arrow representing one treatment. C) Images D) weight of the tumors isolated from the mice in four groups at the end of the experiment. E) Tumor growth inhibition (TGI) of the treated mice in different groups. TGI was calculated by tumor weights. Data are reported as mean ± SD (*n* = 3 or 4) and the statistical significance is determined using Student's *t*‐test (two‐tailed)., ^*^: *P*< 0.05, ^**^: *P*< 0.01.

Since conjugate **D5** exhibited superior in vitro antitumor activity, excellent in vivo pharmacokinetics, and the ability to overcome cetuximab resistance, **D5** was further evaluated for its in vivo antitumor activity using a xenograft mice model. To establish the mouse model, HT‐29 cells, which are resistant to cetuximab, were subcutaneously injected into the left flank of Balb/c nude mice. Four groups of mice were treated with PBS/pooled anti‐Rha mouse serum, 7D12/pooled anti‐Rha mouse serum, conjugate **D5**/pooled anti‐Rha mouse serum, and cetuximab (1 mg per mouse)^[^ ^15^ ^]^ via i.v. injection every two days for a total of five times. During the treatment and ten days after treatment cessation (18 days total), the tumor volume and body weight were monitored every two days using a caliper. As shown in **Figure** **8**B, tumor growth in the group treated with conjugate **D5** was significantly inhibited during the treatment period and significantly delayed even after stopping treatment. In contrast, the unmodified 7D12 treatment group only displayed minor tumor inhibition, while the cetuximab treatment group exhibited a modest tumor growth inhibition (TGI). To further confirm these results, the tumors were excised at the end of the experiment. As shown in Figure 8C–E, 7D12 exhibited a TGI of only 23.4%, cetuximab exhibited a moderate TGI of 52.7%, while conjugate **D5** remarkably suppressed tumor growth with a TGI of 74.1%. The reduced anti‐tumor efficacy observed in the cetuximab treatment group might be partly attributed to its failure to trigger CDC, despite the Balb/c nude mice model maintaining a normal complement system. These results clearly demonstrated that the multivalent conjugate **D5** exhibited greatly improved antitumor efficacy with the aid of anti‐Rha antibodies, which was attributed to its enhanced Fc‐effector functions and its ability to evade anti‐PRSS degradation.

To assess the toxicity of conjugate **D5**, the body weights of the mice were continuously monitored over the treatment duration. No significant weight changes were observed in all groups (Figure S11, Supporting Information). Additionally, main tissues were dissected for hematoxylin and eosin (H&E) staining, the results of which revealed no inflammation or necrotic regions in the organs (Figure S12, Supporting Information). Furthermore, evaluation of renal‐related indices, such as creatinine (CREA), urea (UREA), and uric acid (UA) levels, indicated that no obvious renal toxicity occurred in any of the groups (Figure S13, Supporting Information). Taken together, these findings suggest that conjugate **D5** was essentially non‐toxic.

## 3 Conclusion

In this study, we designed a series of multivalent Rha‐modified nanobody conjugates with the aim to further optimize the ability of nanobody‐based antibody‐recruiting molecules to recruit endogenous antibodies, which would promote the cancer cell killing by the innate immune cells. It was found that the hexadecameric conjugate **D5** was able to trigger the most potent Fc‐mediated innate immunity to promote cancer cell death, highlighting its potential for cancer immunotherapy. Moreover, we demonstrated that the multivalent Rha‐7D12 conjugate **D5** is completely resistant to enzymatic degradation caused by PRSS. This enzyme is aberrantly expressed in the tumor microenvironment of mCRC patients and has been shown to degrade cetuximab, making PRSS a newly discovered cetuximab resistance mechanism. Of note, conjugate **D5** is capable of triggering potent in vitro CDC cytotoxicity to promote cancer cell death, whereas cetuximab is totally devoid of inducing CDC cytotoxicity due to the complement regulatory proteins expressed on tumor cells. The remarkable antitumor activity of conjugate **D5** was also demonstrated in a cetuximab‐resistant tumor cell derived xenograft mice model.

It is notable to mention that previous studies have shown that 7D12 nanobody was able to inhibit EGF activation by binding to small epitope region of EGFR domain III. This nanobody was found to effectively bind and block EGFR with all known acquired resistance mutations. We demonstrated in this study that the multivalent Rha‐7D12 conjugate **D5**, which was resistant to PRSS degradation, had significantly enhanced Fc effector functions, and had the potential to target acquired resistance mutations of EGFR, is a new modality that has the potential for clinical transformation in cancer immunotherapy.

## 4 Experimental Section

### Materials

All chemicals were obtained from commercial resource and used without further purification. All solvents were dried and purified by known procedures and freshly distilled under nitrogen from appropriate drying agents prior to use. The products were isolated by column chromatography on silica gel (200‐300 mesh or 100–200 mesh) by using petroleum ether (30–60 °C), ethyl acetate, dichloromethane, methyl alcohol as eluents. Dulbecco's Modified Eagle Medium (DMEM, Cat. No. SH30022.01), Roswell Park Memorial Institute medium (1640 medium, Cat. No. SH30809.01), Phosphate‐buffered saline (PBS), Fetal Bovine Serum (FBS, Cat. No. SH30084.03), Penicillin‐Streptomycin solution (Cat. No. SV30010) and HiPrep Desalting column were all from GE Healthcare. Anti‐Rha antibody rabbit serum was obtained from in the previous work. The BCA kit (Cat. No. P_oo10_), MYC‐tag Rabbit Polyclonal antibody, and CCK8 assay kit were purchased from Beyotime. Alexa Fluor 647‐conjugated goat anti‐rabbit IgG(H+L)secondary antibody, HRP‐ conjugated goat anti‐human IgG, Dylight 488‐conjugated goat anti‐rabbit IgG(H+L)secondary antibody, and Dylight 488‐conjugated goat anti‐human secondary antibodies were purchased from Abcam. Normal human serum complement was from Quidel (Cat. No. A_113_). The human serum samples were obtained from Wuxi Cancer Institute and was approved by the Ethics Committee of Affiliated Hospital of Jiangnan University (the Reference Number: JUN20220901IRB22). Rabbit serum containing a high level of anti‐Rha antibodies was prepared by the lab. All the other reagents were purchased commercially. Instrumentation Reaction progress and product mixtures were routinely monitored by Thin layer chromatography (TLC) using TLC SiO_2_ sheets, the compounds were visualized under ultraviolet light or 5% H_2_SO_4_/EtOH. ^1^H NMR, ^13^C NMR spectra were recorded on a Bruker AVANCE III 400 or 600 spectrometer, Mass spectra (ESI) were recorded on a Bruker Amazon SL, MALDI‐TOF/TOF were recorded on a Bruker ultrafleXtreme. Flow cytometry analyses of cells were conducted with BD FACSArica III. Confocal laser scanning microscopy (CLSM) images were taken on a Carl Zeiss LSM880 Confocal Microscope (Carl Zeiss, LSM880, Germany). HPLC condition for analysis of the purities of compounds 1–5: Diamonsil 5 µm C18 250×4.6 mm, MeCN 95−60% in 20 min, 220 nm.

### Cell Culture

The human TNBC cell line MDA‐MB‐468, human squamous carcinoma cell line A431, human breast cancer cell line MCF‐7, human non‐small cell lung cancer cells A549. All the above lines were cultured in DMEM medium supplemented with 10% FBS and 1% penicillin‐streptomycin. Human colorectal cancer cells SW480 were cultured in 1640 medium supplemented with 10% FBS and 1% penicillin‐streptomycin, Human colorectal cancer cells HT‐29 were cultured in McCoy's 5A medium supplemented with 10% FBS and 1% penicillin‐streptomycin at 37 °C in a 5% CO_2_ humidified atmosphere.

### Animals

Balb/c mice (female, 6–8 weeks old) and balb/c nude mice (female, 4–6 weeks old) were from Shanghai SLAC Laboratory Animal Co. Ltd. All animal procedures were conducted according to the guidelines and protocols were approved by the Institutional Animal Care and Use Committee of Jiangnan University (JN. No20220330b0300620[095] and JN.No20220615b0200910[212]).

### SrtA‐Mediated Ligation for the Synthesis of Conjugates (**D1** to **D5**)

Triglycine‐functionalized multivalent rhamnose dendrimers (compounds **1**–**5**, 500 µm), nanobody 7D12 (20 µm), and eSrtA (5 µm) were mixed in 1 mL of reaction buffer (50 mm Tris/HCl, 150 mm NaCl, 5 mm CaCl2, pH 7.5), the mixture was conducted at 16 °C for 2 h with constant shaking at 200 rpm. Then, nickel‐magnetic beads (500 µL) were added into the mixture to capture and remove eSrtA and unreacted nanobody 7D12 bearing 6xHis tag. The supernatant containing the desired conjugates were collected and subjected to purification with a Sephadex G25 column to remove small molecular rhamnose dendrimers to obtain pure protein conjugates **D1** to **D5**.

### Evaluation of EGFR Expression on Different Cell Lines

Cells were cultured and detached with trypsin‐EDTA and then resuspended and diluted in flow cytometry buffer (2% BSA in PBS) to 5×105 cells/mL. 100 µL of these cells were added into tubes containing 100 µL of PBS or cetuximab (final concentration:100 nm). After the incubation on ice for 30 min and washing two times with flow cytometry buffer (2×500 µL), cells were treated with 100 µL of Dylight 488‐conjugated goat anti‐human secondary antibodies (1% diluted in flow cytometry buffer) on ice for 30 min. After another washing step, cells were resuspended in 200 µL of flow cytometry buffer, they were analyzed using an BD FACSArica III, and the data analyses were performed with the FlowJo software.

### Binding Specificity of Rha‐Nanobody Conjugates with Cancer Cells Assay

Cells were cultured and detached with trypsin‐EDTA and then resuspended and diluted in flow cytometry buffer to 5×105 cells/mL. 100 µL of these cells were added into tubes containing 100 µL of conjugates (**D1** to **D5**) or nanobody 7D12 (final concentration:100 nm). After the incubation on ice for 30 min and washing two times, cells were treated with 100 µL of MYC‐tag Rabbit Polyclonal antibody (1% diluted in flow cytometry buffer) on ice for 30 min. After another washing step, cells were treated with 100 µL of DyLight 488‐conjugated goat anti‐rabbit secondary antibodies (1% diluted in flow cytometry buffer) on ice for 30 min, washed with flow cytometry buffer (2×500 µL) and then resuspended in 200 µL of flow cytometry buffer. Finally, they were analyzed using an BD FACS C6, and the data analyses were performed with the FlowJo software.

### Anti‐Rha Antibody Recruiting Capability Assay

Cells were cultured and detached with trypsin‐EDTA and then resuspended and diluted in flow cytometry buffer to 5×10^5^ cells/mL. 100 µL of these cells were added into tubes containing 100 µL of conjugates (**D1** to **D5**) or nanobody 7D12 (final concentration: 100 nm). After incubation on ice for 30 min and washing two times, cells were treated with 100 µL of diluted rabbit serum (1% diluted in flow cytometry buffer) on ice for 30 min. After another washing step, cells were treated with 100 µL of Alexa Fluor 647‐conjugated goat anti‐rabbit IgG(H+L)secondary antibody (0.05% diluted in flow cytometry buffer) on ice for 30 min, washed with flow cytometry buffer (2×500 µL) two times and then resuspended in 200 µL of flow cytometry buffer. Finally, they were analyzed using an BD FACSArica III, and the data analyses were performed with the FlowJo software.

### Confocal Laser Scanning Microscopy Assay

Cells were seeded in a 24‐well plate at a density of 8×10^3^ cells per well and allowed to attach for 12 h, the old medium was removed and the cells were fixed with 4% paraformaldehyde for 10 min, and blocked at 37 °C for 1 h (1% BSA in PBS). Then, cells were treated with 100 nm protein samples (conjugate D1 to D5, and 7D12) at 37 °C for 1 h, washed with PBS, cells were then incubated with 500 µL of diluted rabbit serum (1% diluted in 1% BSA) at 37 °C for 1 h. After another washing step, cells were treated with 500 µL of Alexa Fluor 647‐conjugated goat anti‐rabbit IgG (H+L) secondary antibody (0.2% diluted in 1% BSA) at 37°C for 1 h, washed with PBS, after permeabilization with 0.1% TritonX‐100 (in 1% BSA), cells were treated with a drop of antifade mounting medium (containing 4′,6‐diamidino‐2‐phenylindole (DAPI)) and finally imaged using a confocal microscope.

### CDC Assay

All cells were seeded in a 96‐well plate at a density of 4×10^3^ cells per well and allowed to attach for 12 h, the old culture media were removed and cells were incubated with 100 nm protein samples (conjugates **D1** to **D5** and 7D12) at 37 °C for 1 h, washed with PBS, cells were then incubated with 50 µL of diluted human serum (40% in media, the final concentration 20%) and 50 µL of diluted human complement (2% in media, the final concentration 1%) and incubated for 6–8 h. The cell maximum killing was achieved by adding 100 µL of 1% Triton X‐100 to the cell culture. The cell viability was measured using a CCK8 assay kit on a microplate reader at 450 nm wavelength. Cell cytotoxicity was calculated by the following equation. The protocol and usage of human samples has been approved by the Ethics Committee of Jiangnan University (No. LS2019047).

(1) $$\mathrm{CDC}\;\left(\%\right)=\left(1-\frac{A\left(\mathrm{experimental}\right)-A\left(\mathrm{maximum}\right)}{A\left(\mathrm{negative}\right)-A\left(\mathrm{maximum}\right)}\right)\;\times 100\%$$

Where A(negative) is the OD450 value of cells treated with PBS; A(maximum) is the OD450 value of cells completely lysed with 1% Triton X‐100; A(experimental) is the OD450 value of cells treated with protein samples (7D12 and conjugates **D1** to **D5**).

### ADCP Assay

DiO‐prestained target cells (green) were seeded in 24‐well plates at a concentration of 3×10^4^ cells/well, and then treated with 100 µL of 100 nM protein samples (conjugates **D1** to **D5** and 7D12). After 1 h of incubation at 37 °C, target cells were washed with PBS and co‐cultured with 1.5×105 DiI‐prestained THP‐1 cells (orange–red) in the presence of 20% pooled human serum for another 4 h. Subsequently, the medium containing THP‐1 cells were collected in 1.5 mL Eppendorf tubes and the remaining target cells were detached with trypsin and added to the same tubes. After another washing step to remove trypsin, cells were collected for flow cytometry assays. The rates of ADCP were determined by detecting the percentage of double‐positive cells to analysis the phagocytosis efficiency by THP‐1 cells.

(2) $$\mathrm{ADCP}\;\%\;=\left(\frac{R1}{R1+R2}\right)\;\times 100\%$$

where R1 is the percentage of double‐positive cells; R2 is the percentage of remaining target cells.

### PRSS1 Proteolytic Cleavage of mAbs Assay

The study collected the supernatant derived from HT‐29 cell culture and co‐cultured it with cetuximab and conjugate **D5**, respectively. The samples were collected at different time points (0, 12, 24, 48 h) for immunoblotting characterization. Meanwhile, fresh medium without PRSS1 was incubated with cetuximab or conjugate **D5** as a control.

### In Vivo Pharmacokinetics Study

The half‐life of conjugate **D5** were evaluated in normal mice and Rha‐OVA pre‐immunized Balb/c mice. To prepare pre‐immunized Balb/c mice, Rha‐OVA conjugate (2 mg mL^−1^ in PBS) were 1:1 mixed with Freund's incomplete adjuvant and subcutaneously injected (100 µg of Rha‐OVA/injection) into the back of balb/c mice at day 1, 7, and 14. Serum samples at day 0 and 21 were collected for anti‐Rha IgG titer evaluation. For pharmacokinetics study, nanobody samples (nanobody 7D12 or conjugate **D5**) were injected into the tail vein of a group of 3 Balb/c mice or Rha‐OVA pre‐immunized Balb/c mice respectively. Serum samples were collected at different time points to determine the concentrations of nanobody 7D12 or conjugate **D5** samples using ELISA. For standard curves preparation: High binding 96‐well plates were coated with human recombinant EGFR (2 µg mL^−1^) at 4 °C for overnight and at 37 °C for 1 h, washed with PBST three times and blocking with 2% BSA at r.t for 2 h, subsequently, the 96‐well plates were incubated with different concentrations nanobody 7D12 or conjugate **D5** (0.2–10 nm) at 37 °C for 2 h, washed with PBST, and were treated with HRP‐conjugated anti‐myc tag at 37 °C for 1 h. Finally, the results were determined by TMB kit. For sample concentration determination: High binding 96‐well plates were coated with human recombinant EGFR (2 µg mL^−1^) at 4 °C for overnight and at 37 °C for 1 h, washed with PBST three times and blocking with 2% BSA at r.t for 2 h, subsequently, the 96‐well plates were incubated with mice serum of different time points (diluted with 1:100 in PBS) at 37 °C for 2 h, washed with PBST, and were treated with HRP‐conjugated anti‐myc tag at 37 °C for 1 h. finally, the results were determined by TMB kit.

### In Vivo Anti‐Tumor Evaluation

First, enough anti‐Rha mouse sera were collected and pooled as the source of endogenous anti‐Rha antibodies. For xenograft model establishment, ≈2×106 HT‐29 cells in 100 µL of sterilized PBS buffer solution and were subcutaneously injected into the left flank of Balb/c nude mice. After the mice with 40–80 mm^3^ of tumor, and were randomly divided into four groups, and each group treated via i.v. injection with 50 µL of PBS and 50 µL of pooled anti‐Rha mouse serum, 50 µL of 7D12 (40 µmol L^−1^ in PBS) and 50 µL of pooled anti‐Rha mouse serum, 50 µL of conjugate D5 (40 µmol L^−1^ in PBS) and 50 µL of pooled anti‐Rha mouse serum and 100 µL of cetuximab (10 mg mL^−1^ in PBS, 1 mg per mouse). Treatments were given every two days for total five times, during the treatment and ten days after treatment cessation, the tumor volume and body weight were measured by vernier caliper and digital scale every two days respectively, and the volume was calculated using following equation: V = (length×width2)/2.

### In Vivo Toxicity Assay

For renal toxicity evaluation, sera were collected for creatinine (CREA), urea (UREA), and uric acid (UA) determination. For hematoxylin and eosin (H&E) assays, the heart, liver, spleen, lung, and kidney tissues from sacrificed mice were immediately fixed with 10% formalin for subsequent slices making. Thereafter, the slices were stained by H&E, and immaged on microscope.

### Statistical Analysis

All quantitative data were presented as mean ± SD from triplicate measurements and were analyzed statistically using a Student's *t*‐test (two‐tailed) to compare two groups of independent samples using GraphPad Prism 6.0 software, unless otherwise noted. A value of *P* < 0.05 was considered statistically significant.

## Conflict of Interest

The authors declare no conflict of interest.

## Supporting information

Supporting Information

## Data Availability

The data that support the findings of this study are available in the supplementary material of this article.

## References

[1] Y. Yarden , M. X. Sliwkowski , Nat. Rev. Mol. Cell Biol. 2001, 2, 127. 11252954 10.1038/35052073

[2] a) N. Normanno , A. De Luca , C. Bianco , L. Strizzi , M. Mancino , M. R. Maiello , A. Carotenuto , G. De Feo , F. Caponigro , D. S. Salomon , Gene 2006, 366, 2; 16377102 10.1016/j.gene.2005.10.018

[3] A. Dokala , S. S. Thakur , Oncogene 2017, 36, 2337. 27775071 10.1038/onc.2016.393

[4] E. Martinelli , R. De Palma , M. Orditura , F. De Vita , F. Ciardiello , Clin. Exp. Immunol. 2009, 158, 1. 10.1111/j.1365-2249.2009.03992.x PMC2759052 19737224

[5] a) M. Dechant , W. Weisner , S. Berger , M. Peipp , T. Beyer , T. Schneider‐Merck , J. J. Lammerts van Bueren , W. K. Bleeker , P. W. Parren , J. G. van de Winkel , T. Valerius , Cancer Res. 2008, 68, 4998; 18593896 10.1158/0008-5472.CAN-07-6226

[6] A. Bardelli , P. A. Janne , Nat. Med. 2012, 18, 199. 22310681 10.1038/nm.2646

[7] a) B. O. Van Emburgh , S. Arena , G. Siravegna , L. Lazzari , G. Crisafulli , G. Corti , B. Mussolin , F. Baldi , M. Buscarino , A. Bartolini , E. Valtorta , J. Vidal , B. Bellosillo , G. Germano , F. Pietrantonio , A. Ponzetti , J. Albanell , S. Siena , A. Sartore‐Bianchi , F. Di Nicolantonio , C. Montagut , A. Bardelli , Nat. Commun. 2016, 7, 13665; 27929064 10.1038/ncomms13665 PMC5155160

[8] a) T. Price , A. Ang , M. Boedigheimer , T. W. Kim , J. Li , S. Cascinu , P. Ruff , A. Satya Suresh , A. Thomas , S. Tjulandin , M. Peeters , Cancer Biol. Ther. 2020, 21, 891; 33026965 10.1080/15384047.2020.1798695 PMC7583702

[9] F. Braig , M. März , A. Schieferdecker , A. Schulte , M. Voigt , A. Stein , T. Grob , M. Alawi , D. Indenbirken , M. Kriegs , E. Engel , U. Vanhoefer , A. Grundhoff , S. Loges , K. Riecken , B. Fehse , C. Bokemeyer , M. Binder , Oncotarget 2015, 6, 12035. 26059438 10.18632/oncotarget.3574 PMC4494921

[10] A. Bagchi , J. N. Haidar , S. W. Eastman , M. Vieth , M. Topper , M. D. Iacolina , J. M. Walker , A. Forest , Y. Shen , R. D. Novosiadly , K. M. Ferguson , Mol. Cancer Ther. 2018, 17, 521. 29158469 10.1158/1535-7163.MCT-17-0575 PMC5925748

[11] X. Zhuang , Z. Wang , J. Fan , X. Bai , Y. Xu , J. J. Chou , T. Hou , S. Chen , L. Pan , Nat. Commun. 2022, 13, 4431. 35907884 10.1038/s41467-022-32159-6 PMC9338999

[12] C. Montagut , G. Argiles , F. Ciardiello , T. T. Poulsen , R. Dienstmann , M. Kragh , S. Kopetz , T. Lindsted , C. Ding , J. Vidal , J. Clausell‐Tormos , G. Siravegna , F. J. Sanchez‐Martin , K. Koefoed , M. W. Pedersen , M. M. Grandal , M. Dvorkin , L. Wyrwicz , A. Rovira , A. Cubillo , R. Salazar , F. Desseigne , C. Nadal , J. Albanell , V. Zagonel , S. Siena , G. Fumi , G. Rospo , P. Nadler , I. D. Horak , et al., JAMA Oncol. 2018, 4, e175245. 29423521 10.1001/jamaoncol.2017.5245 PMC5885274

[13] a) S. Arena , G. Siravegna , B. Mussolin , J. D. Kearns , B. B. Wolf , S. Misale , L. Lazzari , A. Bertotti , L. Trusolino , A. A. Adjei , C. Montagut , F. Di Nicolantonio , R. Nering , A. Bardelli , Sci. Transl. Med. 2016, 8, 324ra314; 10.1126/scitranslmed.aad5640 26843189

[14] F. Braig , M. Kriegs , M. Voigtlaender , B. Habel , T. Grob , K. Biskup , V. Blanchard , M. Sack , A. Thalhammer , I. Ben Batalla , I. Braren , S. Laban , A. Danielczyk , S. Goletz , E. Jakubowicz , B. Markl , M. Trepel , R. Knecht , K. Riecken , B. Fehse , S. Loges , C. Bokemeyer , M. Binder , Cancer Res. 2017, 77, 1188. 28031227 10.1158/0008-5472.CAN-16-0754

[15] Z. Tan , L. Gao , Y. Wang , H. Yin , Y. Xi , X. Wu , Y. Shao , W. Qiu , P. Du , W. Shen , L. Fu , R.u Jia , C. Zhao , Y. Zhang , Z. Zhao , Z. Sun , H. Chen , X. Hu , J. Xu , Y. Wang , Sci. Adv. 2020, 6, 1. 10.1126/sciadv.aax5576 PMC6938705 31911942

[16] C. Hamers‐Casterman , T. Atarhouch , S. Muyldermans , G. Robinson , C. Hamers , E. B. Songa , N. Bendahman , R. Hamers , Nature 1993, 363, 446. 8502296 10.1038/363446a0

[17] S. Muyldermans , Ann. Rev. Biochem. 2013, 82, 775. 23495938 10.1146/annurev-biochem-063011-092449

[18] K. M. Shokat , P. G. Schultz , J. Am. Chem. Soc. 1991, 113, 1861.

[19] O. Oyelaran , L. M. McShane , L. Dodd , J. C. Gildersleeve , J. Proteome Res. 2009, 8, 4301. 19624168 10.1021/pr900515y PMC2738755

[20] R. T. Sheridan , J. Hudon , J. A. Hank , P. M. Sondel , L. L. Kiessling , ChemBioChem 2014, 15, 1393. 24909955 10.1002/cbic.201402019 PMC4205123

[21] a) H. Hong , Z. Zhou , K. Zhou , S. Liu , Z. Guo , Z. Wu , Chem. Sci. 2019, 10, 9331; 32110296 10.1039/c9sc03840j PMC7006623

[22] C. E. Jakobsche , C. G. Parker , R. N. Tao , M. D. Kolesnikova , E. F. Douglass, Jr. , D. A. Spiegel , ACS Chem. Biol. 2013, 8, 2404. 24053626 10.1021/cb4004942 PMC3830660

[23] M. Mammen , S. K. Choi , G. M. Whitesides , Angew. Chem., Int. Ed. 1998, 37, 2754. 10.1002/(SICI)1521-3773(19981102)37:20<2754::AID-ANIE2754>3.0.CO;2-3 29711117

[24] a) A. Uvyn , R. De Coen , M. Gruijs , C. W. Tuk , J. De Vrieze , M. van Egmond , B. G. De Geest , Angew. Chem., Int. Ed. 2019, 58, 12988; 10.1002/anie.201905093 31206941

[25] J. Tintelnot , N. Baum , C. Schultheiss , F. Braig , M. Trentmann , J. Finter , W. Fumey , P. Bannas , B. Fehse , K. Riecken , K. Schuetze , C. Bokemeyer , T. Rosner , T. Valerius , M. Peipp , F. Koch‐Nolte , M. Binder , Mol. Cancer Ther. 2019, 18, 823. 30824613 10.1158/1535-7163.MCT-18-0849

[26] B. Todaro , S. Achilli , B. Liet , E. Laigre , C. Tiertant , D. Goyard , N. Berthet , O. Renaudet , Biomater. Sci. 2021, 9, 4076. 33913968 10.1039/d1bm00485a

[27] Z. Fishelson , N. Donin , S. Zell , S. Schultz , M. Kirschfink , Mol. Immunol. 2003, 40, 109. 12914817 10.1016/s0161-5890(03)00112-3

[28] a) S. Dai , H. Hong , K. Zhou , K. Zhao , Y. Xie , C. Li , J. Shi , Z. Zhou , L. Nie , Z. Wu , J. Med. Chem. 2021, 64, 4947; 33825469 10.1021/acs.jmedchem.1c00032

