# Supplementary material for: Multivalent Rhamnose‐Modified EGFR‐Targeting Nanobody Gains Enhanced Innate Fc Effector Immunity and Overcomes Cetuximab Resistance via Recruitment of Endogenous Antibodies
Source: Adv Sci (Weinh). 2024 Jan 29;11(13):2307613. doi: 10.1002/advs.202307613 (PMC10987161; doi:10.1002/advs.202307613)
Supplement: Supplementary file 1 — Supporting Information [file ADVS-11-2307613-s001.pdf]

ADVANCED SCIENCE
Open Access

Supporting Information

for *Adv. Sci.*, DOI 10.1002/advs.202307613

Multivalent Rhamnose-Modified EGFR-Targeting Nanobody Gains Enhanced Innate Fc Effector Immunity and Overcomes Cetuximab Resistance via Recruitment of Endogenous Antibodies

Yanchun Li, Han Lin, Haofei Hong, Dan Li, Liang Gong, Jie Zhao, Zheng Wang and Zhimeng Wu**

Supporting Information
©Wiley-VCH 2021
69451 Weinheim, Germany

Multivalent Rhamnose-Modified EGFR-targeting Nanobody Gains Enhanced Innate Fc Effector Immunity and Overcomes Cetuximab Resistance via Recruitment of Endogenous Antibodies

Yanchun Li, Han Lin, Haofei Hong *, Dan Li, Liang Gong, Jie Zhao, Zheng Wang, Zhimeng Wu*

Abstract: Cetuximab resistance is a significant challenge in cancer treatment, requiring the development of novel therapeutic strategies. In this study, we synthesized a series of multivalent rhamnose (Rha)-modified nanobody conjugates and investigated their antitumor activities and their potential to overcome cetuximab resistance. Structure-activity relationship studies revealed that the multivalent conjugate **D5**, bearing sixteen Rha haptens, exhibited the most potent innate Fc effector immunity *in vitro* and excellent *in vivo* pharmacokinetics by recruiting endogenous antibodies. Notably, we found that the optimal conjugate **D5** represents a novel entity capable of reversing cetuximab-resistance induced by serine protease (PRSS). Moreover, in a xenograft mouse model, conjugate **D5** exhibited significantly improved antitumor efficacy compared to unmodified nanobodies and cetuximab. Our findings suggest that Rha-Nanobody (Nb) conjugates hold promise as a novel therapeutic strategy for the treatment of cetuximab-resistant tumors by enhancing the innate crystallizable fragment (Fc) effector immunity and enhancing the recruitment of endogenous antibodies to promote cancer cell clearance by innate immune cells.

DOI: 10.1002/anie.2023XXXXX

Table of Contents

Chemical Synthesis Procedures for intermediate compound **6**-**11**....S3
Chemical Synthesis Procedures for Rha Derivates compound **1**-**5** S7
Supplementary Figures S12
NMR Spectra....S22
References....S49
Author Contributions....S49

Chemical Synthesis Procedures for intermediate compound 6-11

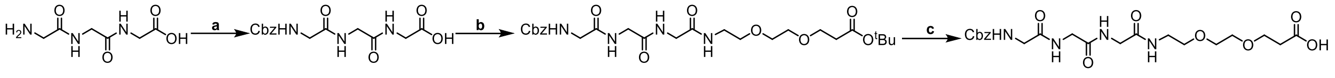

S1 S2 S3 6

Scheme S1: Reagents and conditions: (a) DCM, benzyl chloroformate, 25% Na_2CO_3, 36 h, r.t, 78%; (b) tert-butyl 3-(2-(2-aminoethoxy) ethoxy) propanoate, EDC·HCl, HOBt, TEA, DMF, r.t, 12 h, 74%; (c) 25% TFA/DCM, 0ºC, 2 h, 85%.

Compound S2: To a solution of the compound **S1** (1.9 g, 10 mmol) in DCM (30 mL) and added benzyl chloroformate (2 mL, 15 mmol), 25% Na_2CO_3 (2 g / 8.5 mL). The reaction mixture was stirred at r.t for 36 h, after the completion of the reaction, the reaction was concentrated and extracted with DCM, washed with saturated NaCl solution, the organic layer was dried over anhydrous Na_2SO_4, then was concentrated and give the compound **S2** (2.5 g, 78%). ^{1}H NMR (400 MHz, DMSO-d_6) δ 12.60 (s, 1H), 8.24 (m, 2H), 7.53 (m, 1H), 7.36 (s, 2H), 7.33 – 7.29 (m, 1H), 5.03 (s, 2H), 3.78 – 3.71 (m, 4H), 3.68 (d, J = 6.1 Hz, 2H). MS-ESI (m/z): calculated for 346.10; observed, 346.02, $[M+Na]^+$

Compound S3: To a solution of the compound **S2** (2.3 g, 7.1 mmol) in DMF (20 mL) and added tert-butyl 3-(2-(2-aminoethoxy) ethoxy) propanoate (1.6 g, 7.1 mmol), HOBt (1.2 g, 8.52 mmol), TEA (2 mL, 14 mmol) and EDC·HCl (1.6 g, 8.52 mmol). The reaction mixture was stirred at r.t. for 12 h. After the completion of the reaction, the reaction was concentrated, extracted with DCM, washed with saturated NaCl solution, the organic layer was dried over anhydrous Na_2SO_4, then concentrated and was purified by silica gel chromatography to give the compound **S3** (2.8 g, 74%). ^{1}H NMR (400 MHz, Methanol-d_4) δ 7.43 – 7.27 (m, 5H), 5.13 (s, 2H), 3.92 (s, 2H), 3.88 (s, 2H), 3.85 (s, 2H), 3.71 (t, *J* = 6.2 Hz, 2H), 3.60 (s, 4H), 3.55 (t, *J* = 5.6 Hz, 2H), 3.39 (t, *J* = 5.6 Hz, 2H), 2.49 (t, *J* = 6.2 Hz, 2H), 1.47 (s, 9H). MS-ESI (m/z): calculated for 561.25, observed, 561.05, $[M+Na]^+$

Compound 6: To a solution of the compound **S3** (200 mg, 0.37 mmol) in DCM (6 mL), then 50% TFA/DCM (6 mL) was added dropwise at 0ºC, then the reaction mixture was stirred at 0ºC for 2 h. After the completion of the reaction, the reaction was diluted with toluene (5 mL), and concentrated in vacuo. The residual TFA was removed from the crude residue by azeotroping with toluene (3×5 mL) and was concentrated to give the compound **6** (153mg, 85%). ^{1}H NMR (400 MHz, DMSO-d_6) δ 8.16 (m, 1H), 8.11 (m, 1H), 7.84 (m, 1H), 7.49 (m, 1H), 7.42 – 7.27 (m, 5H), 5.04 (s, 2H), 3.75 (d, *J* = 5.6 Hz, 2H), 3.68 (m 4H), 3.60 (t, *J* = 6.3 Hz, 3H), 3.40 (t, *J* = 5.9 Hz, 6H), 3.21 (m, 2H), 2.44 (t, *J* = 6.3 Hz, 2H). MS-ESI (m/z): calculated for 505.19, observed, 504.99, $[M+Na]^+$

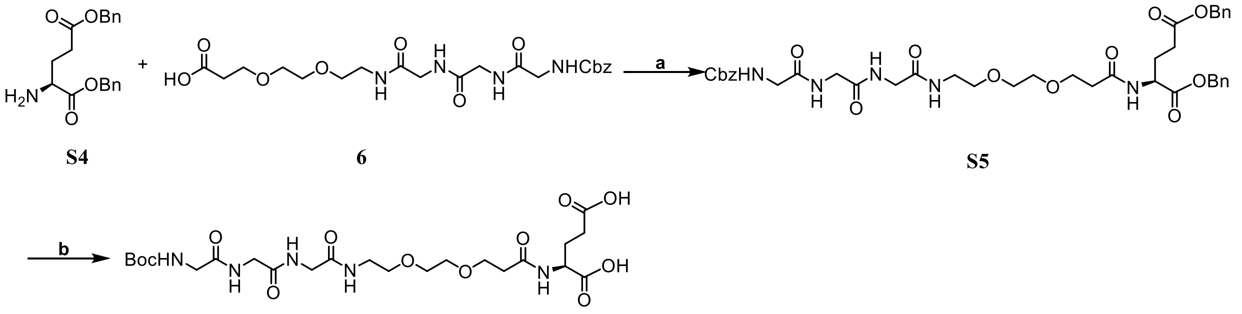

S4 6 S5

7

Scheme S2: Reagents and conditions: (a). EDC·HCl, HOBt, TEA, DCM, r.t, 12 h,73%; (b) (1) MeOH, Pd/C H_2, r.t, 12 h, (2) MeOH, Boc_2O, TEA, r.t, 2 h, 73% (over two steps). Compound **S5**: To a solution of the compound **S4** (2.8 g, 6 mmol) in DCM (30 mL) and added compound **6** (2.5 g, 5 mmol), HOBt (822 mg, 6 mmol), TEA (1.6 mL, 12 mmol) and EDC·HCl (1.1 g, 6 mmol) at 0°C. The reaction mixture was stirred at r.t. for 12 h, after the completion of the reaction, the reaction was diluted with DCM, washed with saturated NaCl solution, the organic layer was dried over anhydrous Na_2SO_4, then concentrated and was purified by silica gel chromatography to give the compound **30** (3 g, 73%). ^{1}H NMR (600 MHz, Methanol-d_4) δ 7.41 – 7.36 (m, 6H), 7.36 – 7.29 (m, 9H), 5.16 (d, *J* = 5.1 Hz, 2H), 5.11 (m, 4H), 4.56 (m, 1H), 3.91 (s, 1H), 3.86 (m, 4H), 3.75 – 3.65 (m, 2H), 3.54 (m, 4H), 3.49 (m, 2H), 3.39 – 3.33 (m, 3H), 2.57 – 2.40 (m, 4H), 2.21 (m, 1H), 2.06 – 1.91 (m, 1H). ^{13}C NMR (150 MHz, Methanol-d_4) δ 172.72, 172.60, 171.71, 171.56, 170.70, 170.21, 157.89, 136.61, 136.13, 135.80, 128.20, 128.17, 128.12, 127.96, 127.95, 127.88, 127.87, 127.84, 127.71, 127.57, 69.90, 69.83, 68.96, 66.75, 66.65, 66.63, 66.04, 51.66, 48.18, 48.04, 47.90, 47.75, 47.61, 47.47, 47.33, 47.19, 43.79, 42.39, 42.02, 39.00, 36.01, 29.76, 26.31. MS-ESI (m/z): calculated for 792.35; observed, 792.08, $[M+H]^+$

Compound 7: To a solution of the compound **S5** (1.5 g, 1.9 mmol) in MeOH (20 mL) and added Pd/C (200 mg). The reaction mixture was stirred at r.t. under H_2 atmosphere for 12 h. After the completion of the reaction, the product mixture was filtered through Celite, washed with MeOH, and then the solvent was concentrated and used for the next step directly without purification. Then the reaction was dissolved in MeOH (20 mL), and added Boc_2O (492 mg, 2.2 mmol), TEA (0.4 mL 3.8 mmol). Then the mixture was stirred at r.t for 12 h, after the completion of the reaction, the reaction was concentrated and washed with Et_2O three times to give the compound **7** (800 mg, 73%). ^{1}H NMR (400 MHz, Methanol-d_4) δ 4.29 (m, 1H), 3.91 (s, 2H), 3.86 (s, 2H), 3.75 (s, 2H), 3.74 – 3.68 (m, 2H), 3.60 (s, 4H), 3.54 (m, 2H), 3.37 (m, 2H), 2.56 – 2.41 (m, 2H), 2.38 – 2.28 (m, 2H), 2.15 (m, 1H), 1.99 – 1.87 (m, 1H), 1.44 (s, 9H). MS-ESI (m/z): calculated for 600.25, observed, 600.09, $[M+Na]^+$

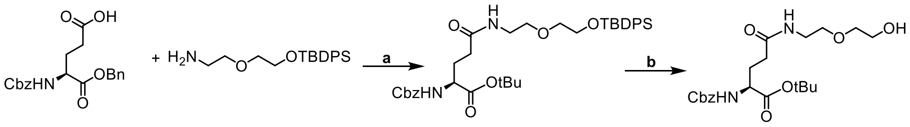

S6 S7 S8 S9

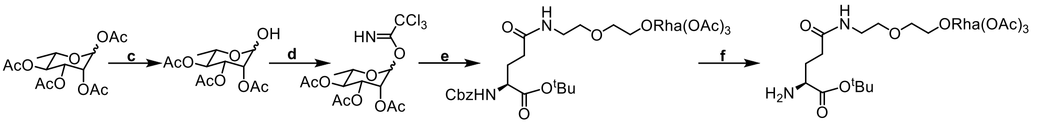

S10 S11 S12 S13 8

Scheme S3: Reagents and conditions: (a) EDC·HCl, HOBt, TEA, DCM, r.t, 12 h,74%; (b) THF, TBAF, AcOH, r.t, 12 h, 89%; (c) MeOH/THF, $(NH_4)_2CO_3$, r.t, 6 h; (d) DCM, CCl_3CN, DBU, 0ºC, 2 h, 72%, (over two steps); (e) compound **S9**, DCM, TMSOTf, 72%; (f) MeOH, Pd/C, H_2, r.t, 12 h, 93%.

Compound S8: To a solution of the compound **S6** (6 g, 18 mmol) in DCM (70 mL) and added compound **S7** (5.1 g, 15 mmol), HOBt (2.5 g, 18 mmol), TEA (4.1 mL, 30 mmol) and EDC·HCl (3.4 g, 18 mmol) at 0°C. The reaction mixture was stirred at r.t. for 12 h, after the completion of the reaction, the reaction was concentrated and extracted with DCM, washed with saturated NaCl solution, the organic layer was dried over anhydrous Na_2SO_4, then concentrated and was purified by silica gel chromatography to give the compound **S8** (8 g, 73%). ^{1}H NMR (400 MHz, Chloroform-d) δ 7.74 – 7.66 (m, 4H), 7.47 – 7.31 (m, 11H), 6.08 (s, 1H), 5.59 (d, *J* = 8.1 Hz, 1H), 5.10 (s, 2H), 4.28 – 4.19 (m, 1H), 3.84 (dd, *J* = 5.7, 4.4 Hz, 2H), 3.58 (dd, *J* = 5.7, 4.5 Hz, 2H), 3.53 (t, *J* = 5.1 Hz, 2H), 3.42 (dd, *J* = 5.2, 3.2 Hz, 2H), 2.25 – 2.13 (m, 3H), 2.00 – 1.88 (m, 1H), 1.75 (s, 2H), 1.08 (s, 9H). MS-ESI (m/z): calculated for, 685.33, observed, 685.11, $[M+Na]^+$

Compound S9: To a solution of the compound **S8** (6 g, 9 mmol) in THF (50 mL), then TBAF (13.5 mmol, 13.5 mL), AcOH (18 mmol, 1.8 g) were added, and the reaction mixture was stirred at r.t for 12 h. After the completion of the reaction, the reaction was concentrated and extracted with DCM, washed with saturated NaCl solution, the organic layer was dried over anhydrous Na_2SO_4, then concentrated and was purified by silica gel chromatography to give the compound **S9** (3.4 g, 89%). ^{1}H NMR (400 MHz, Chloroform-d) δ 7.40 – 7.33 (m, 5H), 6.89 (d, *J* = 5.8 Hz, 1H), 5.72 (d, *J* = 8.1 Hz, 1H), 5.12 (d, *J* = 2.9 Hz, 2H), 4.24 (m, 1H), 3.77 – 3.72 (m, 2H), 3.62 – 3.55 (m, 4H), 3.52 – 3.39 (m, 2H), 3.14 (s, 1H), 2.31 – 2.20 (m, 3H), 2.05 (s, 2H), 1.89 (m, 1H), 1.47 (s, 9H). MS-ESI (m/z): calculated for 447.21, observed, 447.01, $[M+Na]^+$

Compound S13: To a solution of the compound **S10** (3.3 g, 10 mmol) in MeOH/THF (30 mL) and added $(NH_4)_2CO_3$ (1.8 g, 20 mmol). The reaction mixture was stirred at r.t. for 6 h. After the completion of the reaction, the reaction was concentrated and extracted with DCM, washed with saturated NaCl solution, the organic layer was dried over anhydrous Na_2SO_4, then was concentrated and used for the next step directly without purification to obtain compound **S11**. The compound **S11** was dissolved in dry DCM (20 mL), then were added CCl_3CN (2 mL), DBU (298 μL). The reaction mixture was stirred at 0ºC for 2 h, after the completion of the reaction, then concentrated and was purified by silica gel chromatography to give the compound **S12**. To a solution of the compound **S12** (1.4 g, 3.2 mmol) and **S9** (1.37 g, 3.2 mmol) in dry DCM (20 mL), then TMSOTf (117 μL, 0.6 mmol) was added at 0ºC, the reaction mixture was stirred at r.t. for 12 h and was quenched with TEA, extracted with DCM and washed with saturated NaCl solution, the organic layer was dried over anhydrous Na_2SO_4, then concentrated and was purified by silica gel chromatography to give the compound **S13**[1] (1.6 g, 72%). ^{1}H NMR (400 MHz, Chloroform-d) δ 7.44 – 7.28 (m, 5H), 6.40 (s, 1H), 6.11 (s, 1H), 5.78 (d, *J* = 8.2 Hz, 1H), 5.30 (dd, *J* = 10.1, 3.5 Hz, 1H), 5.24 (dd, *J* = 3.5, 1.8 Hz, 1H), 5.10 (d, *J* = 2.1 Hz, 2H), 5.06 (d, *J* = 10.0 Hz, 1H), 4.83 (d, *J* = 1.8 Hz, 1H), 4.24 (m, 1H), 3.91 (m, 1H), 3.83 – 3.73 (m, 1H), 3.69 – 3.58 (m, 3H), 3.56 – 3.52 (m, 2H), 3.45 (m, 1H), 2.30 (m, 2H), 2.27 (m, 1H), 2.13 (s, 3H), 2.05 (s, 3H), 1.99 (d, *J* = 3.3 Hz, 3H), 1.87 (m, 1H), 1.45 (s, 9H), 1.22 (d, *J* = 6.3 Hz, 3H). ^{13}C NMR (100 MHz, Chloroform-d) δ 172.25, 171.19, 170.40, 170.30, 169.99, 163.38, 156.28, 136.39, 128.49, 128.11, 128.09, 97.55, 82.20, 71.08, 70.03, 70.00, 69.10, 66.98, 66.89, 66.49, 54.23, 39.34, 32.35, 28.54, 27.98, 20.90, 20.79, 20.70, 17.43. MS-ESI (m/z): calculated for 719.3, observed,719.03, $[M+Na]^+$

Compound 8: Compound **S13** (800 mg, 1.15 mmol) was dissolved in MeOH (20 mL) and added Pd/C (120 mg). The reaction mixture was stirred at r.t under H_2 atmosphere for 12 h, after the completion of the reaction, the product mixture was filtered through Celite, washed with MeOH, and then the organic was concentrated to afford compound **8** (600 mg, 93%). ^{1}H NMR (400 MHz, Methanol-*d₄*) δ 5.28 – 5.19 (m, 2H), 5.02 (t, *J* = 9.7 Hz, 1H), 4.85 (m, 1H), 3.96 (m, 1H), 3.84 (m, 1H), 3.69 (m, 3H), 3.59 (m, 3H), 3.40 (m, 2H), 3.37 (s, 1H), 2.39 (m, 2H), 2.15 (s, 3H), 2.10 (m, 1H), 2.07 (s, 3H), 1.98 (s, 3H), 1.96 (m, 1H), 1.52 (s, 9H), 1.20 (d, *J* = 6.3 Hz, 3H). ^{13}C NMR (100 MHz, Methanol-d_4) δ 174.98, 173.47, 170.36, 170.34, 170.30, 97.51, 82.25, 70.84, 69.66, 69.37, 69.21, 66.86, 66.24, 53.46, 39.04, 31.43, 26.86, 20.68, 19.29, 19.28, 19.23, 16.38. MS-ESI (m/z): calculated for 585.26, observed, 585.15, $[M+H]^+$

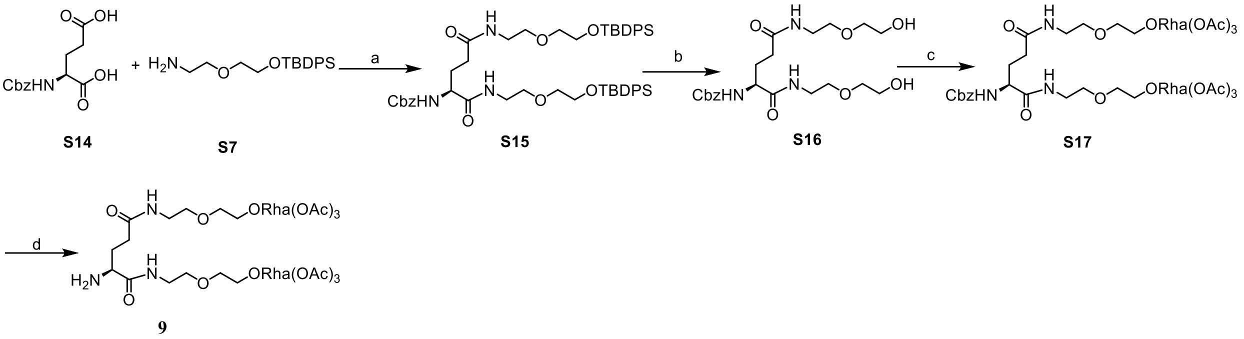

Scheme S4: Reagents and conditions: (a). EDC·HCl, HOBt, TEA, DCM, r.t, 12 h, 88%; (b) THF, TBAF, AcOH, r.t, 12 h, 88%; (c) Rha(OAc)$_3$, trifluoride etherate, DCM, 0ºC, 12 h, 77%; (d) MeOH, Pd/C H_2, r.t, 12 h, 90%;

Compound S15: To a solution of the compound **S14** (11 g, 36 mmol) in DCM (70 mL) and added compound **S7** (24 g, 72 mmol), HOBt (5.9 g, 43 mmol), TEA (10 mL, 72 mmol) and EDC·HCl (8.2 g, 43 mmol). The reaction mixture was stirred at r.t. for 12 h, after the completion of the reaction, the reaction was concentrated and extracted with DCM, washed with saturated NaCl solution, the organic layer was dried over anhydrous Na_2SO_4, then concentrated and was purified by silica gel chromatography to give the compound **S15** (30 g, 88%). ^{1}H NMR (400 MHz, Chloroform-d) δ 7.66 (m, 8H), 7.45 – 7.27 (m, 17H), 6.94 (s, 1H), 6.23 (s, 1H), 5.97 (s, 1H), 5.05 (s, 2H), 4.12 (d, *J* = 6.8 Hz, 1H), 3.79 (m, 4H), 3.53 (m, 8H), 3.38 (m, 4H), 2.25 – 2.09 (m, 2H), 1.97 (m, 2H), 1.04 (s, 18H). MS-ESI (m/z): calculated for 954.45; observed 954.16, [M+Na]$^+$

Compound S16 To a solution of the compound **S15** (21 g, 22 mmol) in THF (50 mL), then added TBAF (66 mmol, 66 mL), AcOH (88 mmol, 8.9 g), and the reaction mixture was stirred at r.t for 12 h. After the completion of the reaction, the reaction was concentrated and was purified by silica gel chromatography to give the compound **S16**[2] (9 g, 88%).

^{1}H NMR (400 MHz, Methanol-*d$_4$*) δ 7.42 – 7.27 (m, 5H), 5.10 (d, *J* = 3.2 Hz, 2H), 4.12 (dd, *J* = 8.7, 5.4 Hz, 1H), 3.72 – 3.64 (m, 4H), 3.55 (m, 8H), 3.41 – 3.36 (m, 4H), 2.31 (m, 2H), 2.14 – 2.03 (m, 1H), 1.93 (m, 1H). MS-ESI (m/z): calculated for 478.22, observed, 478.38, [M+Na]$^+$

Compound S17: To a solution of the compound **S16** (6 g, 13 mmol) in DCM (70 mL) and added compound Rha(OAc)$_3$ (10.7 g, 32 mmol), then boron trifluoride etherate (15 mL) was added at 0ºC, the reaction mixture was stirred at r.t. for 12 h[3]. After the completion of the reaction, the reaction was diluted with DCM, washed with saturated $NaHCO_3$ and NaCl solution, the organic layer was dried over anhydrous Na_2SO_4, then concentrated and was purified by silica gel chromatography to give the compound **S17** (10 g, 77%). ^{1}H NMR (400 MHz, Chloroform-d) δ 7.36 – 7.29 (m, 5H), 7.17 (s, 1H), 6.58 (s, 1H), 6.16 (s, 1H), 5.30 (d, *J* = 3.5 Hz, 1H), 5.27 (d, *J* = 3.4 Hz, 1H), 5.27 – 5.21 (m, 2H), 5.11 – 5.04 (m, 4H), 4.82 (m, 2H), 4.22 (m, 1H), 3.90 (m, 2H), 3.77 (m, 2H), 3.64 (m, 6H), 3.55 (m, 5H), 3.50 – 3.38 (m, 4H), 2.38 (m, 2H), 2.14 (m, 8H), 2.05 (m, 6H), 1.99 (s, 6H), 1.23 (d, *J* = 6.2 Hz, 6H). ^{13}C NMR (100 MHz, Chloroform-d) δ 172.84, 171.56, 170.39, 170.35, 170.30, 170.20, 170.04, 170.00, 156.25, 136.42, 128.49, 128.09, 128.01, 97.51, 97.47, 71.02, 69.93, 69.11, 66.98, 66.90, 66.80, 66.44, 54.35, 39.35, 32.36, 29.16, 20.95, 20.92, 20.82, 20.80, 20.75, 17.42. MS-ESI (m/z): calculated for, 1022.4, observed, 1022.08, [M+Na]$^+$

Compound 9: Compound **S17** (1.8 g, 1.8 mmol) was dissolved in MeOH (20 mL) and added Pd/C (200 mg), the reaction mixture was stirred at r.t under H_2 atmosphere for 12 h. After the completion of the reaction, the product mixture was filtered through Celite, washed with MeOH, and then the solvent was concentrated to afford compound **9** (1.4 g, 90%). ^{1}H NMR (400 MHz, Chloroform-d) δ 7.72 (t, *J* = 5.7 Hz, 1H), 6.71 (t, *J* = 5.6 Hz, 1H), 5.31 – 5.28 (m, 1H), 5.26 (d, *J* = 3.8 Hz, 2H), 5.23 (dd, *J* = 3.5, 1.8 Hz, 1H), 5.06 (m, 2H), 4.80 (dd, *J* = 10.6, 1.6 Hz, 2H), 3.90 (dd, *J* = 9.8, 6.3 Hz, 2H), 3.82 – 3.75 (m, 2H), 3.68 – 3.60 (m, 6H), 3.59 – 3.54 (m, 4H), 3.47 – 3.42 (m, 5H), 2.35 (t, *J* = 7.1 Hz, 2H), 2.18 (s, 2H), 2.15 (s, 3H), 2.14 (s, 3H), 2.06 – 2.04 (m, 6H), 1.99 (s, 3H), 1.98 (s, 3H), 1.24 (d, *J* = 6.2 Hz, 6H). MS-ESI (m/z): calculated for 866.38; observed, 866.02, [M+H]$^+$

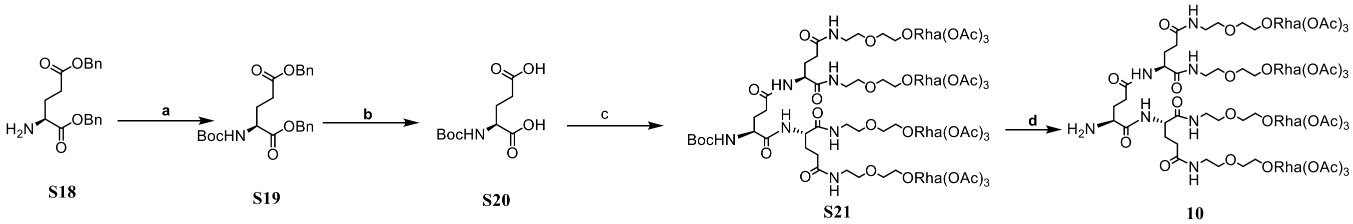

Scheme S5: Reagents and conditions: (a) MeOH, Boc$_2$O, TEA, r.t, 2 h,94%; (b) MeOH, Pd/C H_2, r.t, 12 h, 91%; (c) compound **8**, EDC·HCl, HOBt, TEA, DCM, r.t, 12 h, 78%; (d) 25% TFA/DCM, 0ºC, 2 h, 95%.

Compound S19: To a solution of the compound **S18** (3.27 g, 10 mmol) in MeOH (30 mL) and added Boc$_2$O (2.5 g, 12 mmol), TEA (2.7 mL, 20 mmol). The reaction mixture was stirred at r.t. for 2 h, after the completion of the reaction, then concentrated and was purified by silica gel chromatography to give the compound **S19** (4 g, 94%). ^{1}H NMR (400 MHz, Chloroform-d) δ 7.37 (m, 10H), 5.18 (d, *J* = 3.0 Hz, 2H), 5.12 (s, 2H), 4.41 (m, 1H), 2.45 (m, 2H), 2.21 (m, 1H), 2.00 (m, 1H), 1.45 (s, 9H). MS-ESI (m/z): calculated for 450.19; observed, 450.00, [M+Na]$^+$

Compound S20: Compound **S19** (2 g, 4.6 mmol) was dissolved in MeOH (20 mL) and added Pd/C (400 mg). The reaction mixture was stirred at room temperature under H_2 atmosphere for 12 h, after the completion of the reaction, the product mixture was filtered through Celite, washed with MeOH, and then the solvent was concentrated to afford compound **S20** (1 g, 91%). ^{1}H NMR (400 MHz, Methanol-*d4*) δ 4.15 (m, 1H), 2.42 (m, 2H), 2.20 – 2.07 (m, 1H), 1.98 – 1.85 (m, 1H), 1.46 (s, 9H). MS-ESI (m/z): calculated for, 270.10; observed, 269.94, [M+Na]$^+$

Compound S21: To a solution of the compound **S20** (123 mg, 0.5 mmol) in DCM (20 mL) and added compound **8** (995 mg, 1.15 mmol), HOBt (164 mg, 1.2 mmol), TEA (0.5 mL, 2 mmol) and EDC·HCl (229 mg, 1.2 mmol). The reaction mixture was stirred at r.t for 12 h, after the completion of the reaction, the reaction was diluted with DCM and washed with saturated NaCl solution, the organic layer was dried over anhydrous Na_2SO_4, then concentrated and was purified by silica gel chromatography to give the compound **S21** (760 mg, 78%). ^{1}H NMR (600 MHz, Chloroform-d) δ 8.81 (m, 1H), 8.56 (m, 1H), 7.31 (m, 1H), 7.21 (m, 1H), 6.79 (m, 1H), 6.55 (m, 1H), 5.28 (m, 4H), 5.24 (m, 4H), 5.09 – 5.05 (m, 4H), 4.84 – 4.80 (m, 4H), 4.44 (m, 1H), 4.38 (m, 1H), 3.91 (m, 4H), 3.87 – 3.83 (m, 1H), 3.81 – 3.77 (m, 4H), 3.66 (m, 14H), 3.57 (m, 8H), 3.50 – 3.42 (m, 6H), 2.43 (m, 1H), 2.37 (m, 6H), 2.24 (m, 1H), 2.15 (s, 12H), 2.12 – 2.09 (m, 2H), 2.06 (s, 12H), 2.01 (s, 12H), 1.96 – 1.91 (m, 1H), 1.79 (m, 1H), 1.42 (s, 9H), 1.23 (d, *J* = 6.3 Hz, 12H). ^{13}C NMR (150 MHz, Chloroform-d) δ 172.29, 171.86,171.33, 171.00, 169.32, 169.27, 169.23, 169.10, 169.02, 168.96, 96.54, 96.47, 96.44, 70.08, 70.02, 69.03, 68.95, 68.91, 68.84, 68.79, 68.71, 68.65, 68.13, 68.10, 68.09, 65.99, 65.90, 65.85, 65.43, 65.40, 65.37, 52.42, 38.67, 38.58, 38.30, 38.19, 31.51, 31.44, 30.78, 27.53, 27.35, 27.14, 19.93, 19.81, 19.74, 16.43, 16.41. MS-MALDI-TOF (m/z): calculated for, 1964.81; observed, 1964.90 [M+Na]$^+$

Compound 10: To a solution of the compound **S21** (700 mg, 0.36 mmol) in DCM (10 mL), then 50% TFA/DCM (10 mL) was added dropwise at 0ºC, then the reaction mixture was stirred at 0ºC for 2 h. After the completion of the reaction, the reaction was diluted with toluene (5 mL) and concentrated in vacuo. The residual TFA was removed from the crude residue by azeotroping with toluene (3×5 mL) and was concentrated to give the compound **10**, which was used for the next step directly.

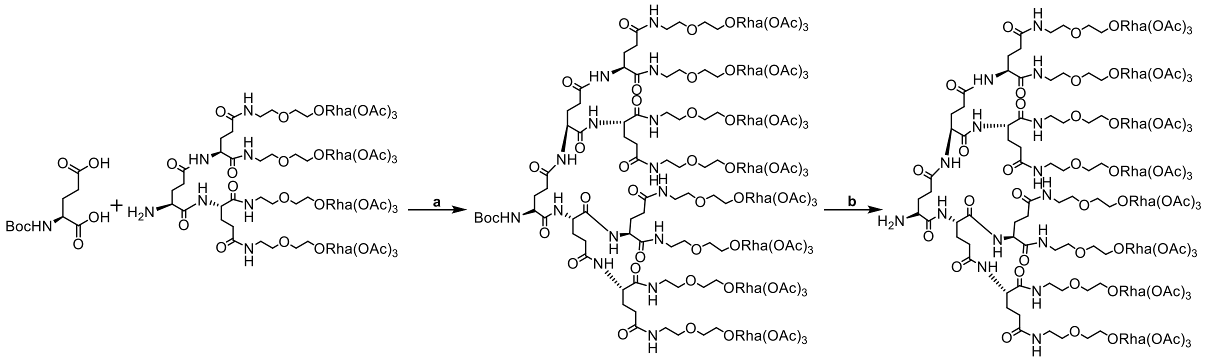

S22 S23 S24 11

Scheme S6: Reagents and conditions: (a) EDC·HCl, HOBt, TEA, DCM, r.t, 12 h, 85%; (b) 25% TFA/DCM, 0ºC, 2 h, 93%.

Compound S24: To a solution of the compound **S22** (131 mg, 0.53 mmol) in DCM (30 mL) and compound **S23** (2.1 g, 1.2 mmol), HOBt (180 mg, 1.32 mmol), TEA (0.3 mL, 2 mmol) and EDC·HCl (253 g, 1.32 mmol) were added at 0°C, the reaction mixture was stirred at r.t. for 12 h. After the completion of the reaction, the reaction was concentrated and extracted with DCM, washed with saturated NaCl solution, the organic layer was dried over anhydrous Na_2SO_4, then concentrated and was purified by silica gel chromatography to give the compound **S24** (1.7 g, 85%). ^{1}H NMR (400 MHz, Methanol-*d4*) δ 5.27 – 5.19 (m, 16H), 5.02 (m, 8H), 4.88 – 4.85 (m, 8H), 4.48 – 4.33 (m, 4H), 4.25 – 4.11 (m, 3H), 3.96 (m, 8H), 3.83 (m, 8H), 3.70 (m, 25H), 3.60 (m, 18H), 3.51 – 3.38 (m, 14H), 2.48 – 2.28 (m, 14H), 2.15 (s, 24H), 2.14 – 2.09 (m, 5H), 2.07 (s, 24H), 2.05 – 2.00 (m, 3H), 1.98 (s, 24H), 1.96 – 1.83 (m, 6H), 1.46 (s, 9H), 1.21 (d, *J* = 6.2, 24H). ^{13}C NMR (100 MHz, Methanol-*d4*) δ 173.60, 173.54, 173.49, 173.38, 173.04, 172.79, 170.33, 170.31, 170.30, 170.26, 170.22, 170.20, 97.48, 97.45, 70.81, 69.72, 69.64, 69.33, 69.20, 66.89, 66.83, 66.22, 39.20, 39.01, 31.78, 27.74, 27.54, 19.40, 19.36, 19.32, 16.49,16.47. MS-MALDI-TOF (m/z): calculated for 3917.62; observed, 3917.45, [M+Na]$^+$

Compound 11: To a solution of the compound **S24** (1.6 g, 0.41 mmol) in DCM (10 mL), then 50% TFA/DCM (10 mL) was added dropwise at 0ºC, then the reaction mixture was stirred at 0ºC for 2 h. After the completion of the reaction, the reaction was diluted with toluene (5 mL), and concentrated in vacuo. The residual TFA was removed from the crude residue by azeotroping with toluene (3×5 mL) and was concentrated to give the compound **11** (1.4 g, 93%).

Chemical Synthesis Procedures for intermediate compound 1-5

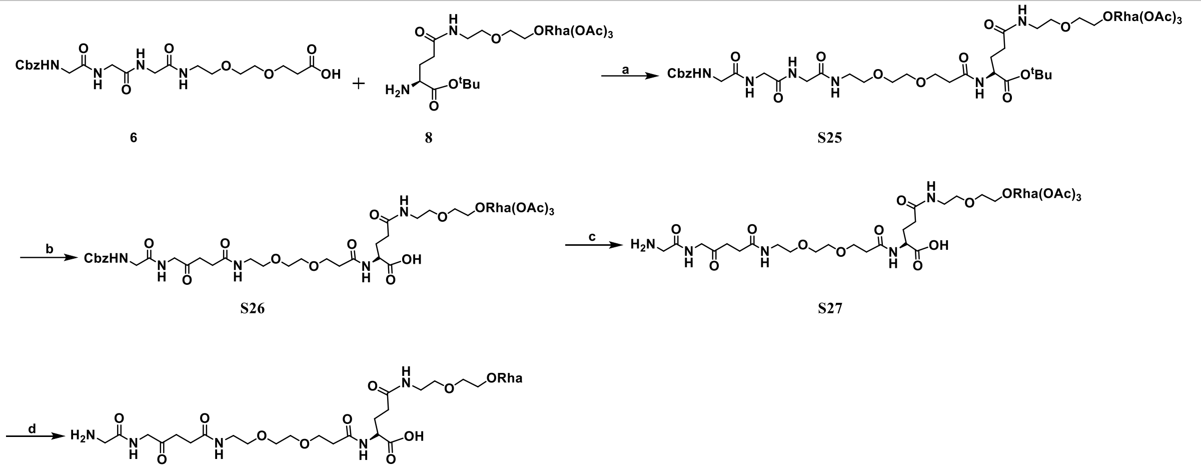

1

(a) EDC·HCl, HOBt, TEA, DMF, r.t, 12 h, 48%; (b) 25% TFA/DCM, 0ºC, 2 h, 95%; (c) MeOH, Pd/C H_2, r.t, 12 h, 86%; (d) MeOH, MeONa, r.t, 12 h, 91%.

Compound S25: To a solution of the compound **6** (482 mg, 1 mmol) in DMF (15 mL) and added compound **8** (562 mg, 1 mmol), HOBt (164 mg, 1.2 mmol), TEA (0.3 mL, 2 mmol) and EDC·HCl (229 mg, 1.2 mmol). The reaction mixture was stirred at r.t. for 12 h, after the completion of the reaction, the reaction was concentrated and extracted with DCM, washed with saturated NaCl solution, the organic layer was dried over anhydrous Na_2SO_4, then concentrated and was purified by silica gel chromatography to give the compound **19** (480 mg, 48%). ^1H NMR (600 MHz, Chloroform-d) δ 7.59 (m, 1H), 7.52 (m, 1H), 7.34 (m, 5H), 7.31 (m, 2H), 6.89 (m, 1H), 6.17 (m, 1H), 5.26 (m, 1H), 5.22 (m, 1H), 5.10 (d, *J* = 7.5 Hz, 2H), 5.07 (d, *J* = 9.9 Hz, 1H), 4.83 – 4.79 (m, 1H), 4.42 (m, 1H), 3.99 (m, 2H), 3.92 (m, 5H), 3.78 – 3.70 (m, 3H), 3.67 – 3.57 (m, 7H), 3.53 (t, *J* = 4.8 Hz, 4H), 3.41 (m, 4H), 2.50 (m, 2H), 2.28 (m, 2H), 2.15 (s, 3H), 2.11 (m, 1H), 2.05 (s, 3H), 2.00 (s, 3H), 1.95 (m, 1H), 1.44 (s, 9H), 1.22 (d, *J* = 6.3 Hz, 3H). ^{13}C NMR (150 MHz, Chloroform-d) δ 172.77, 171.60, 171.36, 170.45, 169.95, 169.55, 169.25, 136.25, 128.53, 128.20, 128.08, 97.49, 82.26, 70.96, 70.13, 70.08, 69.98, 69.91, 69.49, 69.20, 67.13, 66.94, 66.92, 66.51, 52.60, 44.51, 43.07, 42.97, 39.38, 39.30, 36.73, 32.34, 28.16, 27.99, 20.92, 20.78, 20.74, 17.42. MS-ESI (m/z): calculated for 1049.45; observed,1049.27, $[M+Na]^+$

Compound S26: To a solution of the compound **S25** (300 mg, 0.29 mmol) in DCM (6 mL), then 50% TFA/DCM (6 mL) was added dropwise at 0ºC, and the reaction mixture was stirred at 0ºC for 2 h. After the completion of the reaction, the reaction was diluted with toluene three times, and concentrated in vacuo. The residual TFA was removed from the crude residue by azeotroping with toluene (3×5 mL) and was concentrated to give the compound **S26** (270 mg, 95%). ^1H NMR (600 MHz, Chloroform-d) δ 8.15 (brs, 1H), 8.04 (brs, 1H), 7.79 (brs, 1H), 7.30 (m, 5H), 7.17 (brs, 1H), 6.64 (brs, 1H), 5.27 – 5.20 (m, 2H), 5.06 (m, 2H), 4.80 – 4.77 (m, 1H), 4.40 (brs, 1H), 3.93 – 3.84 (m, 7H), 3.76 – 3.68 (m, 4H), 3.61 (m, 6H), 3.51 (m, 8H), 3.36 (m, 2H), 2.50 (s, 2H), 2.32 (s, 2H), 2.13 (s, 3H), 2.11 (m, 1H), 2.04 (s, 3H), 1.98 (s, 3H), 1.94 (m, 1H), 1.21 (d, *J* = 6.2 Hz, 3H). ^{13}C NMR (150 MHz, Chloroform-d) δ 170.49, 170.05, 136.20, 128.51, 128.17, 127.99, 97.45, 70.92, 69.85, 69.82, 69.65, 69.26, 67.17, 66.90, 66.47, 50.69, 20.89, 20.77, 20.72, 17.38. MS-ESI (m/z): calculated for 993.39, observed, 993.11, $[M+Na]^+$

Compound S27: Compound **S26** (350 mg, 0.36 mmol) was dissolved in MeOH (10 mL) and added Pd/C (30 mg). The reaction mixture was stirred at r.t. under H_2 atmosphere for 12 h. After the completion of the reaction, the product mixture was filtered through Celite, washed with MeOH, and then solvent was concentrated to afford compound **S27** (260 mg, 86%). ^1H NMR (600 MHz, Methanol-*d4*) δ 5.24 – 5.19 (m, 2H), 5.00 (t, *J* = 9.9 Hz, 2H), 4.82 (d, *J* = 1.7 Hz, 2H), 4.23 (m, 1H), 3.98 (s, 2H), 3.96 – 3.91 (m, 2H), 3.90 (s, 2H), 3.83 – 3.78 (m, 3H), 3.76 (m, 3H), 3.67 (m, 4H), 3.63 (m, 5H), 3.58 (m, 5H), 3.41 – 3.36 (m, 5H), 2.54 (m, 2H), 2.29 (m, 2H), 2.13 (s, 3H), 2.05 (s, 3H), 1.96 (s, 3H), 1.18 (d, *J* = 6.3 Hz, 3H). ^{13}C NMR (150 MHz, Methanol-*d4*) δ 174.34, 172.36, 170.58, 170.37, 167.77, 97.48, 70.80, 69.90, 69.69, 69.65, 69.63, 69.37, 69.28, 69.08, 66.86, 66.79, 66.23, 42.56, 42.17, 40.56, 39.04, 38.96, 36.12, 32.15, 28.47, 19.34, 19.32, 19.27, 16.41. MS-ESI (m/z): calculated for:859.35; observed, 859.34, $[M+Na]^+$

Compound 1: Compound **S27** (200 mg, 0.23 mmol) was dissolved in MeOH (10 mL) and added MeONa (10 µL). The reaction mixture was stirred at r.t for 12 h, after the completion of the reaction, the product mixture was neutralized with acid resin and filtered, washed with MeOH, and then the solvent was concentrated to afford compound **1** (150 mg, 91%).

^1H NMR (400 MHz, Methanol-*d4*) δ 4.73 (s, 1H), 4.44 (m, 1H), 3.99 (s, 2H), 3.90 (s, 2H), 3.84 – 3.74 (m, 6H), 3.66 – 3.55 (m, 13H), 3.41 – 3.34 (m, 5H), 2.55 (m, 2H), 2.33 (t, *J* = 7.5 Hz, 2H), 2.23 – 2.15 (m, 1H), 2.03 – 1.90 (m, 1H), 1.26 (d, *J* = 6.2 Hz, 3H). ^{13}C NMR (100 MHz, Methanol-*d4*) δ 173.61, 172.79, 170.46, 170.31, 167.02, 100.33, 72.62, 70.97, 70.81, 69.87, 69.76, 69.19, 69.07, 68.41, 66.78, 66.38, 51.80, 48.06, 47.92, 47.77, 47.63, 47.49, 47.35, 47.21, 42.36, 40.35, 39.06, 38.95, 35.93, 31.81, 27.41, 16.66. MS-ESI (m/z): calculated for:733.33; observed, 733.36, $[M+Na]^+$

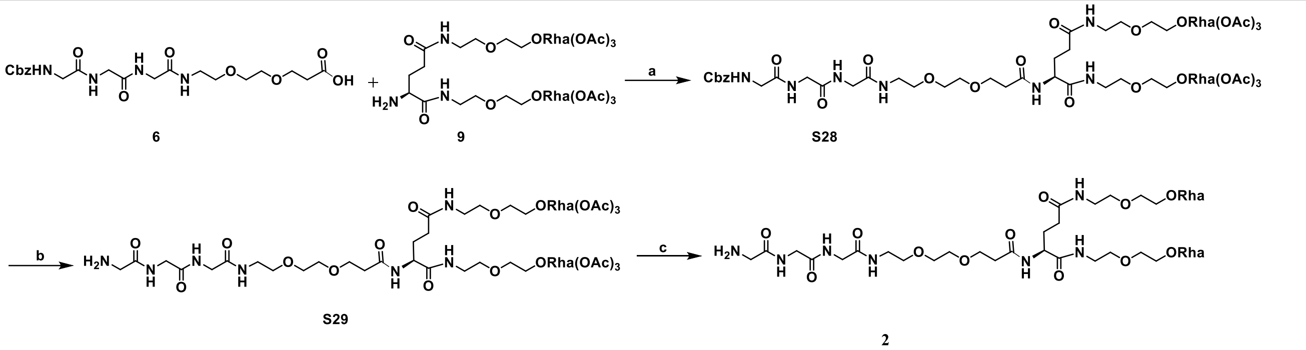

(a) EDC·HCl, HOBt, TEA, DMF, r.t, 12 h, 68%; (b) MeOH, Pd/C, H_2, r.t, 12 h, 92%; (c) MeOH, MeONa, r.t, 12 h, 80%.

Compound S28: To a solution of the compound **9** (190 mg, 0.2 mmol) in DMF (50 mL) and added compound **6** (106 mg, 0.22 mmol), HOBt (33 mg, 0.24 mmol), TEA (55 μL, 0.4 mmol) and EDC·HCl (46 mg, 0.24 mmol) at 0°C. The reaction mixture was warmed to r.t. and stirred for 12 h. After the completion of the reaction, the reaction was concentrated and diluted with DCM, washed with saturated NaCl solution, the organic layer was dried over anhydrous Na_2SO_4. The concentrated residue was purified by silica gel chromatography to give the compound **S28** (180 mg, 68%). ^{1}H NMR (400 MHz, Chloroform-d) δ 7.61 (m, 1H), 7.59 – 7.53 (m, 1H), 7.43 (m, 3H), 7.37 – 7.31 (m, 5H), 6.85 (m, 1H), 6.24 (m, 1H), 5.31 (s, 2H), 5.27 (m, 1H), 5.25 – 5.22 (m, 3H), 5.14 – 5.03 (m, 4H), 4.81 (t, *J* = 1.5 Hz, 2H), 4.48 (m, 1H), 4.03 – 3.85 (m, 8H), 3.81 – 3.71 (m, 4H), 3.64 – 3.53 (m, 14H), 3.48 – 3.35 (m, 6H), 2.52 – 2.44 (m, 2H), 2.36 (m, 4H), 2.15 (s, 6H), 2.06 (s, 6H), 2.00 (s, 3H), 1.99 (s, 3H), 1.23 (d, *J* = 6.2 Hz, 6H); ^{13}C NMR (100 MHz, Chloroform-d) δ 172.96, 171.83, 171.68, 170.50, 170.37,170.30, 170.00, 169.99, 169.63, 169.30, 156.96, 128.53, 128.19, 128.10, 97.45, 97.43, 70.95, 70.04, 69.95, 69.88, 69.86, 69.82, 69.77, 69.59, 69.21, 69.18, 67.08, 66.90, 66.88, 66.82, 66.47, 66.45, 53.44, 52.76, 44.48, 43.10, 42.98, 39.35, 39.31, 39.28, 36.75, 32.36, 28.73, 20.94, 20.81, 20.77, 17.42. MS-ESI (m/z): calculated for 1352.55, observed 1352.52 $[M+Na]^+$

Compound S29: Compound **S28** (150 mg, 0.11 mmol) was dissolved in MeOH (5 mL) and added Pd/C (10 mg), the reaction mixture was stirred at r.t. under H_2 atmosphere for 12 h. After the completion of the reaction, the product mixture was filtered through Celite, washed with MeOH, and then the solvent was concentrated to afford compound **S29** (120 mg, 92%). ^{1}H NMR (400 MHz, Methanol-*d₄*) δ 8.22 (brs, 4H), 5.24 – 5.18 (m, 4H), 5.00 (m, 2H), 4.83 (m, 4H), 4.35 (m, 1H), 3.99 (s, 2H), 3.93 (m, 3H), 3.90 (s, 1H), 3.84 – 3.74 (m, 6H), 3.68 (m, 6H), 3.63 (s, 4H), 3.57 (m, 6H), 3.39 (m, 6H), 2.54 (m, 2H), 2.31 (m, 2H), 2.13 (s, 6H), 2.05 (s, 6H), 1.96 (s, 6H), 1.18 (d, *J* = 6.2 Hz, 6H). ^{13}C NMR (100 MHz, Methanol-d_4) δ 173.65, 172.69, 172.55, 170.41, 170.39, 170.37, 170.35, 170.24, 167.03, 165.68, 161.95, 97.48, 70.83, 69.87, 69.75, 69.70, 69.66, 69.39, 69.26, 69.22, 69.08, 66.88, 66.85, 66.82, 66.25, 66.24, 52.92, 48.48, 42.37, 42.12, 40.38, 39.03, 39.01, 38.94, 36.09, 31.75, 27.85, 19.35, 19.34, 19.28, 16.42. MS-ESI (m/z): calculated for 1218.51, observed 1218.31 $[M+Na]^+$

Compound 2: Compound **S29** (100 mg, 0.08 mmol) was dissolved in MeOH (3 mL) and added MeONa (4 μL), the reaction mixture was stirred at r.t. for 12 h. After the completion of the reaction, the product mixture was neutralized with acid resin and filtered, washed with MeOH, and then the solvent was concentrated to afford compound **2** (65 mg, 80%).

^{1}H NMR (400 MHz, Methanol-d_4) δ 8.24 – 7.92 (m, 2H), 4.75 (d, *J* = 1.6 Hz, 2H), 4.38 (m, 1H), 4.02 (m, 2H), 3.92 (m, 2H), 3.85 – 3.74 (m, 8H), 3.69 – 3.62 (m, 12H), 3.58 (m, 7H), 3.43 – 3.35 (m, 8H), 2.60 – 2.53 (m, 2H), 2.33 (m, 2H), 2.18 – 2.05 (m, 1H), 2.00 – 1.86 (m, 1H), 1.28 (d, *J* = 6.2 Hz, 6H). ^{13}C NMR (100 MHz, Methanol-d_4) δ 173.69, 172.75, 172.51, 170.33, 170.18, 166.88, 100.36, 100.34, 72.64, 71.01, 70.99, 70.83, 69.89, 69.84, 69.77, 69.20, 69.14, 69.02, 68.45, 66.82, 66.36, 66.33, 52.90, 42.25, 42.09, 40.36, 39.10, 38.99, 36.13, 31.85, 27.97, 16.70, 16.69. MS-ESI (m/z): calculated for 966.46 observed, 966.14, $[M+Na]^+$

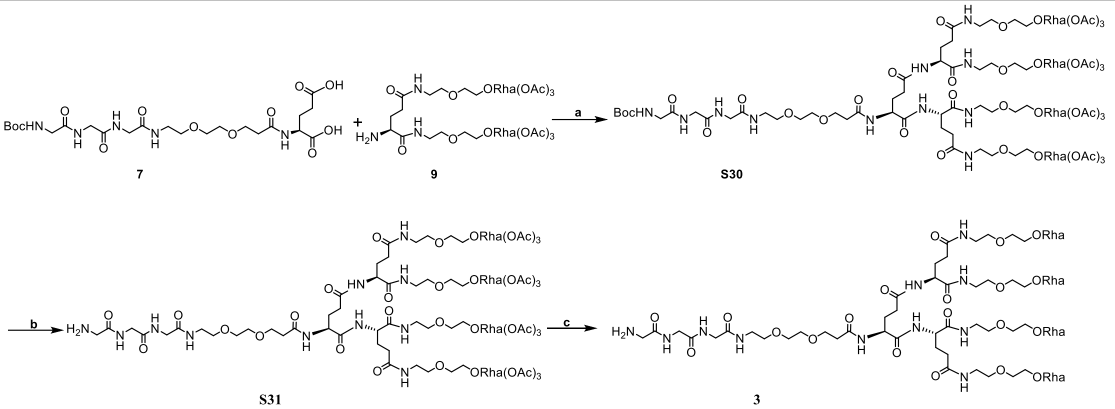

(a) EDC·HCl, HOBt, TEA, DCM, r.t, 12 h, 73%; (b) 25% TFA/DCM, 0ºC, 2 h, 90%; (c) MeOH, MeONa, r.t, 24 h, 90%.

Compound S30: To a solution of the compound **7** (288 mg, 0.5 mmol) in DCM (15 mL) and added compound **9** (1.04 mg, 1.2 mmol), HOBt (164 mg, 1.2 mmol), TEA (0.3 mL, 2 mmol) and EDC·HCl (229 mg, 1.2 mmol). The reaction mixture was stirred at r.t. for 12 h, after the completion of the reaction, the reaction was concentrated and extracted with DCM, washed with saturated NaCl solution, the organic layer was dried over anhydrous Na_2SO_4, then concentrated and was purified by silica gel chromatography to give the compound **S30** (800 mg, 73%). ^{1}H NMR (600 MHz, Chloroform-d) δ 8.99 (m, 1H), 8.59 (m, 1H), 7.74 (m, 1H), 7.66 (m, 1H), 7.56 – 7.48 (m, 2H), 7.39 (m, 1H), 7.13 (s, 1H), 7.02 (m, 1H), 6.62 (m, 1H), 5.86 (m, 1H), 5.29 – 5.21 (m, 8H), 5.06 (m, 4H), 4.83 – 4.77 (m, 4H), 4.50 – 4.36 (m, 2H), 4.19 – 4.12 (m, 1H), 4.05 – 3.93 (m, 4H), 3.92 – 3.84 (m, 6H), 3.78 (m, 4H), 3.72 (m, 2H), 3.69 – 3.52 (m, 27H), 3.51 – 3.43 (m, 4H), 3.43 – 3.30 (m, 7H), 2.51 – 2.33 (m, 6H), 2.30 – 2.21 (m, 2H), 2.15 (d, *J* = 2.2 Hz, 12H), 2.12 – 2.07 (m, 2H), 2.05 (d, *J* = 1.5 Hz, 12H), 1.99 (t, *J* = 2.7 Hz, 12H), 1.95 – 1.91 (m, 1H), 1.83 (m, 1H), 1.43 (d, *J* = 1.9 Hz, 9H), 1.22 (d, *J* = 2.7 Hz, 12H). ^{13}C NMR (150 MHz, Chloroform-d) δ 173.28, 173.08,172.79, 172.49, 170.34, 170.31, 170.27, 170.23, 170.03, 169.98, 169.85, 156.35, 97.54, 97.51, 97.49, 97.46, 71.03, 71.01, 70.19, 69.91, 69.87, 69.86, 69.81, 69.70, 69.66, 69.55, 69.19, 69.16, 69.14, 69.12, 66.99, 66.95, 66.89, 66.71, 66.45, 66.43, 66.39, 53.44, 53.09, 52.48, 43.18, 43.02, 39.65, 39.56, 39.32, 39.17, 36.40, 32.40, 32.34, 31.67, 28.72, 28.36, 28.19, 20.94, 20.82, 20.76, 17.42. MS-MALDI-TOF (m/z): calculated for 2294.98; observed, 2295.01, [M+Na]$^+$

Compound **S31**: To a solution of the compound **S30** (750 mg, 0.33 mmol) in DCM (10 mL), then 50% TFA/DCM (10 mL) was added dropwise at 0ºC, then the reaction mixture was stirred at 0ºC for 2 h. After the completion of the reaction, the reaction was diluted with toluene (5 mL), and concentrated in vacuo. The residual TFA was removed from the crude residue by azeotroping with toluene (3×5 mL) and was concentrated to give the compound **S31** (650 mg, 90%). ^{1}H NMR (400 MHz, Methanol-d_4) δ 5.28 – 5.18 (m, 8H), 5.02 (t, *J* = 9.8 Hz, 4H), 4.87 – 4.84 (m, 4H), 4.37 (m, 2H), 4.18 (m, 1H), 4.02 (s, 2H), 3.98 – 3.90 (m, 6H), 3.86 – 3.74 (m, 8H), 3.73 – 3.67 (m, 12H), 3.61 (m, 14H), 3.47 (m, 2H), 3.40 (m, 8H), 2.54 (m, 2H), 2.36 (m, 6H), 2.15 (s, 12H), 2.12 (m, 2H), 2.07 (s, 12H), 1.98 (m, 12H), 1.94 (m, 2H), 1.20 (d, *J* = 6.2 Hz, 12H). ^{13}C NMR (100 MHz, Methanol-d_4) δ 173.65, 173.55, 173.45, 173.35, 173.23, 172.54, 172.41, 170.35, 170.33, 170.29, 97.47, 70.79, 69.91, 69.76, 69.71, 69.68, 69.65, 69.63, 69.35, 69.26, 69.17, 69.02, 66.89, 66.87, 66.83, 66.70, 66.24, 53.00, 52.64, 42.22, 42.05, 40.26, 39.18, 39.02, 38.99, 35.84, 31.74, 27.79, 27.75, 19.36, 19.30, 16.45, 16.44. MS-MALDI-TOF (m/z): calculated for 2194.92; observed, 2194.89, [M+Na]+

Compound **3**: Compound **S31** (600 mg, 0.27 mmol) was dissolved in MeOH (10 mL) and was added MeONa (10 μL). The reaction mixture was stirred at room temperature 24 h, after the completion of the reaction, the product mixture was neutralized with acid resin and filtered, washed with MeOH, and then the solvent was concentrated to afford compound **3** (450 mg, 90%). ^{1}H NMR (400 MHz, D_2O) δ 4.69 (s, 4H), 4.22 – 4.09 (m, 3H), 3.94 (s, 2H), 3.85 – 3.80 (m, 6H), 3.79 (s, 2H), 3.72 (M, 2H), 3.71 – 3.63 (m, 8H), 3.60 (M, 7H), 3.58 – 3.47 (m, 22H), 3.38 – 3.25 (m, 14H), 3.23 (m, 3H), 2.48 (m, 2H), 2.33 – 2.20 (m, 6H), 2.00 (m, 1H), 1.93 – 1.80 (m, 3H), 1.16 (d, *J* = 6.2 Hz, 12H). ^{13}C NMR (100 MHz, D_2O) δ 174.85, 174.08, 173.58, 173.35, 174.22, 171.67, 171.23, 167.81, 163.00, 162.76, 99.97, 72.04, 70.26, 70.09, 69.53, 69.38, 68.81, 68.77, 68.73, 68.70, 68.60, 66.57, 66.48, 53.37, 53.28, 48.88, 42.38, 42.33, 40.44, 39.06, 39.03, 39.00, 38.93, 35.60, 31.88, 31.85, 31.27, 27.28, 26.98, 20.38, 16.65. MS-MALDI-TOF (m/z): calculated for 1690.80, observed, 1690.89 [M+Na]$^+$

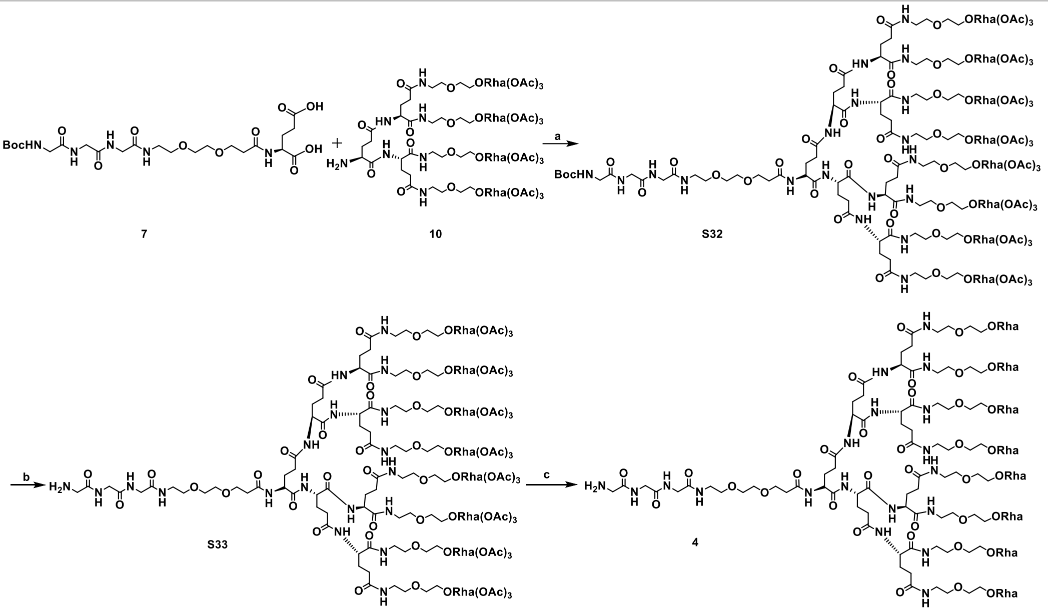

(a) EDC·HCl, HOBt, TEA, DMF, r.t, 12 h, 67%; (b) 25% TFA/DCM, 0ºC, 2 h, 86%; (c) MeOH, MeONa, r.t, 24 h, 83%.

Compound S32: To a solution of the compound **7** (719 mg, 0.39 mmol) in DCM (20 mL) and added compound **10** (100 mg, 0.17 mmol), HOBt (74 mg, 0.5 mmol), TEA (0.2 mL, 0.8 mmol) and EDC·HCl (100 mg, 0.5 mmol), the reaction mixture was stirred for 12 h. After the completion of the reaction, the reaction was diluted with DCM, washed with saturated NaCl solution, the organic layer was dried over anhydrous Na_2SO_4. Then the residue concentrated and was purified by silica gel chromatography to give the compound **S32** (482 mg, 67%). ^{1}H NMR (400 MHz, Chloroform-d) δ 9.12 – 8.93 (m, 2H), 8.92 – 8.75 (m, 2H), 8.60 (m, 1H), 8.30 (m, 1H), 8.04 – 7.90 (m, 2H), 7.61 (m, 1H), 7.54 (m, 1H), 7.41 (m, 2H), 7.01 (m, 2H), 6.71 (m, 1H), 6.62 (m, 1H), 5.99 (m, 1H), 5.31 (m, 20H), 5.06 (m, 8H), 4.87 – 4.74 (m, 8H), 4.48 (m, 2H), 4.41 (m, 2H), 4.02 (m, 5H), 3.96 – 3.73 (m, 21H), 3.73 – 3.67 (m, 6H), 3.67 – 3.49 (m, 45H), 3.43 (m, 12H), 2.91 (s, 6H), 2.47 (s, 2H), 2.43 – 2.30 (m, 8H), 2.30 – 2.18 (m, 5H), 2.14 (s, 24H), 2.10 (m, 3H), 2.06 (s, 24H), 1.99 (s, 24H), 1.89 (m, 4H), 1.43 (s, 9H), 1.22 (d, *J* = 6.3 Hz, 24H). ^{13}C NMR (100 MHz, Chloroform-d) δ 173.83, 173.43, 172.93, 172.79, 172.70, 172.45, 170.29, 170.33, 170.29, 170.27, 170.24, 170.18, 170.15, 170.09, 170.05, 170.03, 170.01, 169.99, 169.96, 97.54, 97.52, 97.46, 79.71, 71.11, 71.06, 71.03, 71.00, 70.98, 70.08, 69.93, 69.91, 69.89, 69.86, 69.84, 69.81, 69.71, 69.66, 69.55, 69.16, 69.10, 67.11, 67.04, 66.95, 66.90, 66.44, 66.42, 66.38, 66.36, 66.32, 53.44, 53.08, 52.97, 52.75, 39.72, 39.60, 39.52, 39.37, 39.29, 39.20, 39.06, 32.28, 28.37, 28.31, 28.09, 20.92, 20.82, 20.80, 20.73, 17.41. MS-MALDI-TOF (m/z): calculated for 4247.78; observed, 4247.74, $[M+Na]^+$

Compound S33: To a solution of the compound **S32** (450 mg, 0.1 mmol) in DCM (10 mL), then 50% TFA/DCM (10 mL) was added dropwise at 0ºC, then the reaction mixture was stirred at 0ºC for 2 h. After the completion of the reaction, the reaction was diluted with toluene (5 mL), and concentrated in vacuo. The residual TFA was removed from the crude residue by azeotroping with toluene (3×5 mL) and was concentrated to give the compound **S33** (380 mg, 86%). ^{1}H NMR (400 MHz, Methanol-*d*$_4$) δ 7.21 (m, 4H), 7.16 – 7.09 (m, 6H), 5.26 – 5.16 (m, 16H), 5.00 (t, *J* = 9.8 Hz, 8H), 4.84 (d, *J* = 3.3 Hz, 8H), 4.35 (dd, *J* = 9.5, 5.0 Hz, 3H), 4.19 (dd, *J* = 11.2, 6.3 Hz, 3H), 4.02 – 3.89 (m, 14H), 3.81 (m, 11H), 3.70 – 3.65 (m, 24H), 3.62 – 3.53 (m, 25H), 3.47 (m, 4H), 3.39 (m, 13H), 2.40 – 2.34 (m, 8H), 2.32 (m, 10H), 2.13 (s, 24H), 2.10 (s, 6H), 2.05 (s, 24H), 2.03 (m, 4H), 1.96 (s, 24H), 1.18 (d, *J* = 6.2 Hz, 24H). ^{13}C NMR (100 MHz, Methanol-*d*$_4$) δ 173.75, 173.73, 173.61, 173.49, 173.24, 172.87, 170.67, 137.65, 128.36, 127.88, 124.80, 97.48, 71.00, 70.98, 70.08, 69.93, 69.91, 69.89, 69.86, 69.84, 69.81, 69.71, 69.66, 69.55, 69.16, 69.10, 67.11, 67.04, 39.72, 39.60, 39.52, 39.37, 39.29, 39.20, 39.06, 32.28, 28.37, 28.31, 28.09, 20.92, 20.82, 20.80, 20.73, 17.41. MS-MALDI-TOF (m/z): calculated for, 4147.72; observed, 4148.14, $[M+Na]^+$

Compound 4: Compound **S33** (350 mg, 0.08 mmol) was dissolved in MeOH (15 mL) and was added MeONa (10 μL). The reaction mixture was stirred at room temperature 24 h, after the completion of the reaction, the product mixture was neutralized with acid resin and filtered, washed with MeOH, and then the solvent was concentrated to afford compound **4** (220 mg, 83%). ^{1}H NMR (400 MHz, Methanol-*d*$_4$) δ 4.80 – 4.73 (m, 8H), 4.42 (m, 4H), 4.29 – 4.19 (m, 2H), 4.04 (m, 2H), 3.94 (m, 2H), 3.90 – 3.72 (m, 21H), 3.68 (m, 10H), 3.66 (m, 41H), 3.51 – 3.34 (m, 28H), 2.60 – 2.50 (m, 2H), 2.43 – 2.29 (m, 12H), 2.04 (m, 14H), 1.29 (d, *J* = 6.1 Hz, 24H). ^{13}C NMR (100 MHz, Methanol-*d*$_4$) δ 173.86, 173.69, 173.65, 173.64, 173.59, 173.56, 173.55, 173.53, 173.36, 173.25, 173.13, 172.91, 172.87, 172.82, 172.46, 172.33, 170.36, 170.20, 100.33, 100.30, 72.62, 70.99, 70.96, 70.82, 69.87, 69.85, 69.28, 69.22, 69.13, 69.07, 68.46, 66.33, 42.13, 39.23, 39.08, 31.84, 16.79, 16.76. MS-MALDI-TOF (m/z): calculated for 3139.46, observed, 3139.67, $[M+Na]^+$

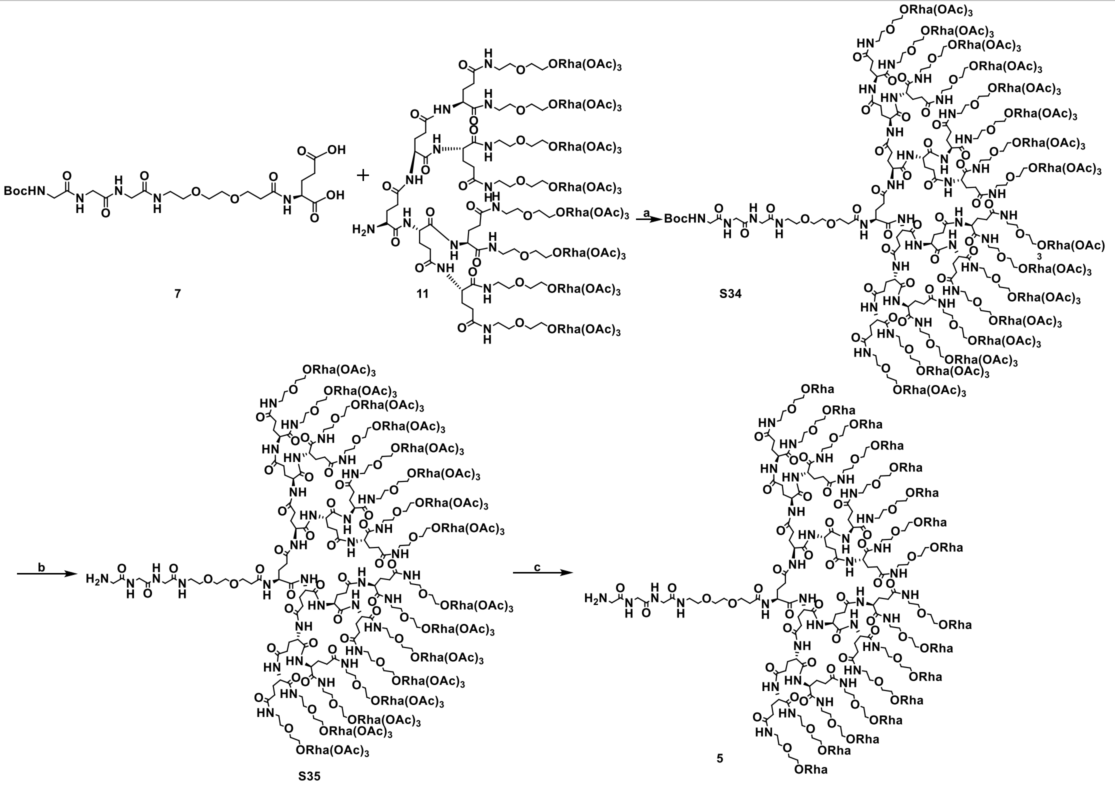

(a) HBTU, HOBt, DIPEA, DMF, r.t, 24 h, 50%; (b) 25% TFA/DCM, 0ºC, 2 h, 93%; (c) MeOH, MeONa, r.t, 18 h, 95%.

Compound S34: To a solution of the compound **7** (60 mg, 0.1 mmol) in DMF (10 mL) and added compound **11** (870 mg, 0.23 mmol), HBTU (80 mg, 0.23 mmol), DIPEA (0.1 mL, 1 mmol) and HOBt (30 mg, 0.23 mmol). The reaction mixture was stirred at r.t. for 24 h, after the completion of the reaction, the reaction was concentrated and extracted with DCM, washed with saturated NaCl solution, the organic layer was dried over anhydrous Na_2SO_4, then concentrated and was purified by silica gel chromatography to give the compound **S34** (400 mg, 50%). ^{1}H NMR (400 MHz, Methanol-*d₄*) δ 5.34 – 5.18 (m, 32H), 5.02 (m, 16H), 4.86 (m, 16H), 4.47 – 4.25 (m, 11H), 3.96 (m, 20H), 3.83 (m, 22H), 3.71 (m, 46H), 3.61 (m, 40H), 3.51 (m, 12H), 3.40 (m, 26H), 2.38 (s, 32H), 2.16 (s, 48H), 2.12 (m, 10H), 2.08 (s, 48H), 2.02 (m, 10H), 1.98 (s, 48H), 1.93 (m, 8H), 1.47 (s, 9H), 1.21 (d, *J* = 6.3 Hz, 48H). ^{13}C NMR (100 MHz, Methanol-*d₄*) δ 173.50, 173.38, 173.35, 170.33, 173.31, 170.27, 173.22, 97.49, 97.47, 70.81, 69.72, 69.70, 69.68, 69.64, 69.33,69.22, 69.20, 66.89, 66.86, 66.76, 66.24, 65.51, 62.93, 39.19, 39.03, 31.80, 31.26, 31.09, 27.96, 27.94, 27.83, 27.75, 27.46, 19.45, 19.40, 19.37, 19.36, 19.34, 16.54, 16.50, 16.49, 14.07. MS-MALDI-TOF (m/z): calculated for, 8130.38; observed, 8075.95 $[M-56+H]^+$

Compound S35: To a solution of the compound **S34** (380 mg, 0.04 mmol) in DCM (10 mL), then 50% TFA/DCM (10 mL) was added dropwise at 0ºC, and the reaction mixture was stirred at 0ºC for 2 h. After the completion of the reaction, the reaction was diluted with toluene (5 mL), and concentrated in vacuo. The residual TFA was removed from the crude residue by azeotroping with toluene (3×5 mL) and was concentrated to give the compound **S35**[3] (350 mg, 93%). ^{1}H NMR (400 MHz, Methanol-*d₄*) δ 5.27 – 5.19 (m, 32H), 5.02 (m, 16H), 4.84 (m, 16H), 4.37 (m, 11H), 4.01 – 3.90 (m, 20H), 3.84 (m, 25H), 3.73 – 3.67 (m, 48H), 3.61 (m, 42H), 3.40 (m, 31H), 2.38 (m, 32H), 2.16 (s, 48H), 2.11 (m, 12H), 2.07 (s, 48H), 2.03 (m, 10H), 1.98 (s, 48H), 1.91 (m, 6H), 1.21 (d, *J* = 6.2 Hz, 48H). ^{13}C NMR (100 MHz, Methanol-*d₄*) δ 173.52, 173.48, 173.44, 173.38, 173.37, 170.33, 170.33, 170.28, 97.47, 70.78, 70.63, 69.63, 69.33, 66.85, 66.24, 62.92, 39.19, 39.03, 19.44, 19.37, 16.52. MS-MALDI-TOF (m/z): calculated for 8053.53; observed, 8053.72, $[M+Na]^+$

Compound 5: Compound **S35** (340 mg, 0.037 mmol) was dissolved in MeOH (10 mL) and added MeONa (10 µL), the reaction mixture was stirred at r.t. for 24 h. After the completion of the reaction, the product mixture was neutralized with acid resin and filtered, washed with MeOH, and then the solvent was concentrated to afford compound **5** (240 mg, 95%).

^{1}H NMR (400 MHz, Methanol-*d₄*) δ 4.74 (m, 16H), 4.47 – 4.27 (m, 11H), 3.86 – 3.82 (m, 16H), 3.79 – 3.75 (m, 16H), 3.69 – 3.60 (m, 80H), 3.60 – 3.51 (m, 49H), 3.48 – 3.43 (m, 8H), 3.43 – 3.34 (m, 44H), 2.36 (m, 30H), 2.17 – 2.01 (m, 18H), 2.00 – 1.86 (m, 12H), 1.27 (d, *J* = 6.3 Hz, 48H). ^{13}C NMR (100 MHz, Methanol-*d₄*) δ 173.69, 173.55, 173.26, 173.13, 173.09, 172.61, 100.32, 72.63, 71.00, 70.83, 70.12, 69.87, 69.83, 69.13, 68.47, 66.35, 62.94, 52.96, 48.50, 48.22, 48.07, 47.93, 47.91, 47.79, 47.65, 47.51, 47.48, 47.36, 47.22, 39.25, 39.11, 31.87, 16.84, 16.80, 16.71. MS-MALDI-TOF (m/z): calculated for 6036.81; observed, 6036.63, $[M+Na]^+$

Supplementary Figures

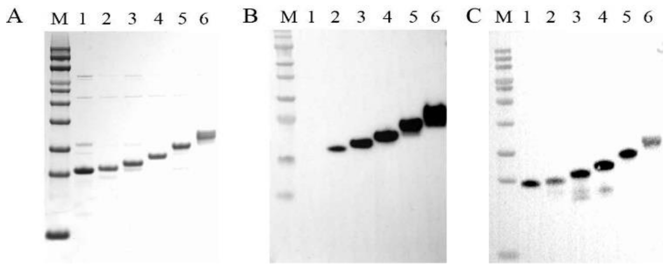

Figure S1: Characterization of nanobody conjugates (**D1**-**D5**); Lane M: marker, Lane 1: 7D12, Lane 2: conjugate **D1**, Lane 3: conjugate **D2**, Lane 4: conjugate **D3**, Lane 5: conjugate **D4**, Lane 6: conjugate **D5**. (A) SDS-PAGE analysis of purified 7D12, conjugates **D1**, **D2**, **D3**, **D4**, **D5**; (B) western blot using *anti*-Rha antibody; (C) western blot using *anti*-myc antibody.

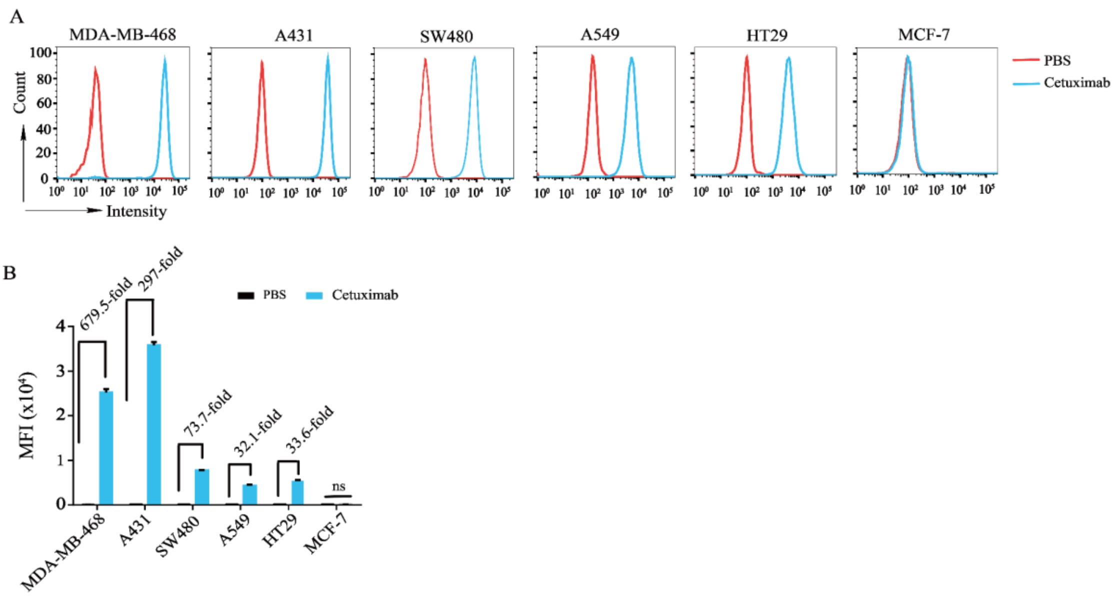

Figure S2: Evaluation of EGFR expression on different cell lines. (A) Flow cytometry assays and (B) the corresponding MFIs of cells treated with PBS or cetuximab. Data are reported as mean ± SD (n=3).

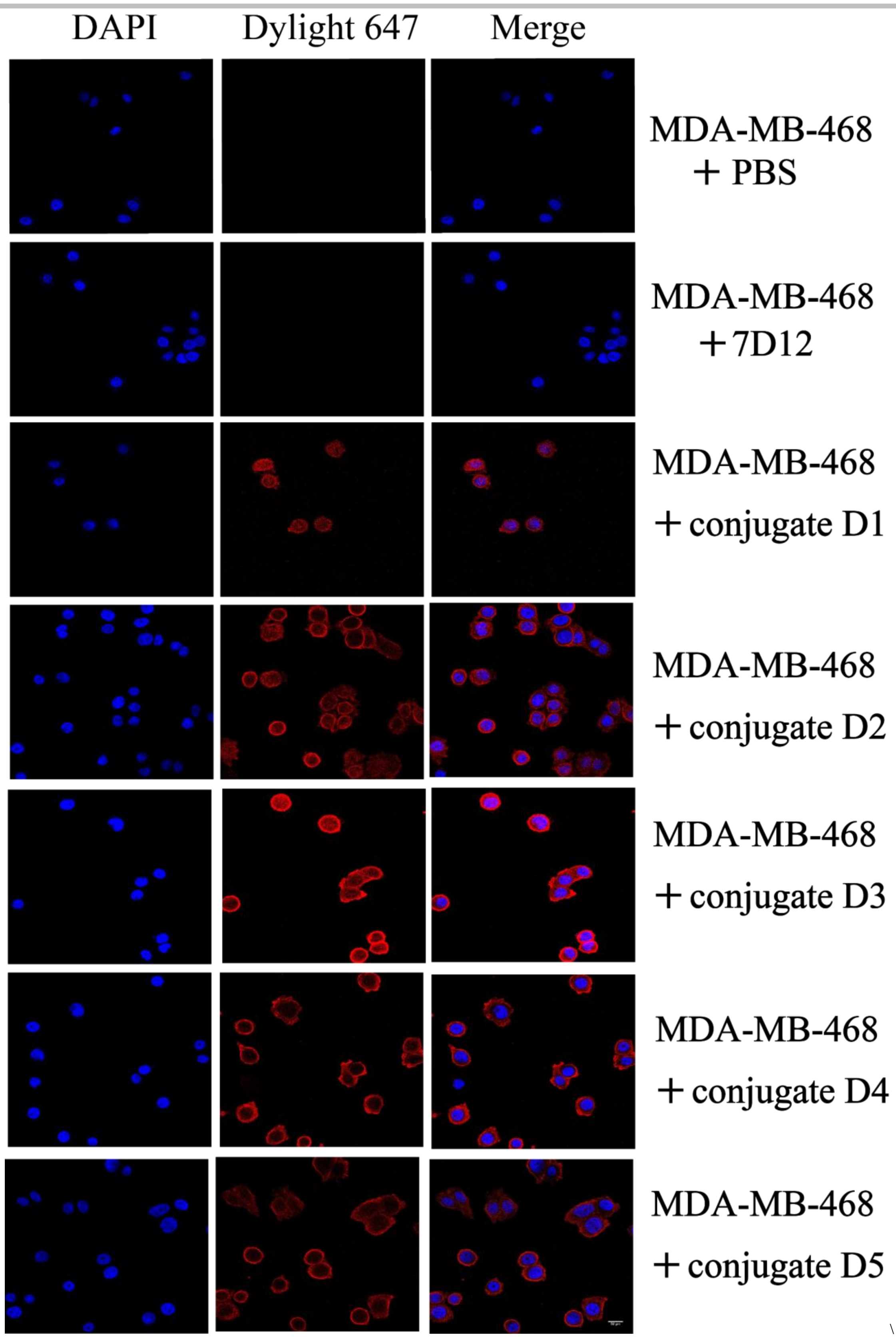

Figure S3: Confocal laser scanning microscopy images of MDA-MB-468 cells treated with PBS, 7D12, monovalent conjugate **D1** and multivalent conjugates (**D2** to **D5**), Scale bar: 50 μm.

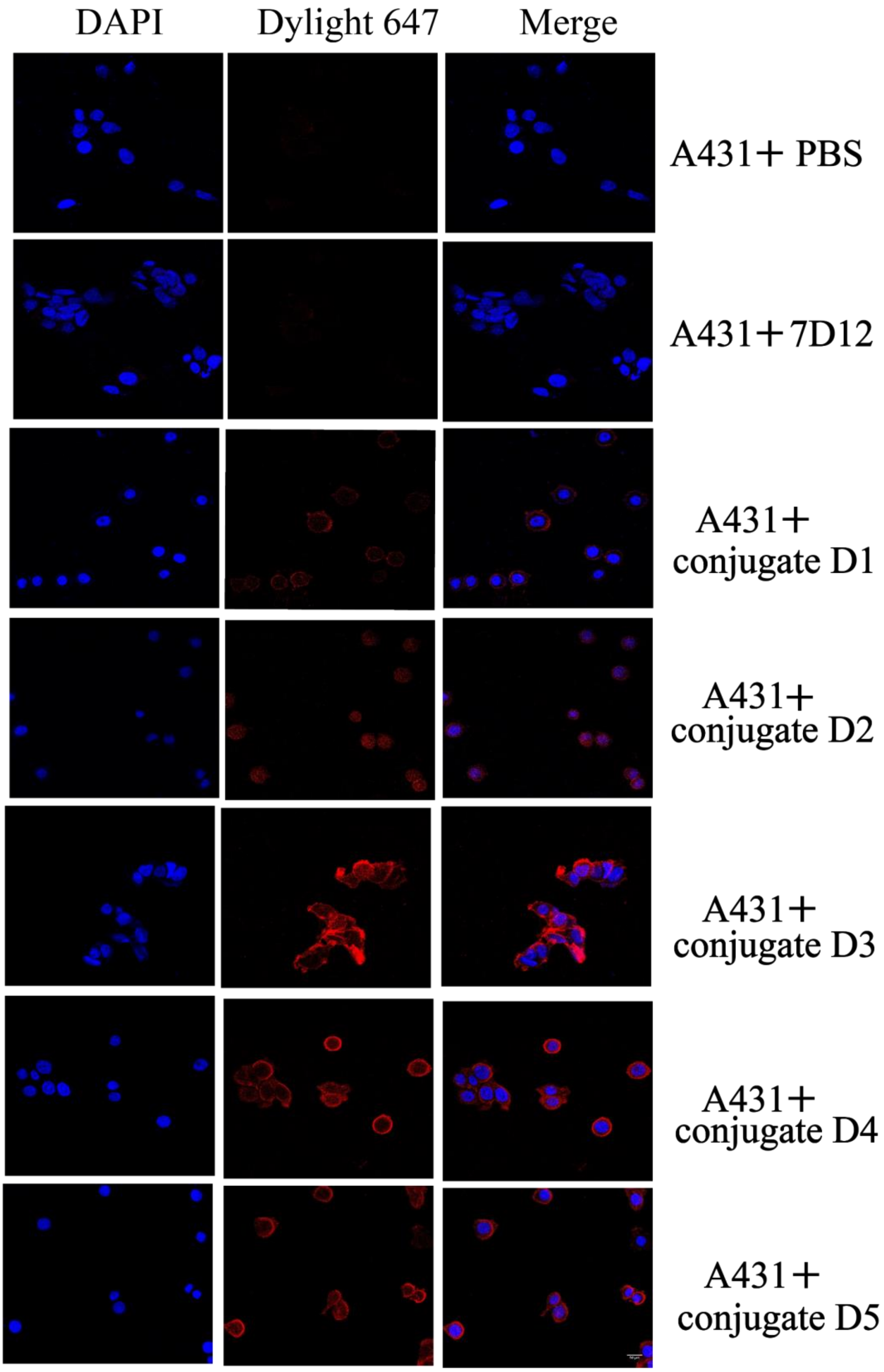

Figure S4: Confocal laser scanning microscopy images of A431 cells treated with PBS, 7D12, monovalent conjugate **D1** and multivalent conjugates (**D2** to **D5**), Scale bar: 50 μm.

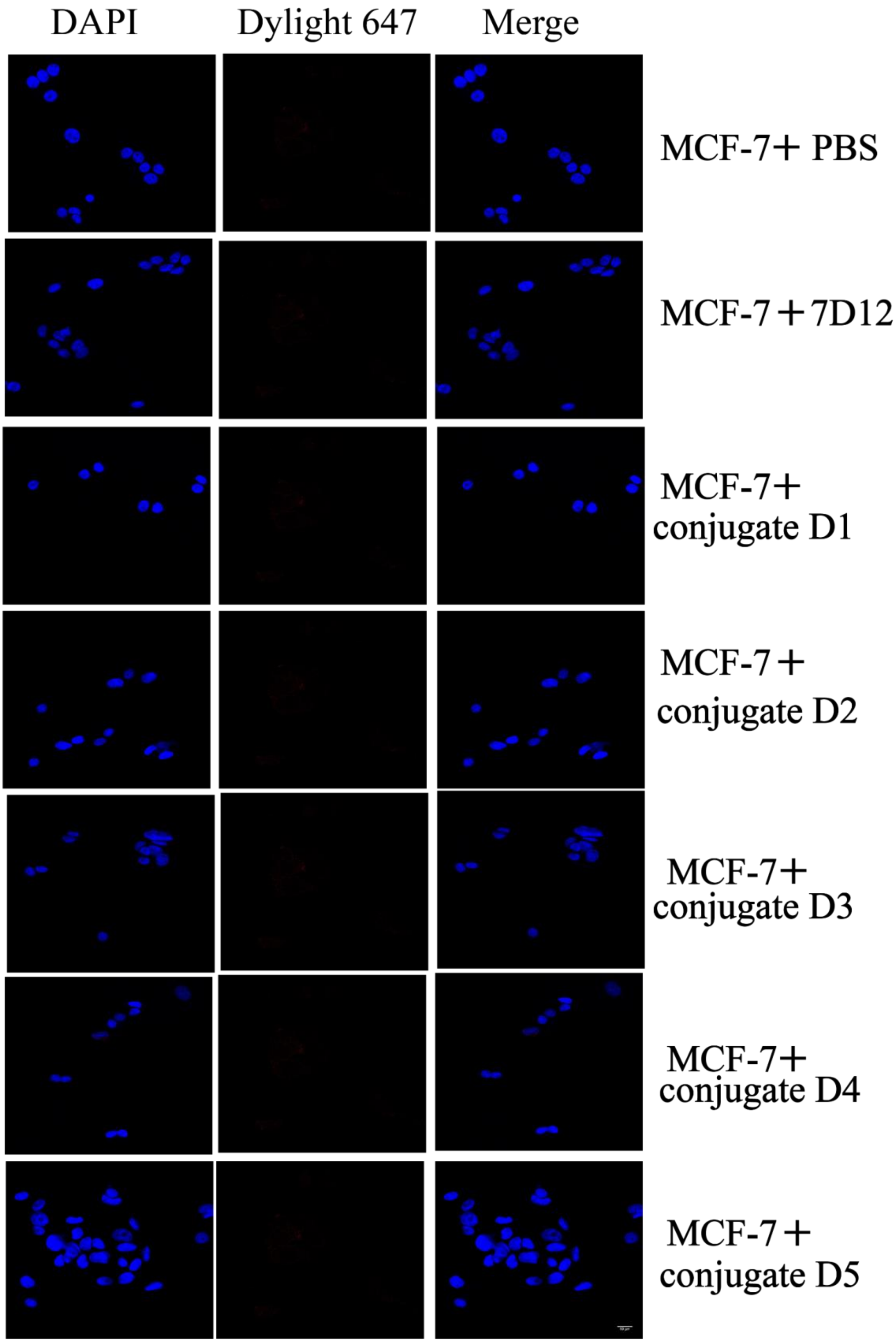

Figure S5: Confocal laser scanning microscopy images of MCF-7 cells treated with PBS, nanobody 7D12, monovalent conjugate **D1**, multivalent conjugates (**D2** to **D5**), Scale bar: 50 μm.

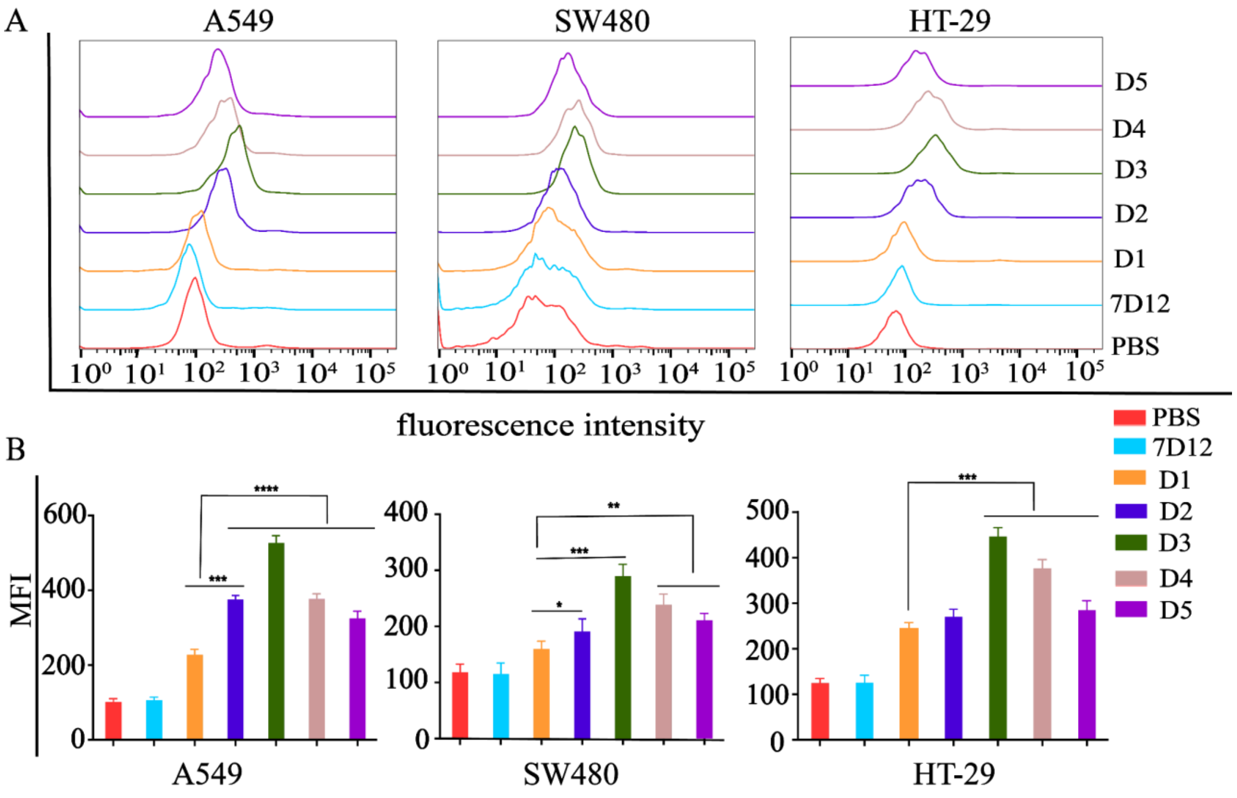

Figure S6: Antibodies recruiting assays. (A) the histograms and (B) the corresponding MFI of cells treated with PBS, 7D12, monovalent conjugate **D1**, multivalent conjugates (**D2** to **D5**). Data are reported as mean ± SD (n=3) and the statistical significance is determined using Student's t-test (two-tailed). *: *P*< 0.05, **: *P*< 0.01, ***: *P*< 0.001, ****: *P*< 0.0001.

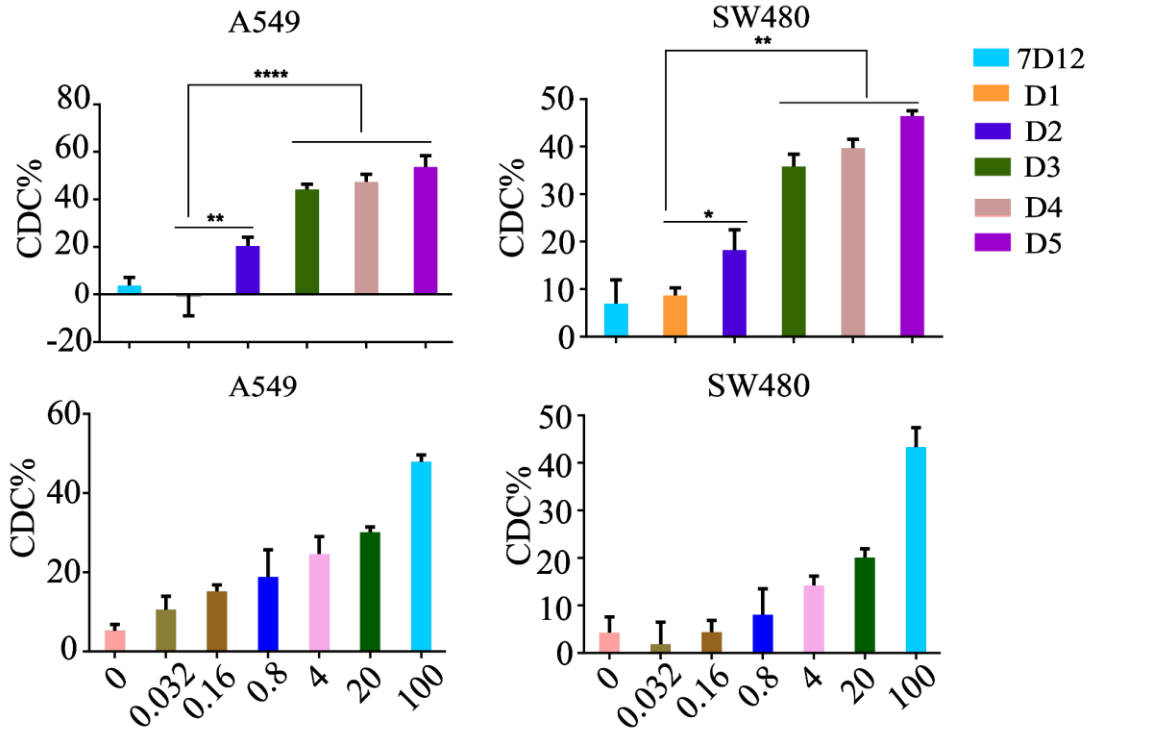

Figure S7: *In vitro* CDC assay (A) CDC mediated by 100 nM of 7D12, monovalent conjugate **D1**, multivalent conjugates (**D2** to **D5**); (B) CDC mediated by different concentrations of conjugates **D5** (0-100 nM). Data are reported as mean ± SD (n=3) and the statistical significance is determined using Student's t-test (two-tailed). *: *P*< 0.05, **: *P*< 0.01, ****: *P*< 0.0001.

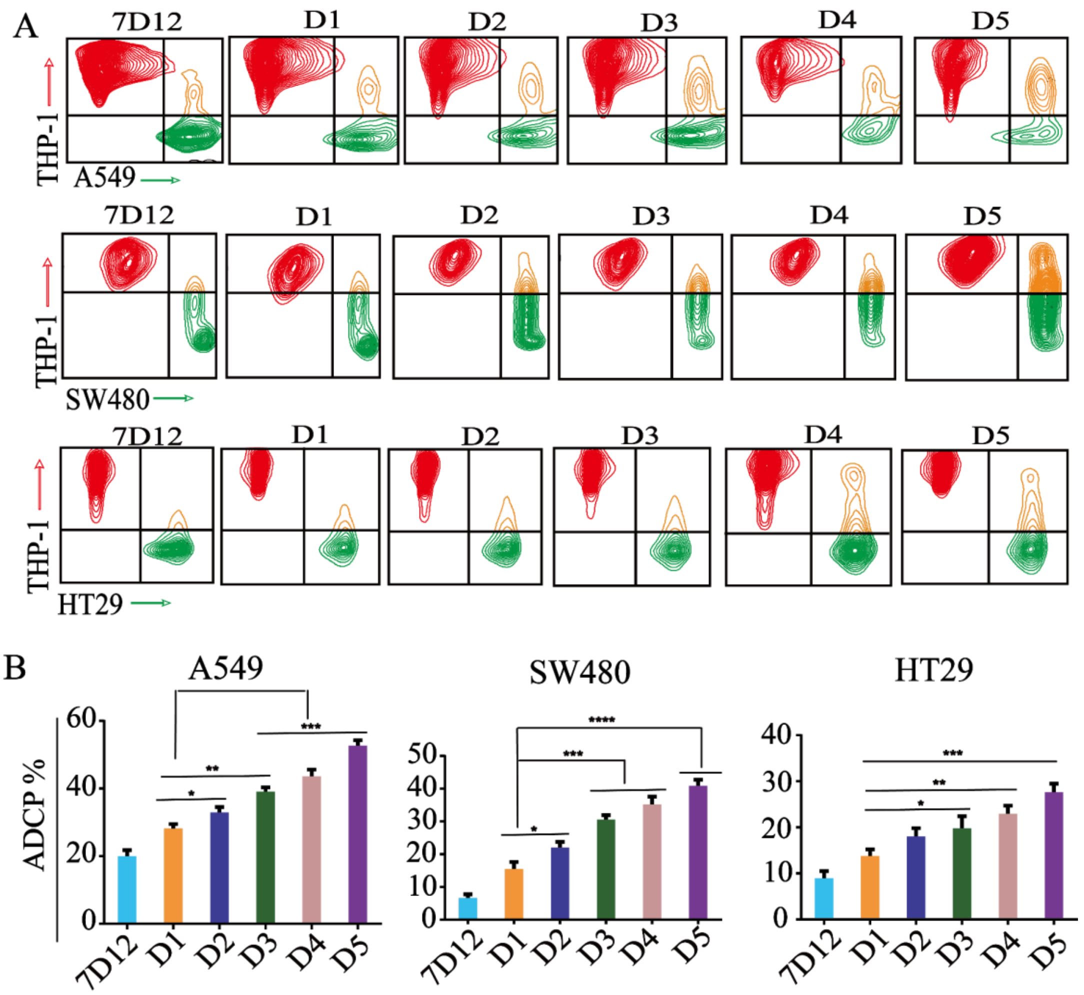

Figure S8: *In vitro* ADCP assays. (A) Flow cytometry assays and (B) the corresponding phagocytosis of target cells treated with 100 nM of 7D12, monovalent conjugate **D1**, multivalent conjugates (**D2** to **D5**); Data are reported as mean ± SD (n=3) and the statistical significance is determined using Student's t-test (two-tailed). *: *P*< 0.05, **: *P*< 0.01, ***: *P*< 0.001, ****: *P*< 0.0001.

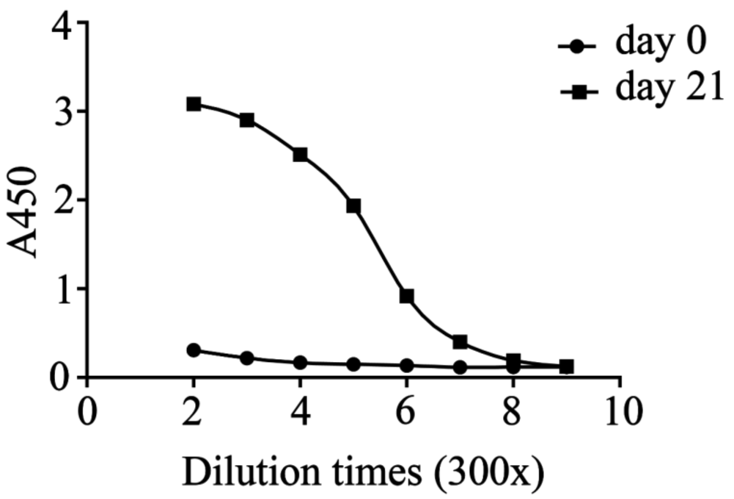

Figure S9: The titer of anti-Rha antibodies in Balb/c mice. The titer of anti-Rha antibodies in Balb/c mice at 0 and day 21 was determined by ELISA. The calculated titer at day 21 was about $33x10^5$

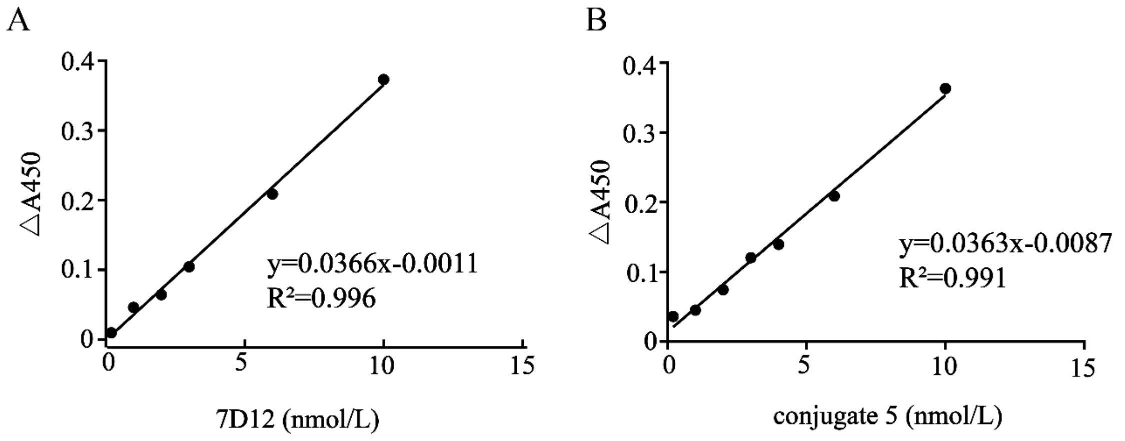

Figure S10: The standard curves for 7D12 and conjugate **D5** concentrations determination. The standard curves of (A) 7D12 and (B) conjugate **D5** were generated by ELISA using human recombinant EGFR as the coating antigen. $\triangle A450 = A_x - A_0$, where A_x is the OD450 value of samples containing x nM of 7D12 or conjugate **D5**. A_0 is the OD450 value of samples containing 0 nM of 7D12 or conjugate **D5**.

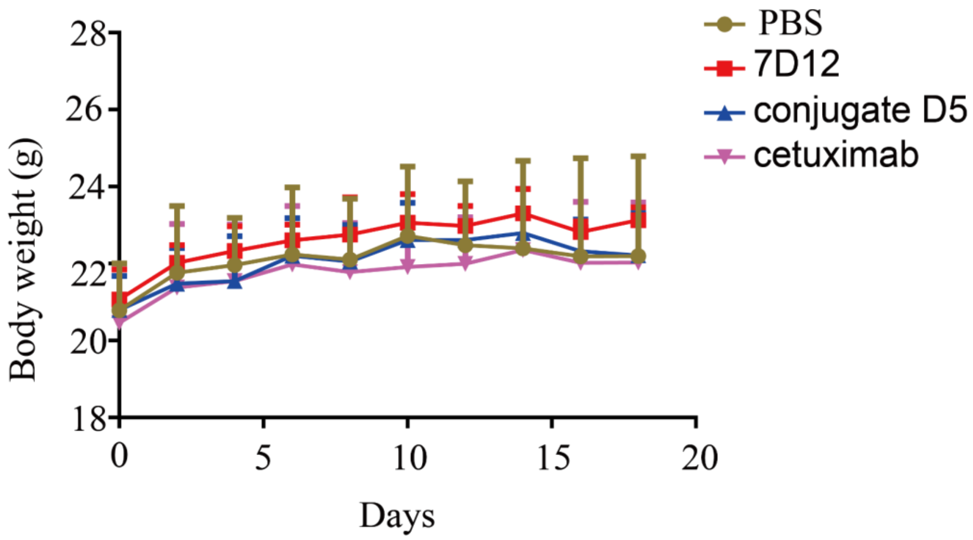

Figure S11: The body weight of Balb/c nude mice in each group. Data are reported as mean ± SD (n=3 or 4).

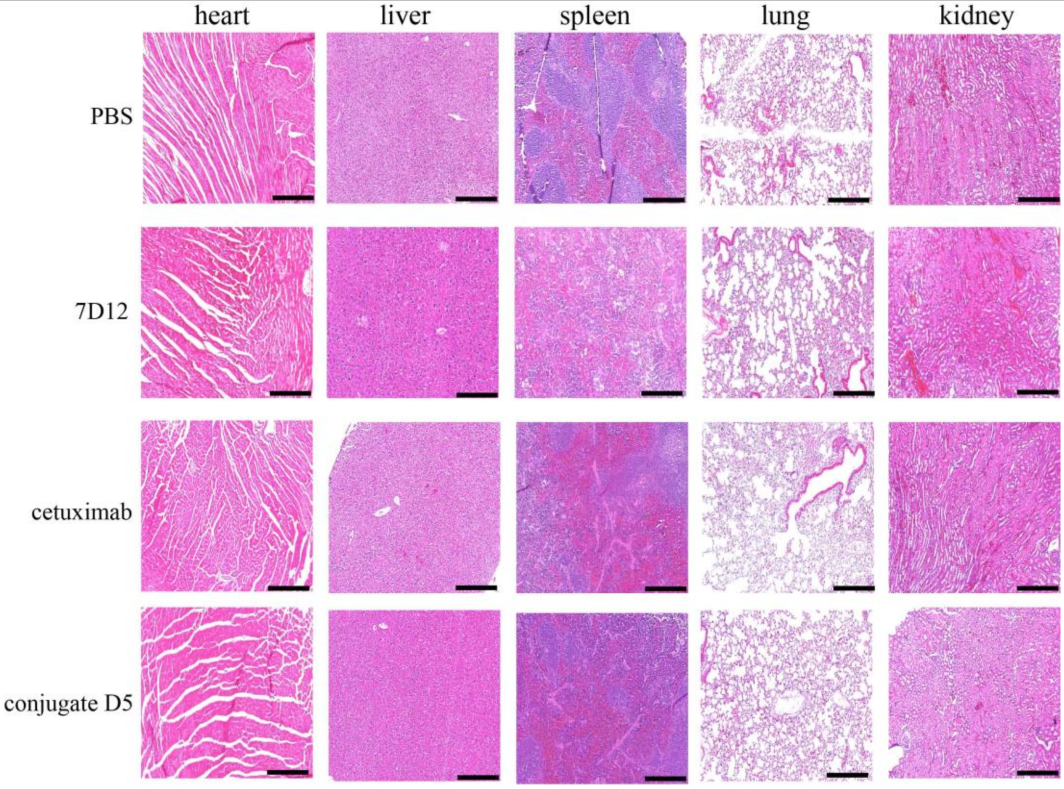

Figure S12: Histological assays of different tissues from mice treated with PBS, 7D12, cetuximab and conjugate D5, Scale bar: 50 μm.

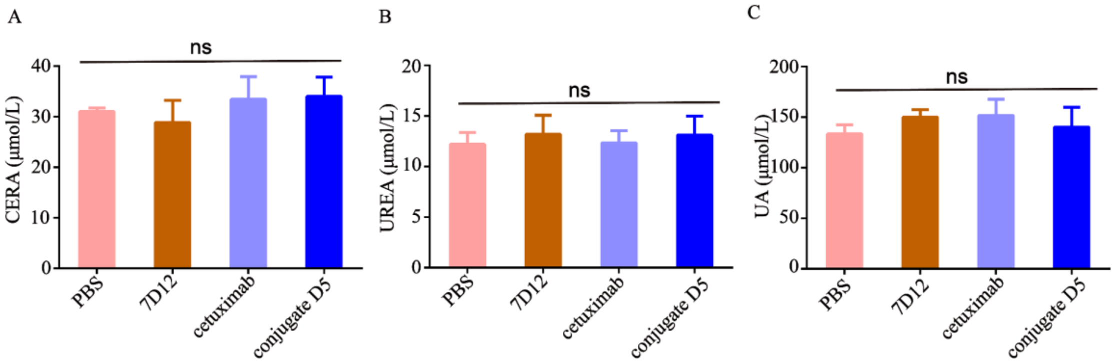

Figure S13: Evaluation the renal-related indexes of all groups, (A) creatinine (CREA), (B) urea (UREA), and (C) uric acid (UA). Data are reported as mean ± SD (n=3 or 4).

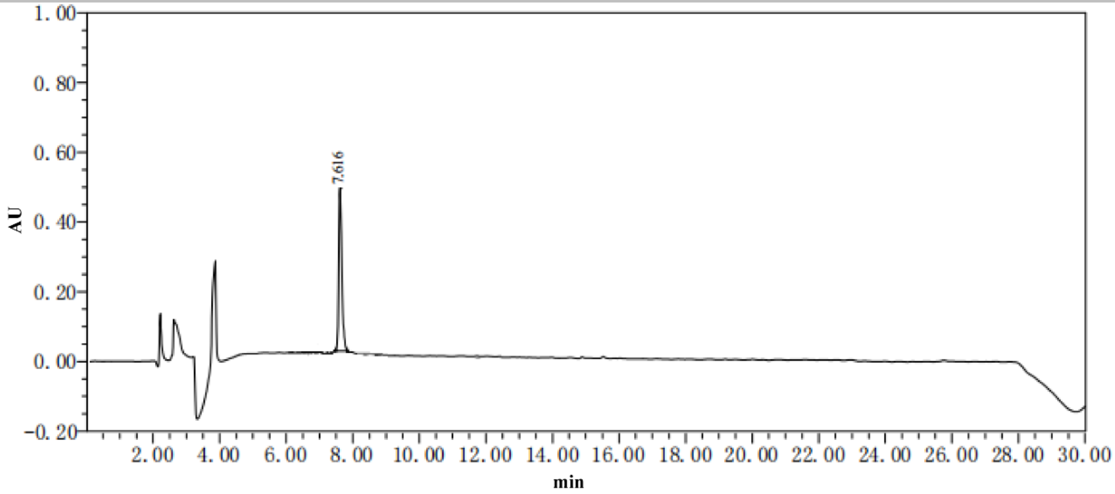

Figure S14: The HPLC analysis of compound 1 (purity: 97.7%)

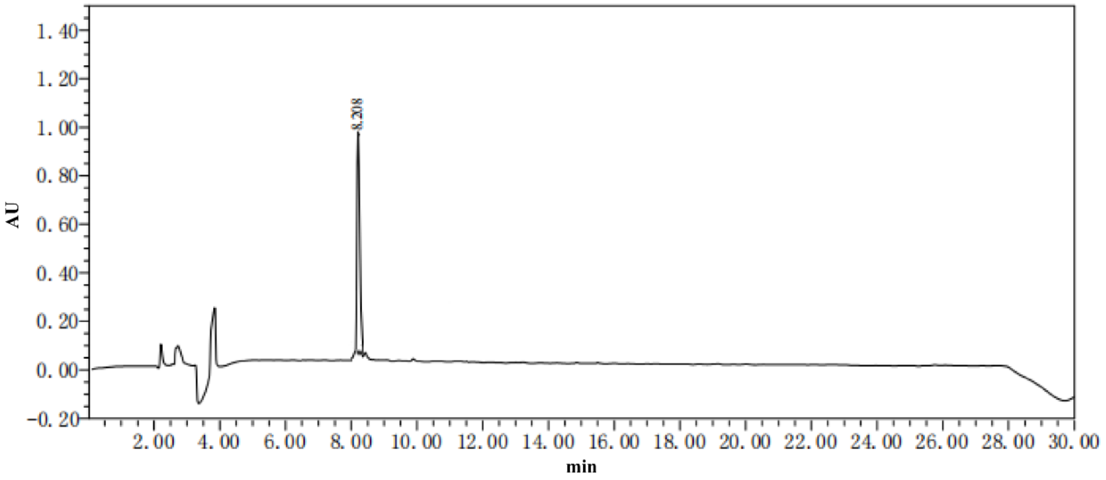

Figure S15: The HPLC analysis of compound 2 (purity: 95.2%)

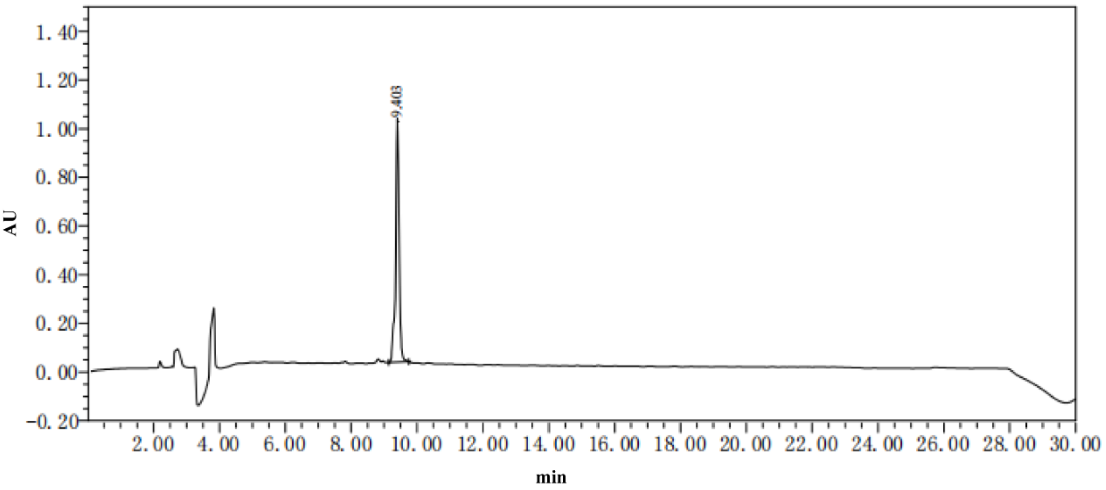

Figure S16: The HPLC analysis of compound 3 (purity: 96.8%)

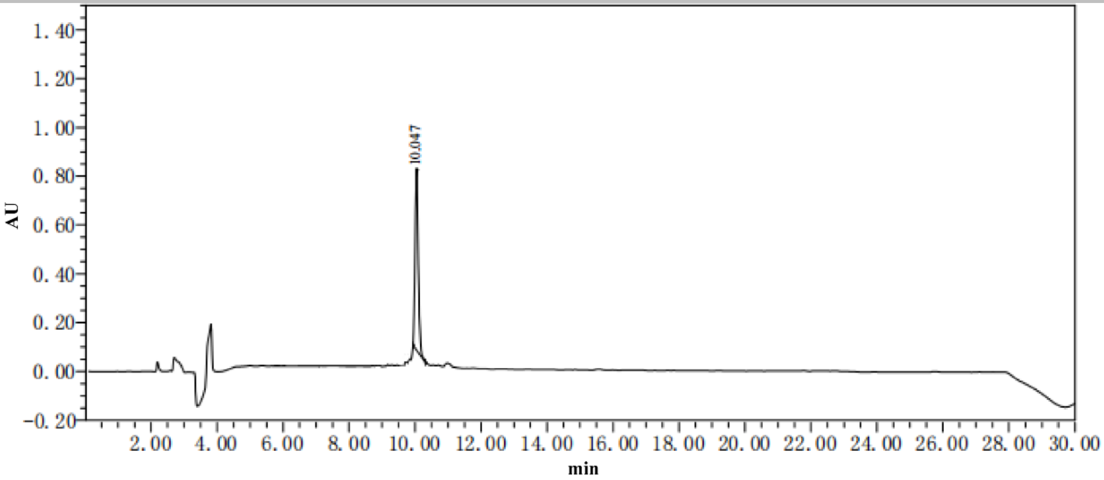

Figure S17: The HPLC analysis of compound 4 (purity: 96.1%)

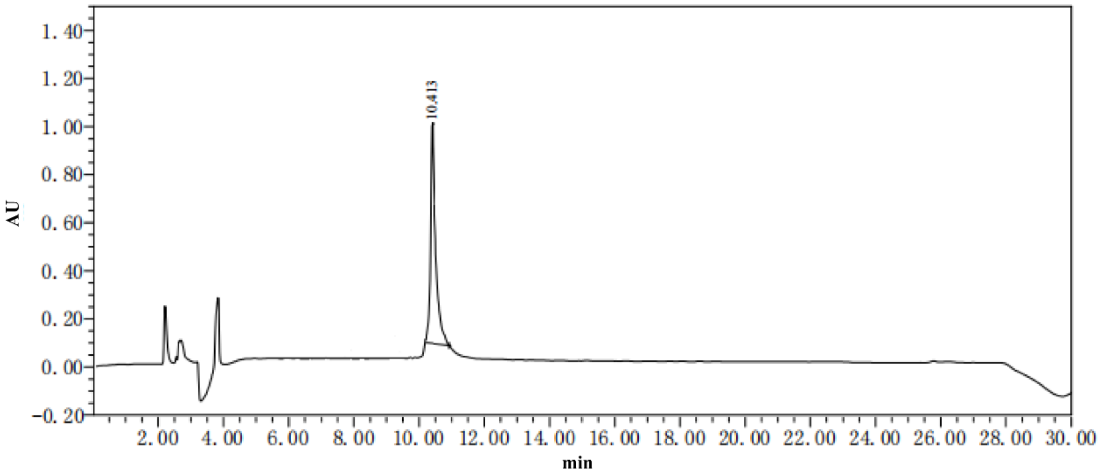

Figure S18: The HPLC analysis of compound 5 (purity: 99.2%)

NMR Spectra

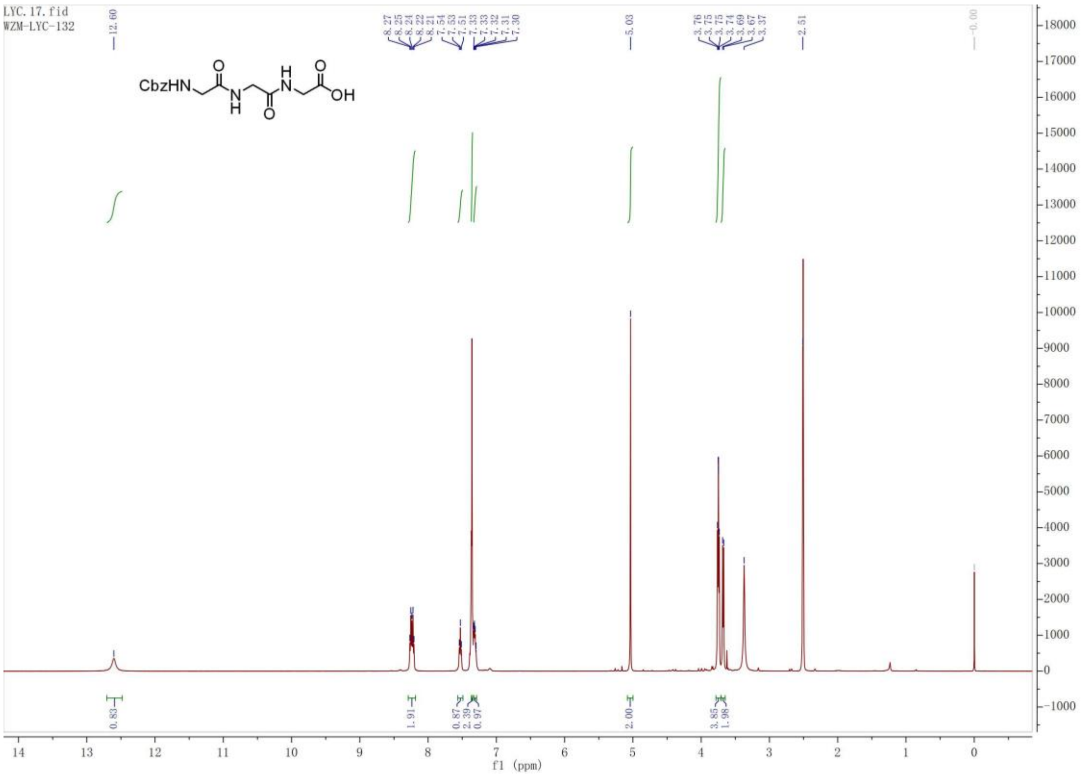

^{1}H-NMR spectrum of compound **S2**.

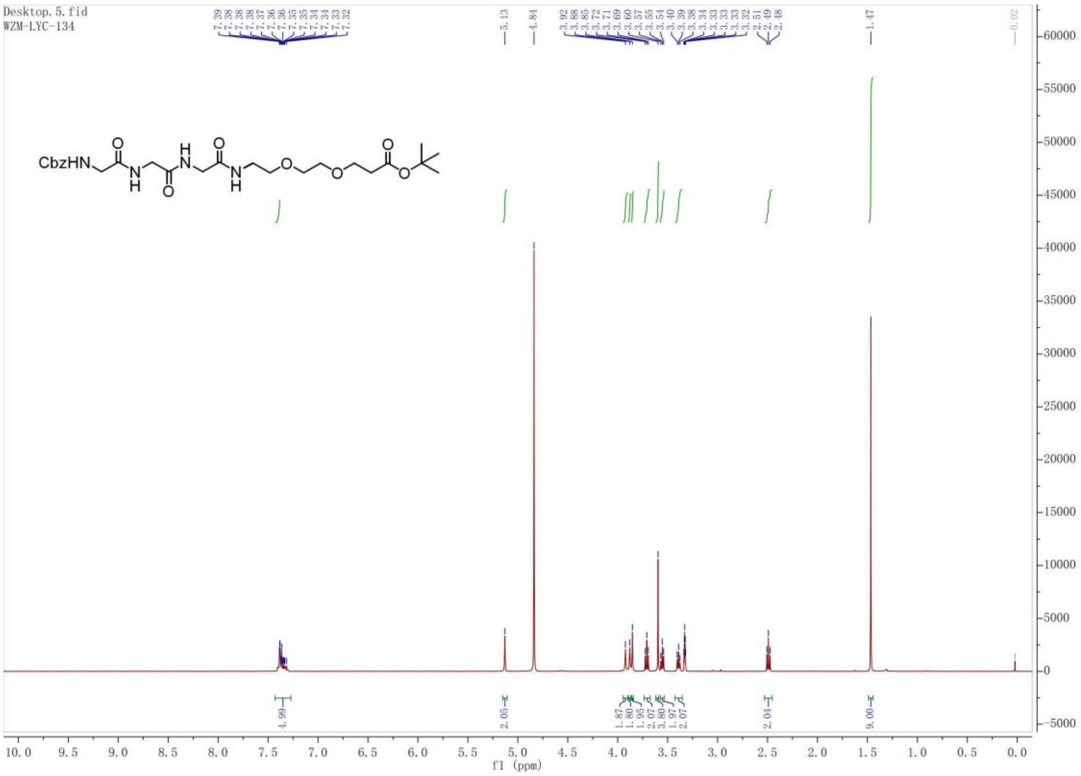

^{1}H-NMR spectrum of compound **S3**

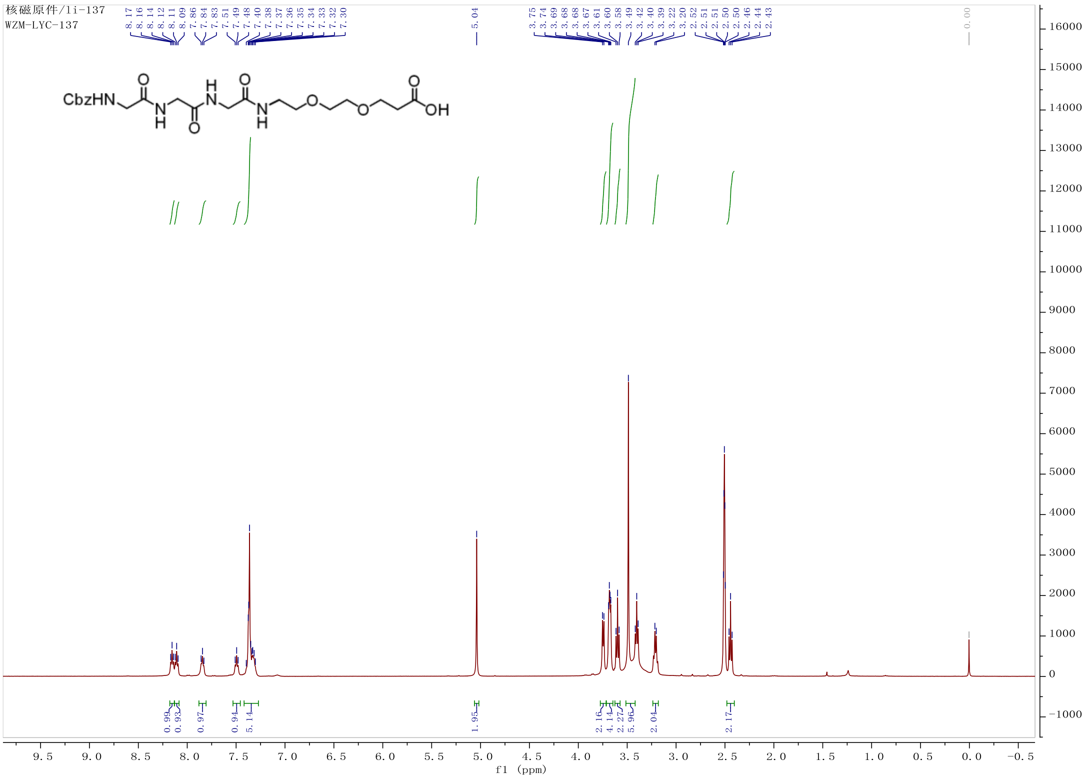

^{1}H-NMR spectrum of compound **6**

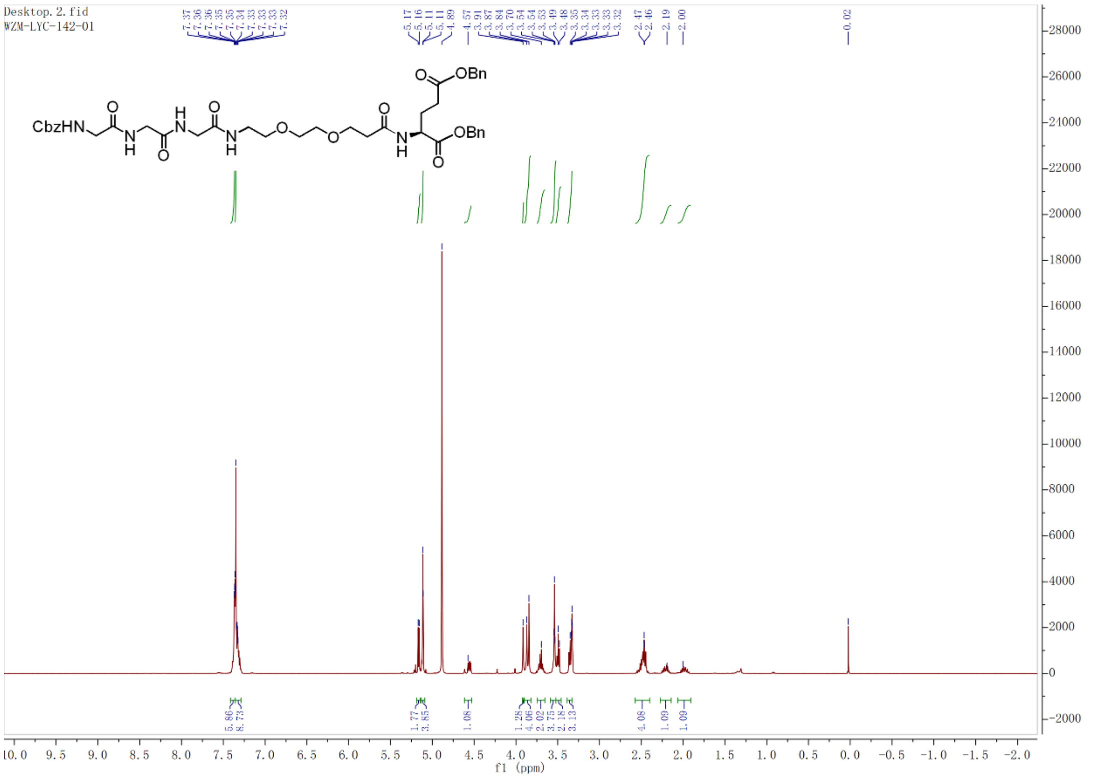

^{1}H-NMR spectrum of compound **S5**.

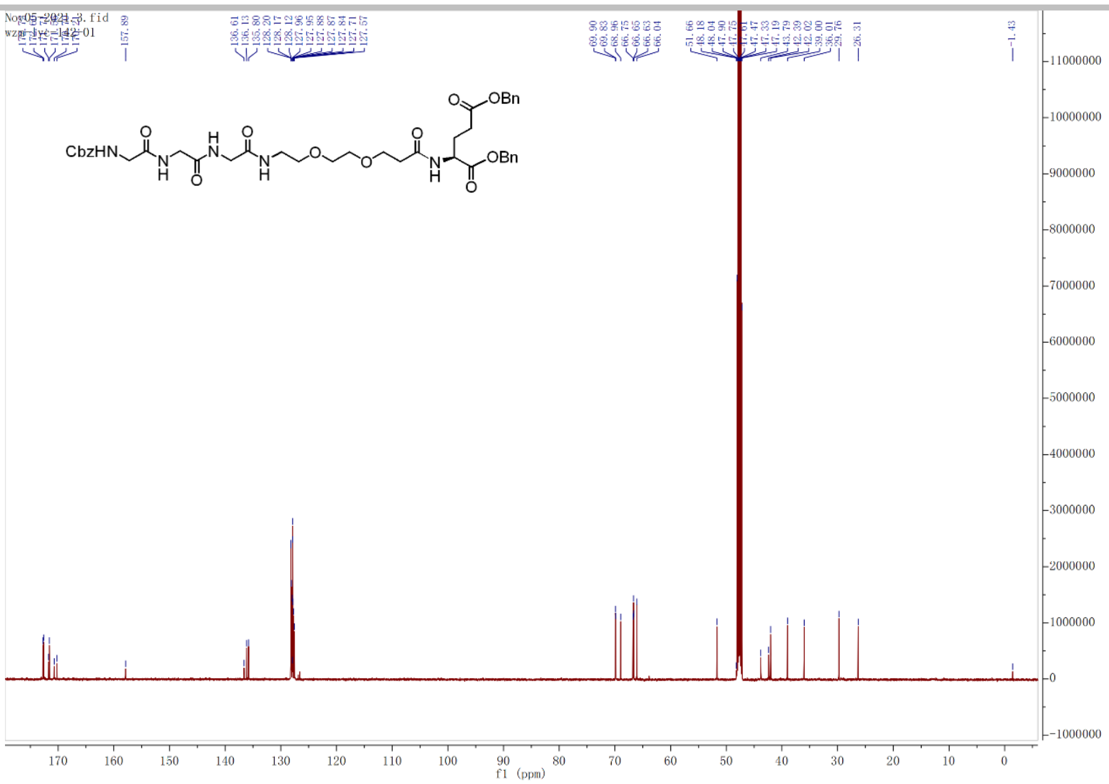

^{13}C-NMR spectrum of compound **S5**

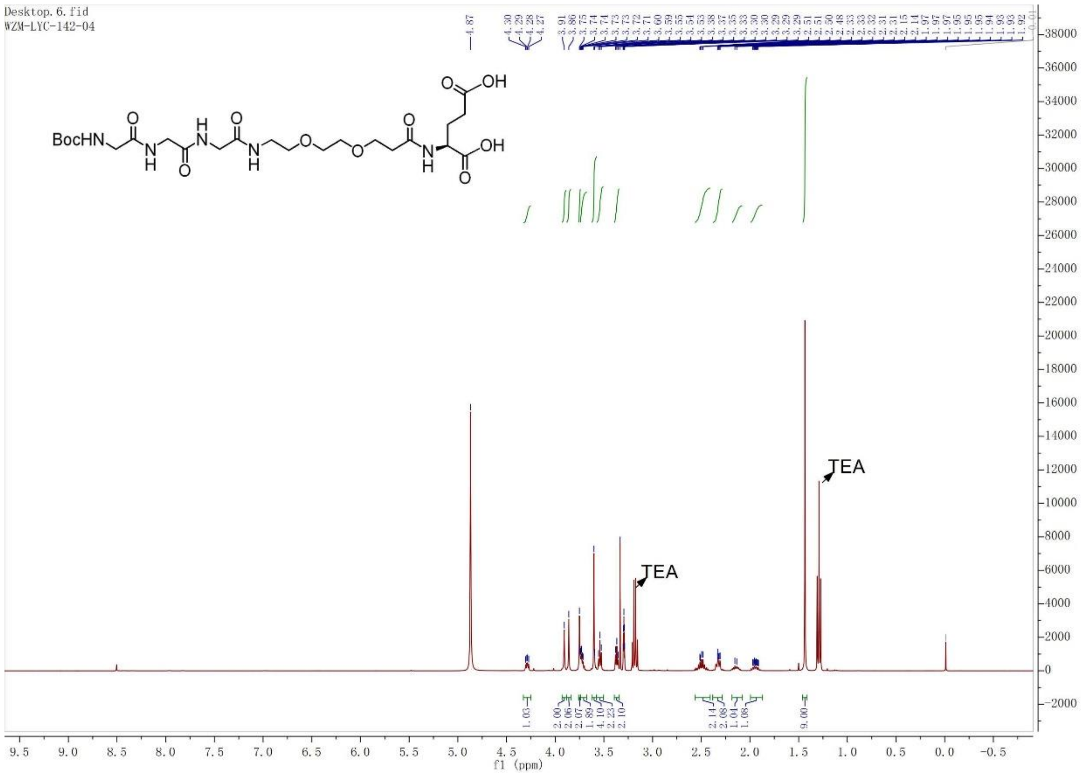

^{1}H-NMR spectrum of compound **7**.

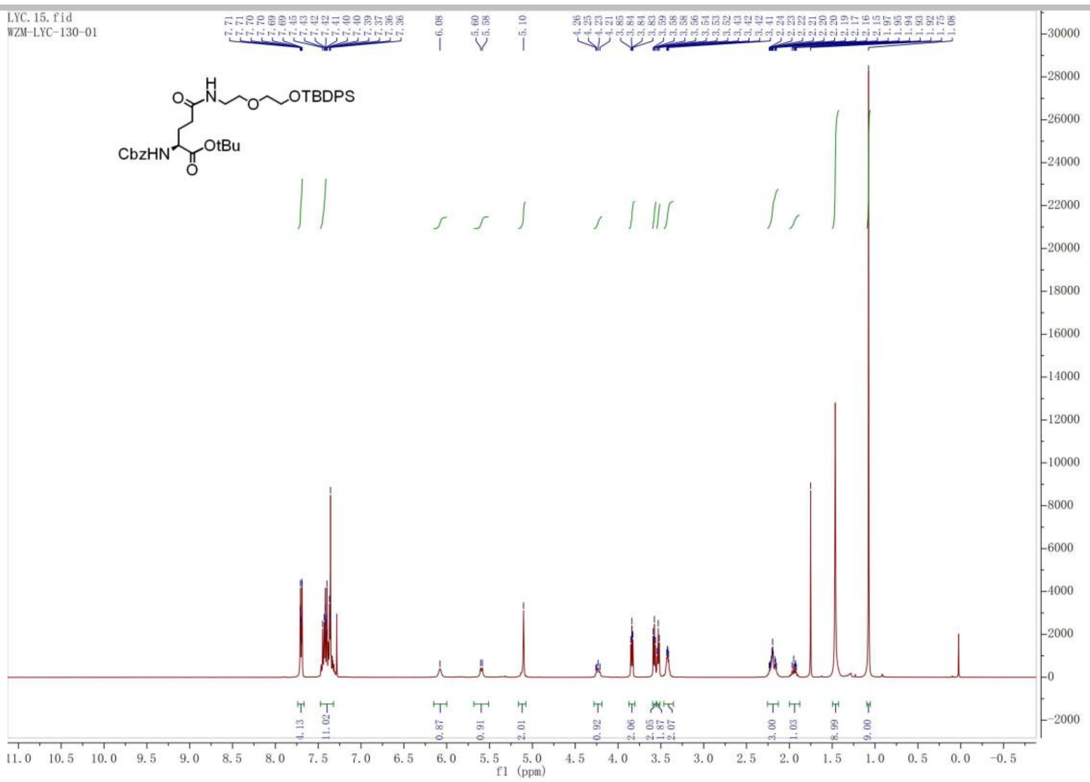

^{1}H-NMR spectrum of compound **S8**.

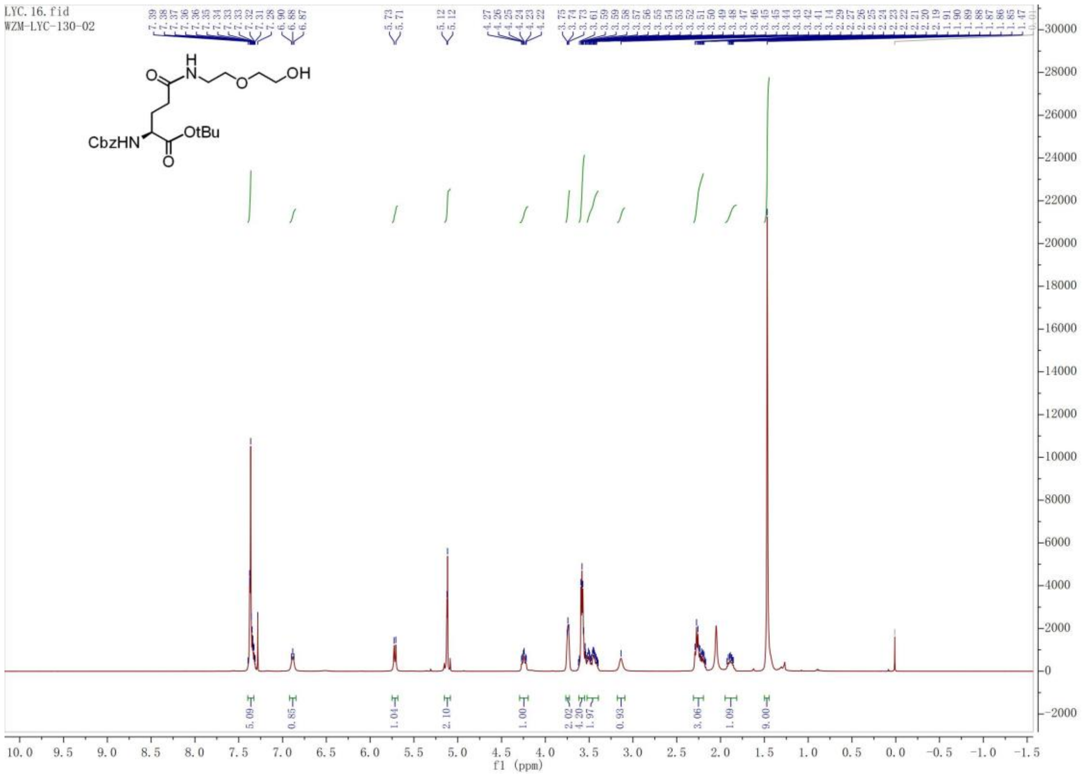

^{1}H-NMR spectrum of compound **S9**

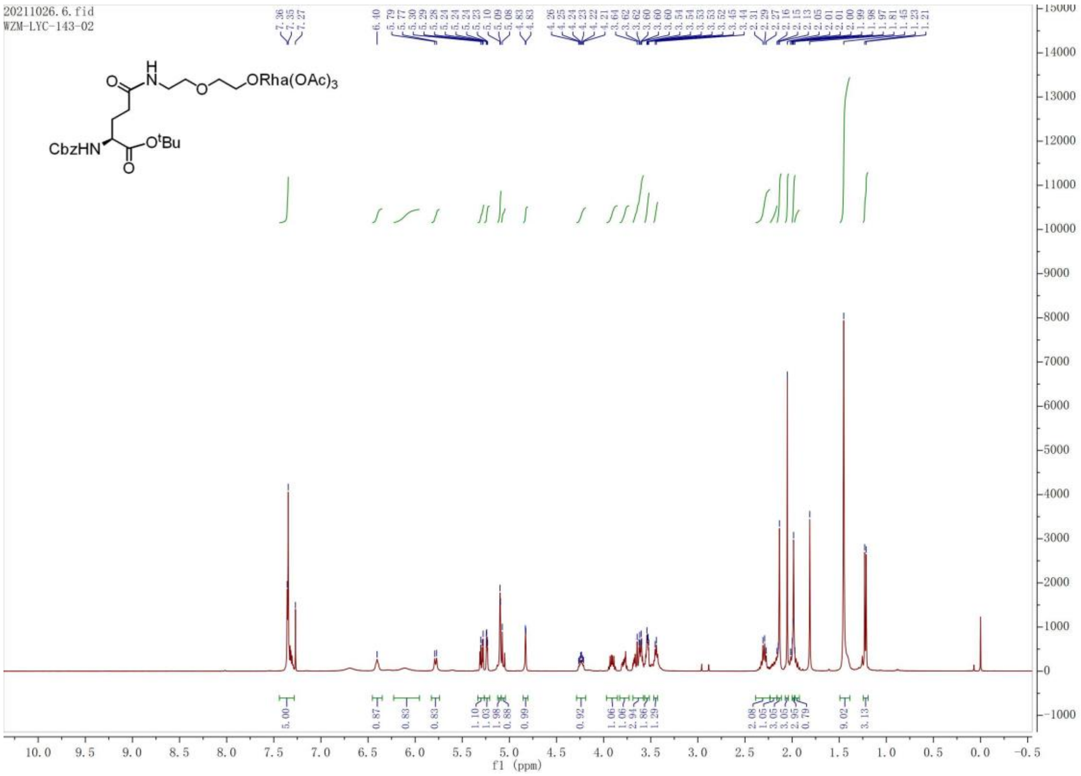

^{1}H-NMR spectrum of compound **S13**

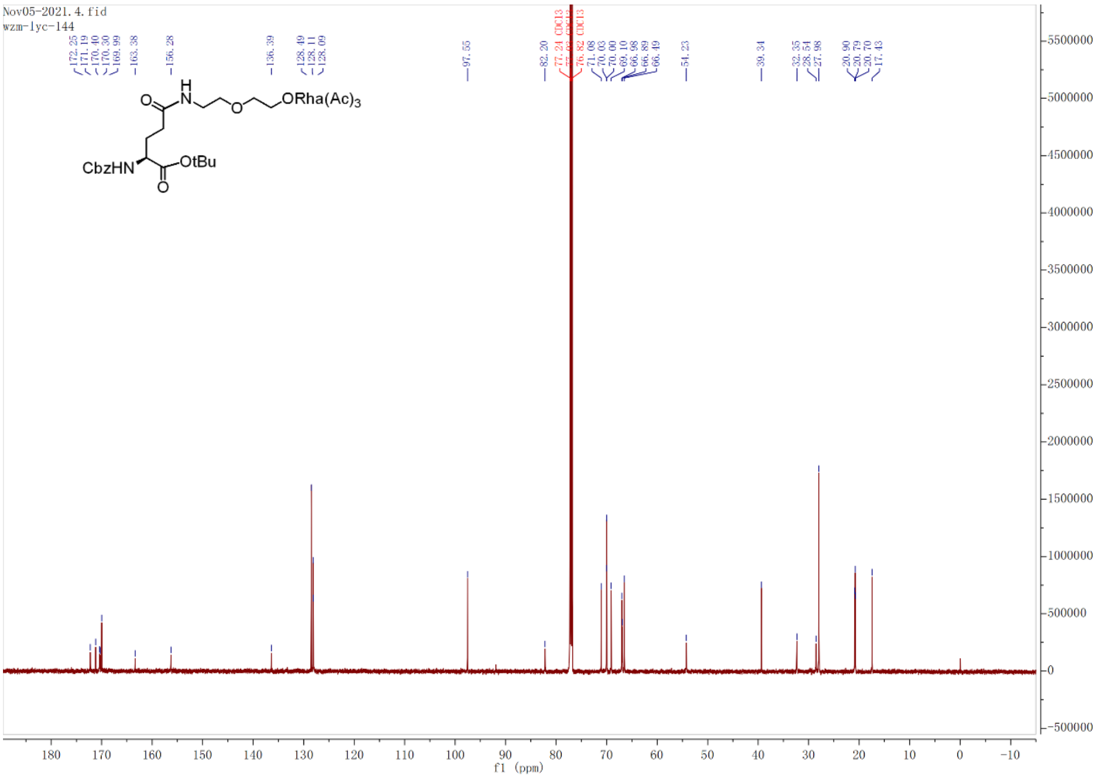

^{13}C-NMR spectrum of compound **S13**

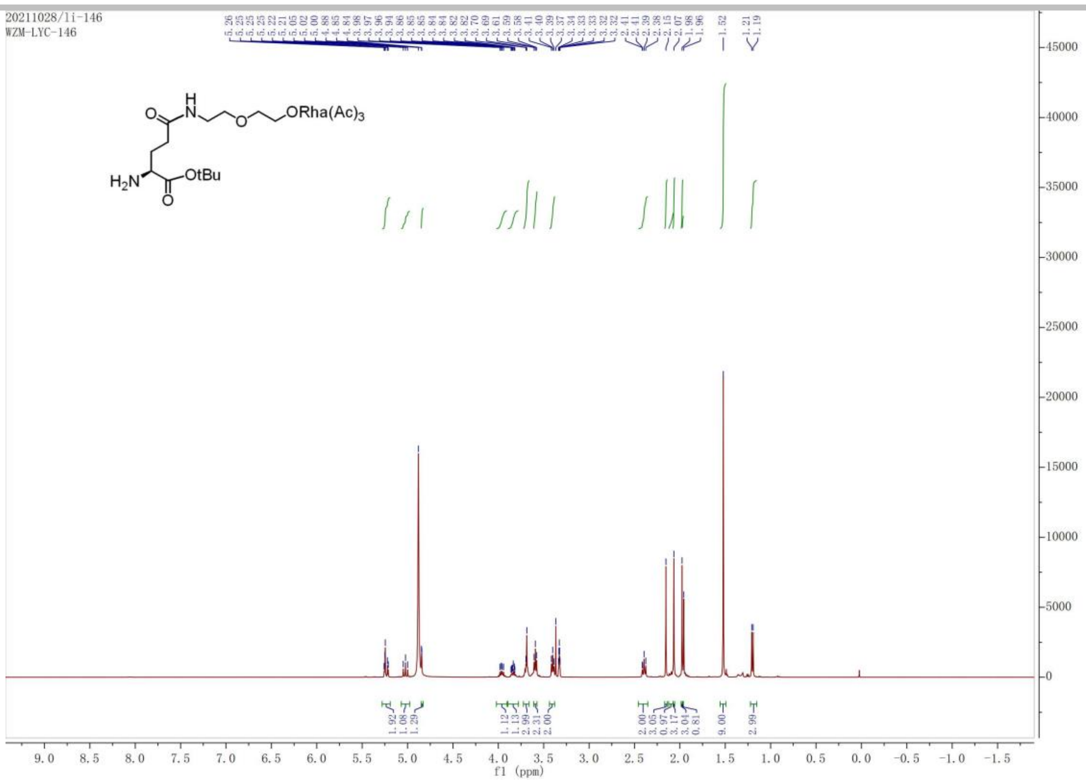

^{1}H-NMR spectrum of compound **8**

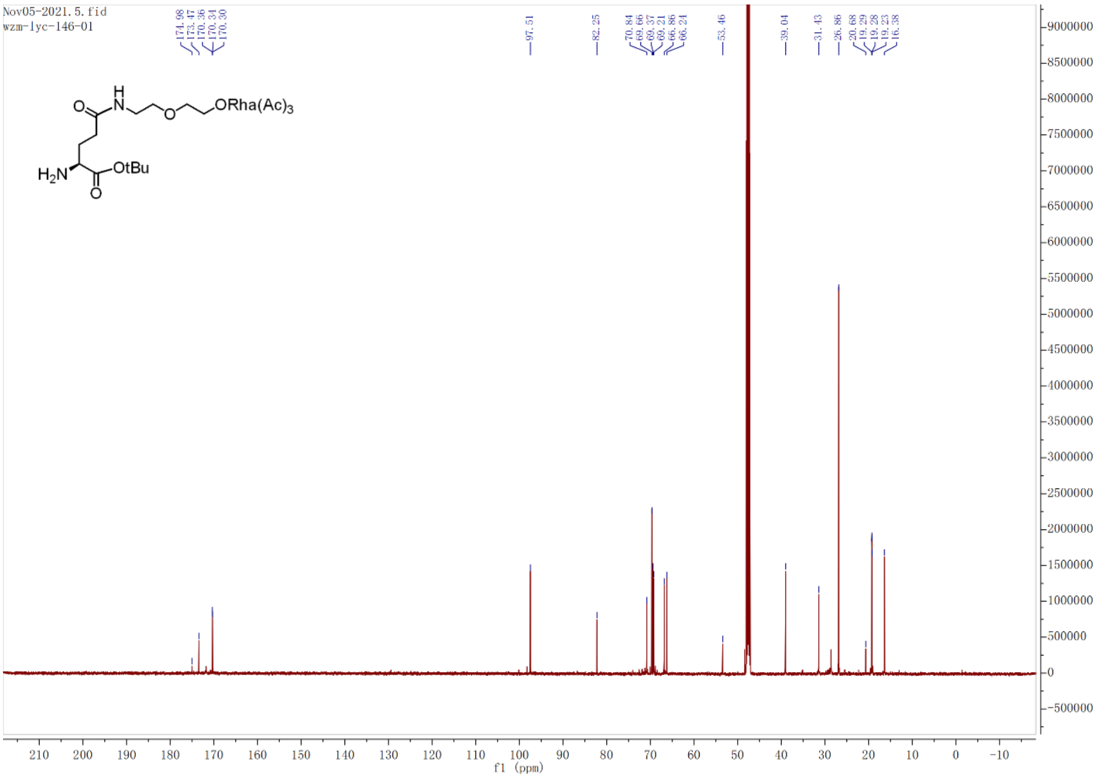

^{13}C-NMR spectrum of compound **8**

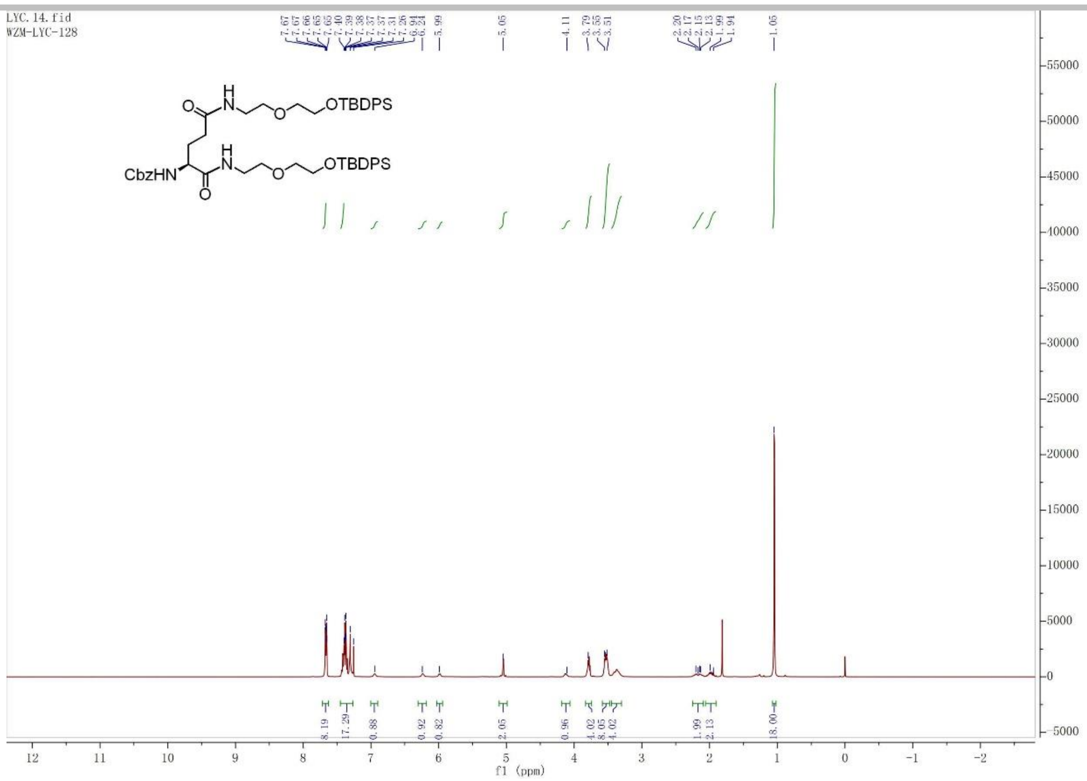

^{1}H-NMR spectrum of compound **S15**

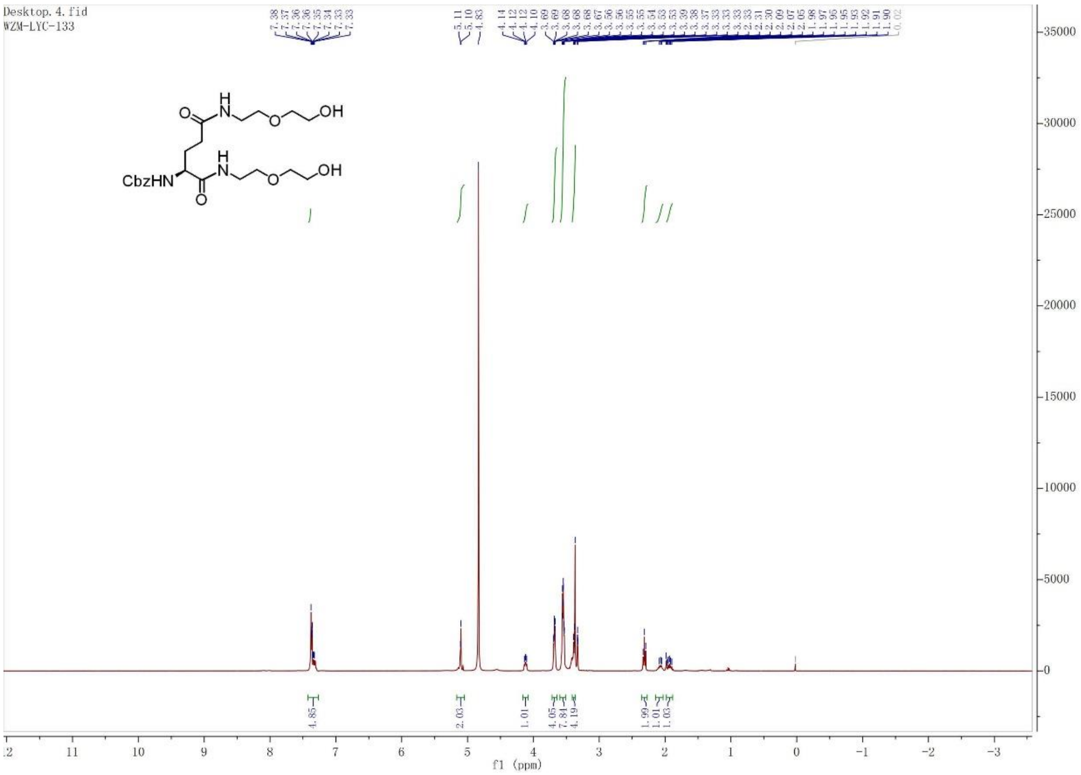

^{1}H-NMR spectrum of compound **S16**

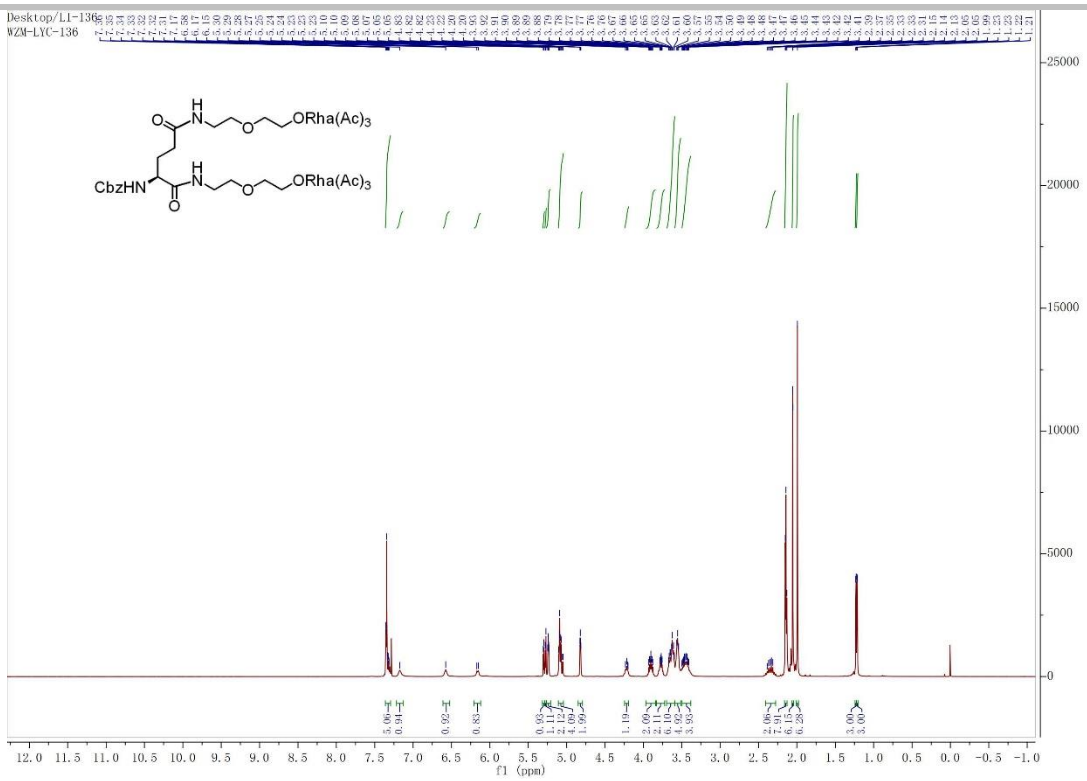

^{1}H-NMR spectrum of compound **S17**

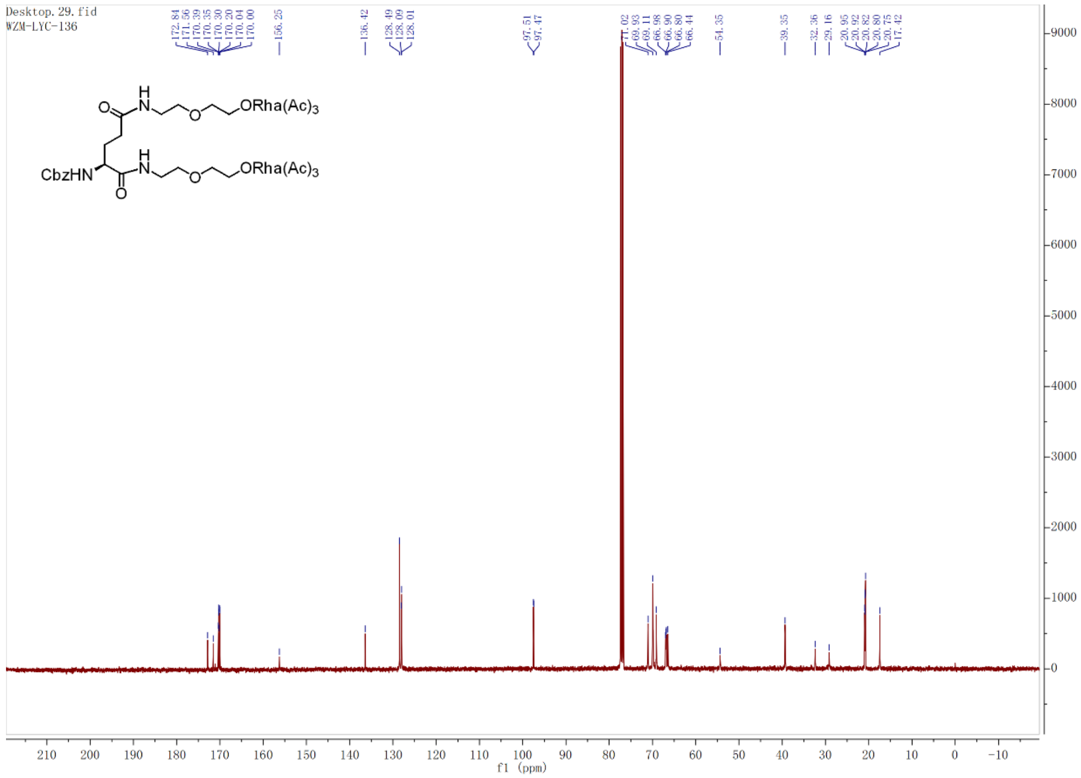

^{13}C-NMR spectrum of compound **S17**

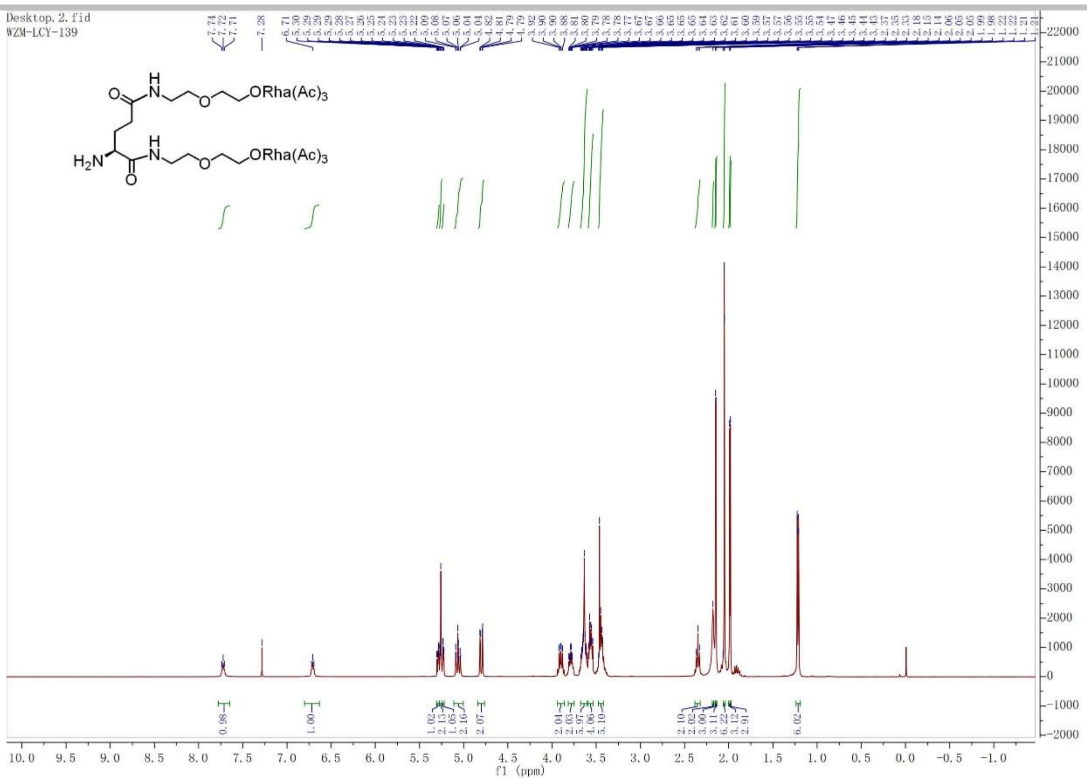

^{1}H-NMR spectrum of compound **9**

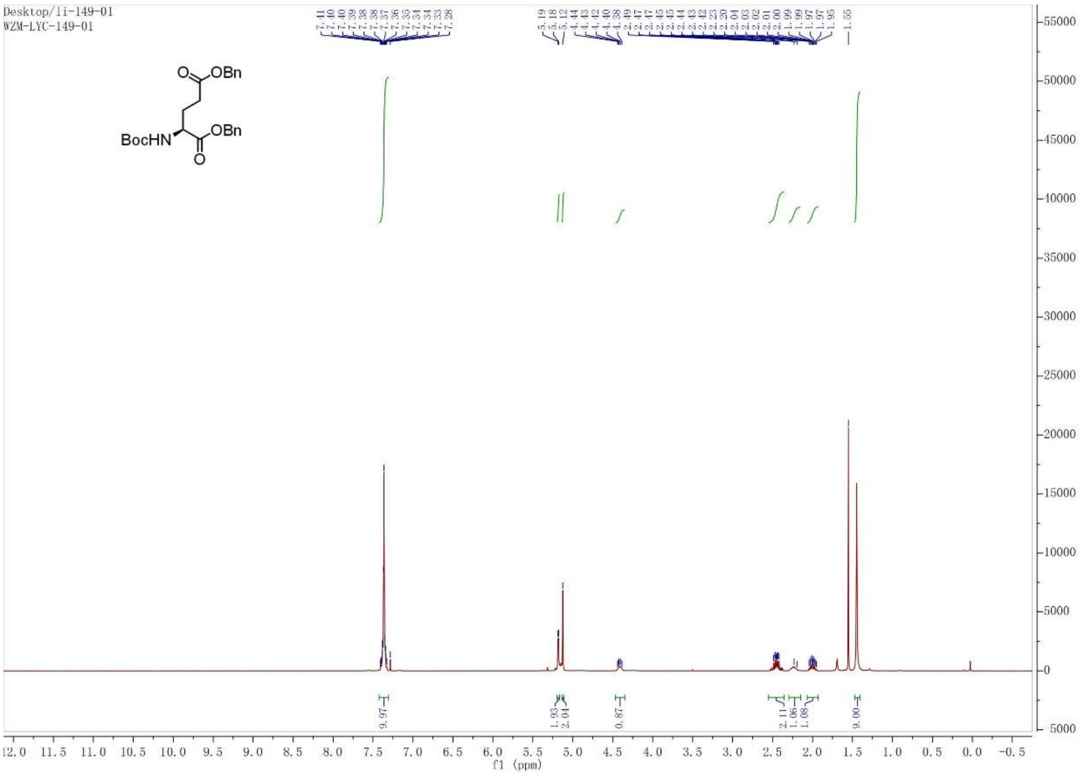

^{1}H-NMR spectrum of compound **S19**

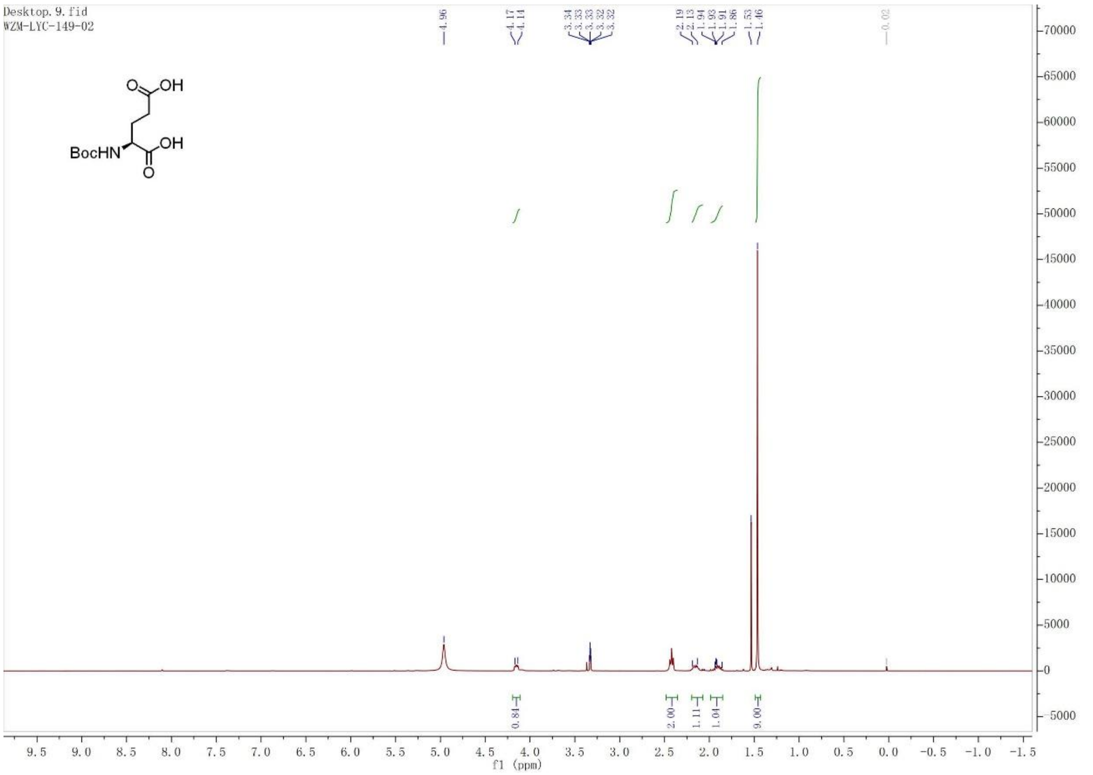

^{1}H-NMR spectrum of compound **S20**

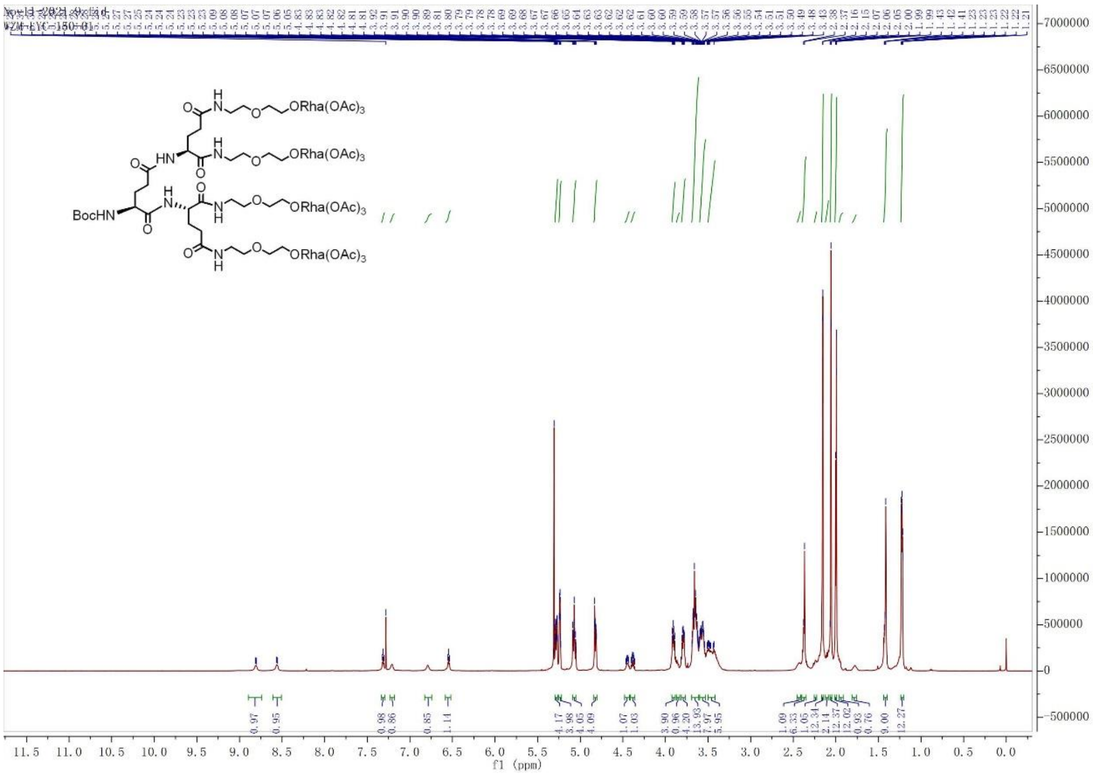

^{1}H-NMR spectrum of compound **S21**

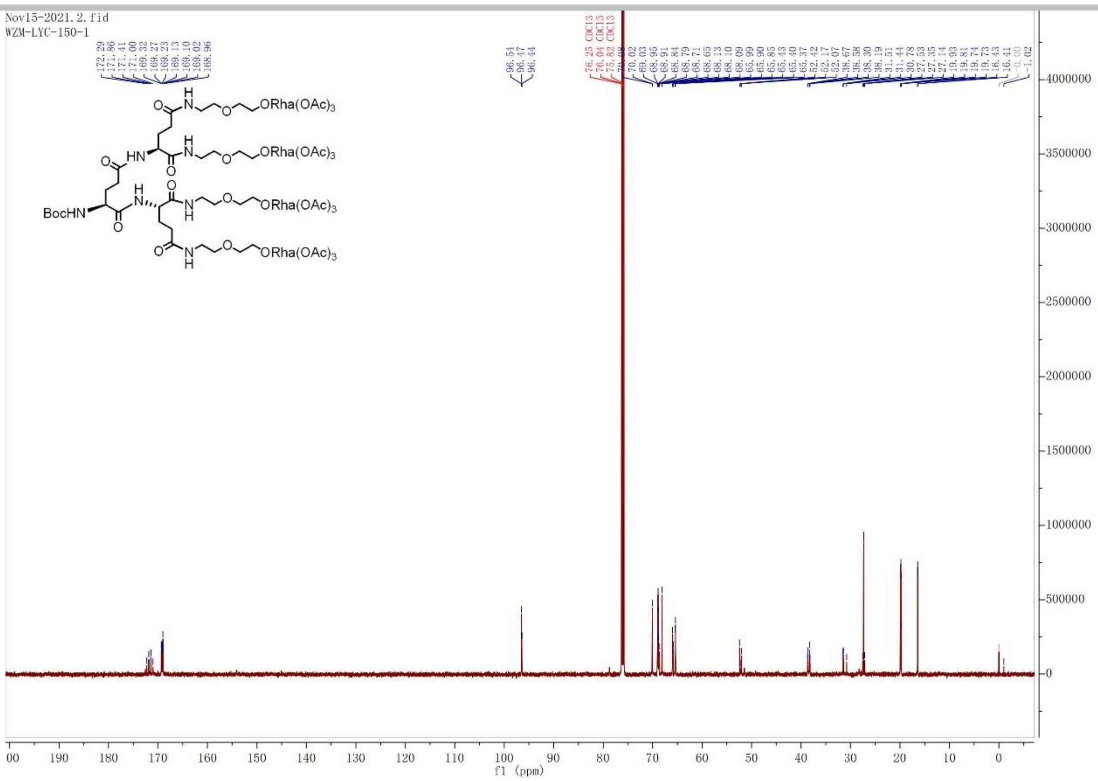

^{13}C-NMR spectrum of compound **S21**

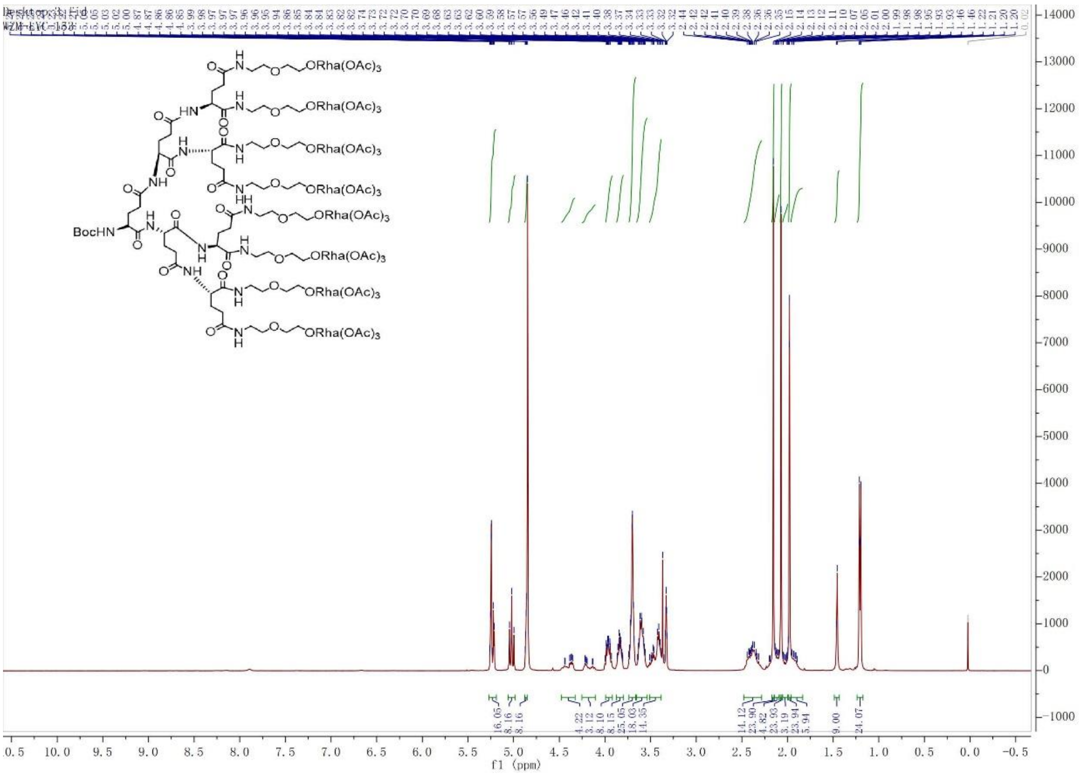

^{1}H-NMR spectrum of compound **S24**

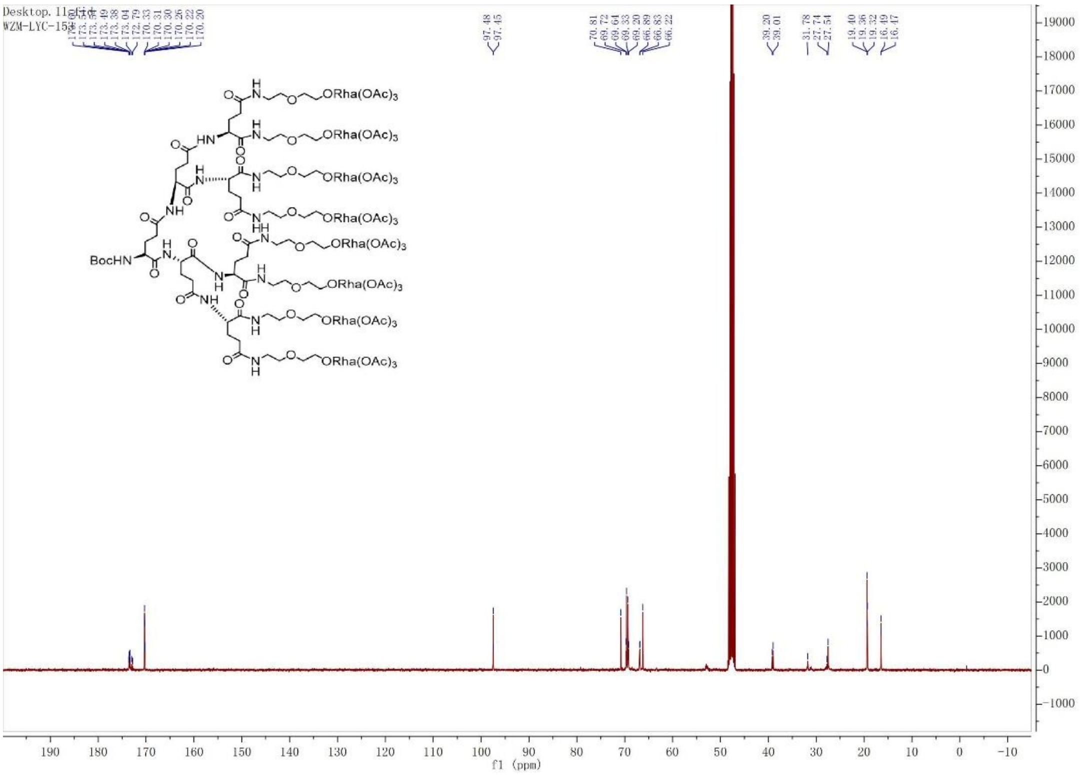

^{13}C-NMR spectrum of compound **S24**

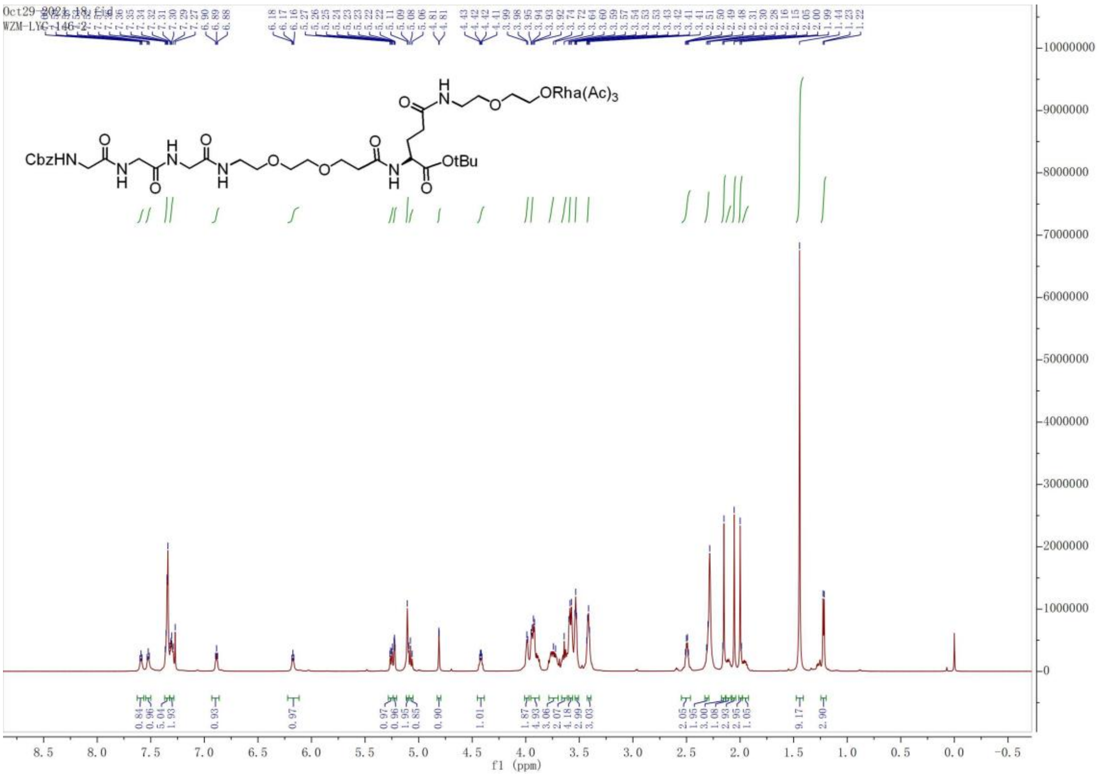

^{1}H-NMR spectrum of compound **S25**

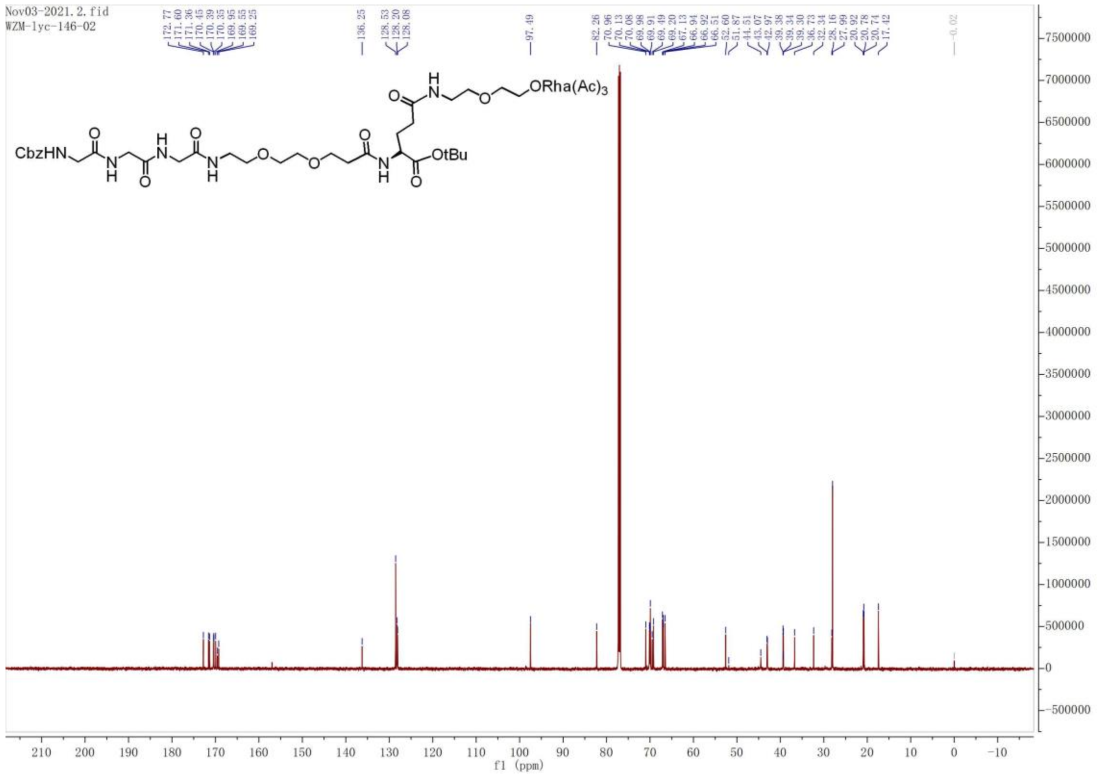

^{13}C-NMR spectrum of compound **S25**

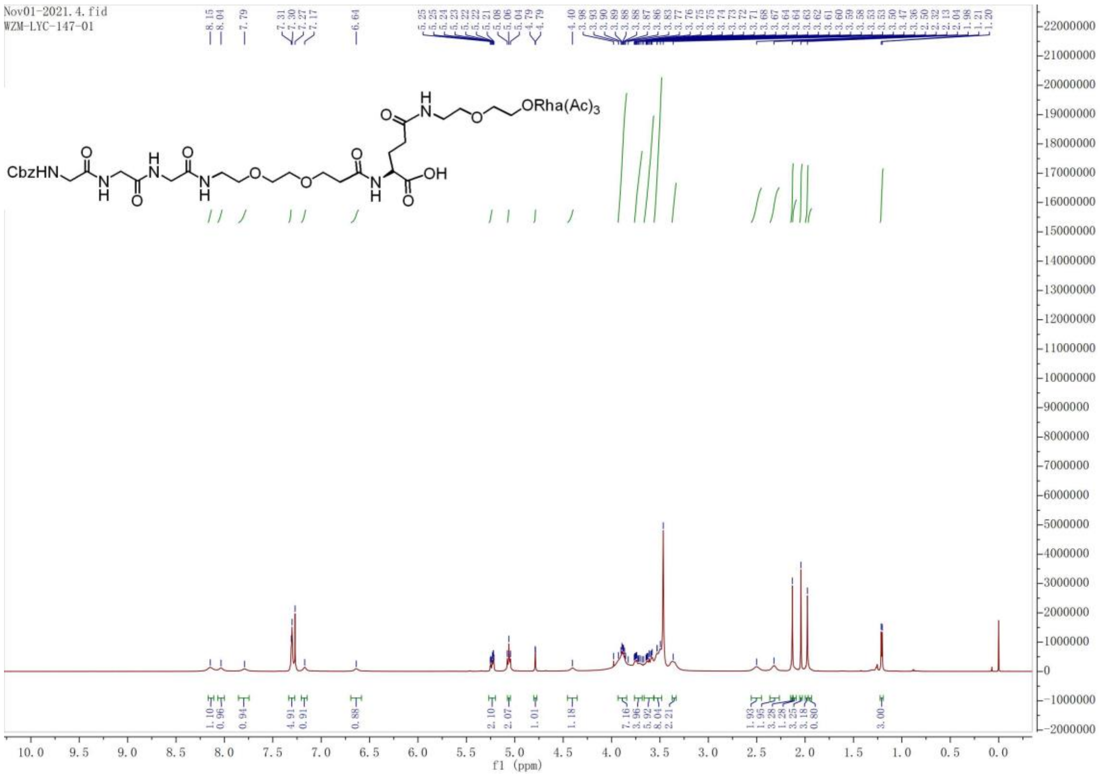

^{1}H-NMR spectrum of compound **S26**

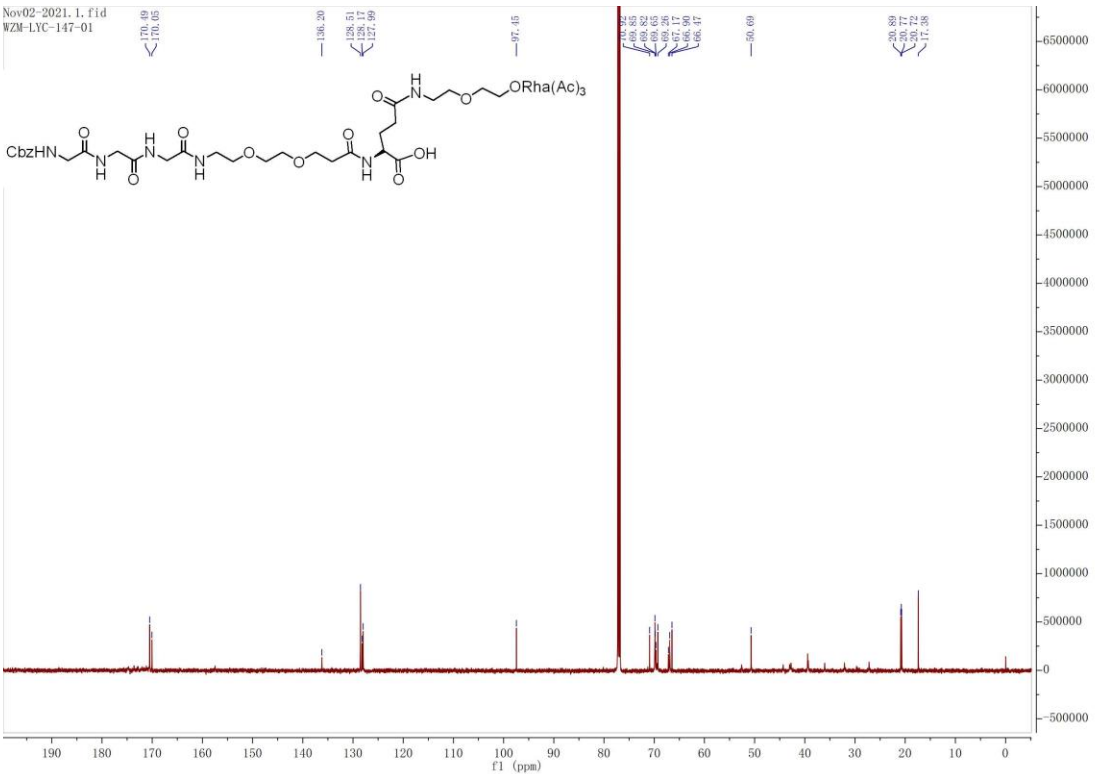

^{13}C-NMR spectrum of compound **S26**

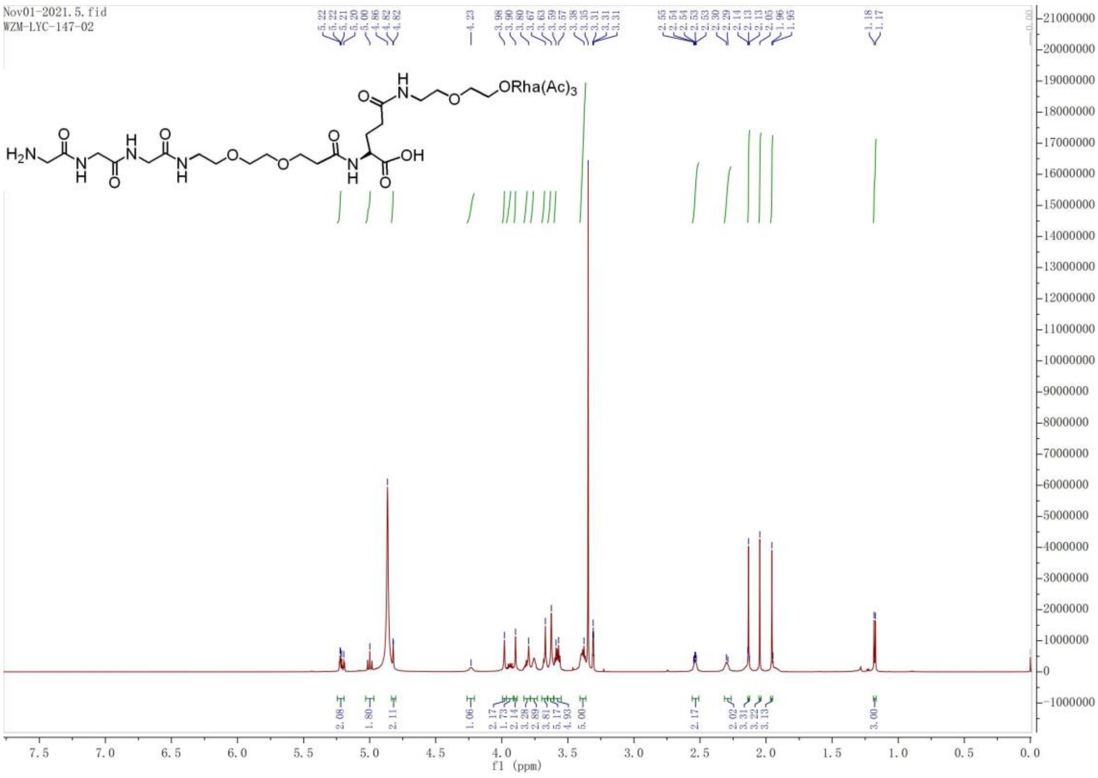

^{1}H-NMR spectrum of compound **S27**

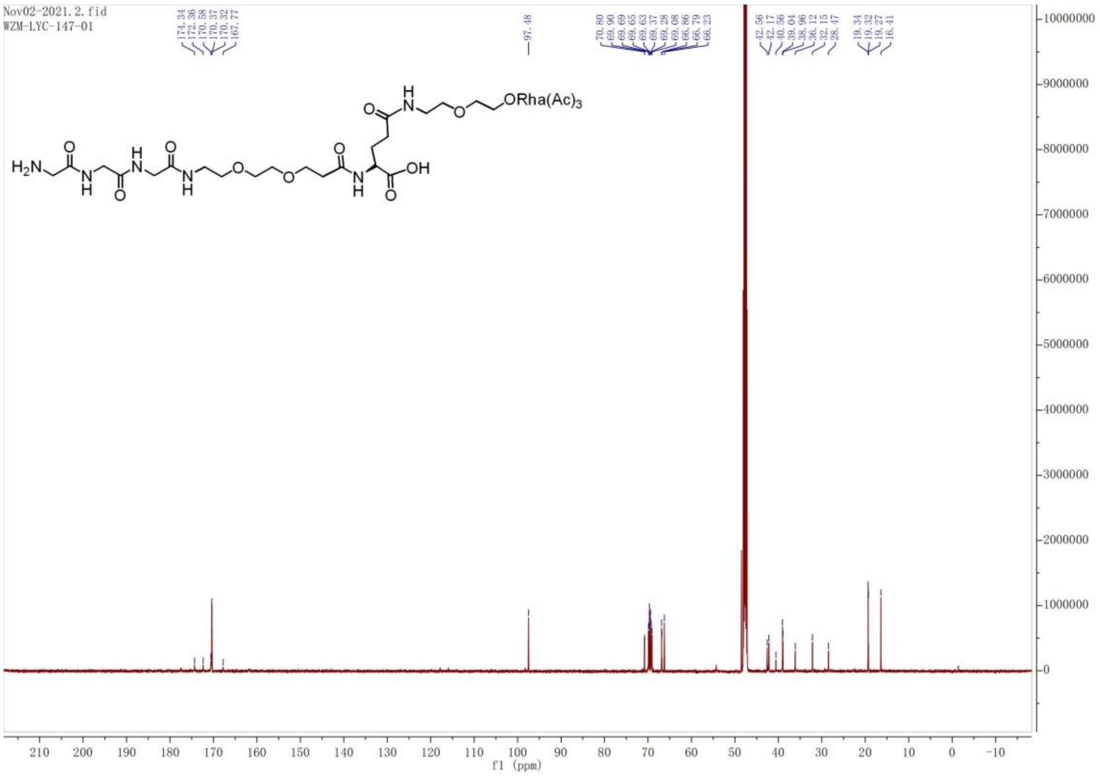

^{13}C-NMR spectrum of compound **S27**

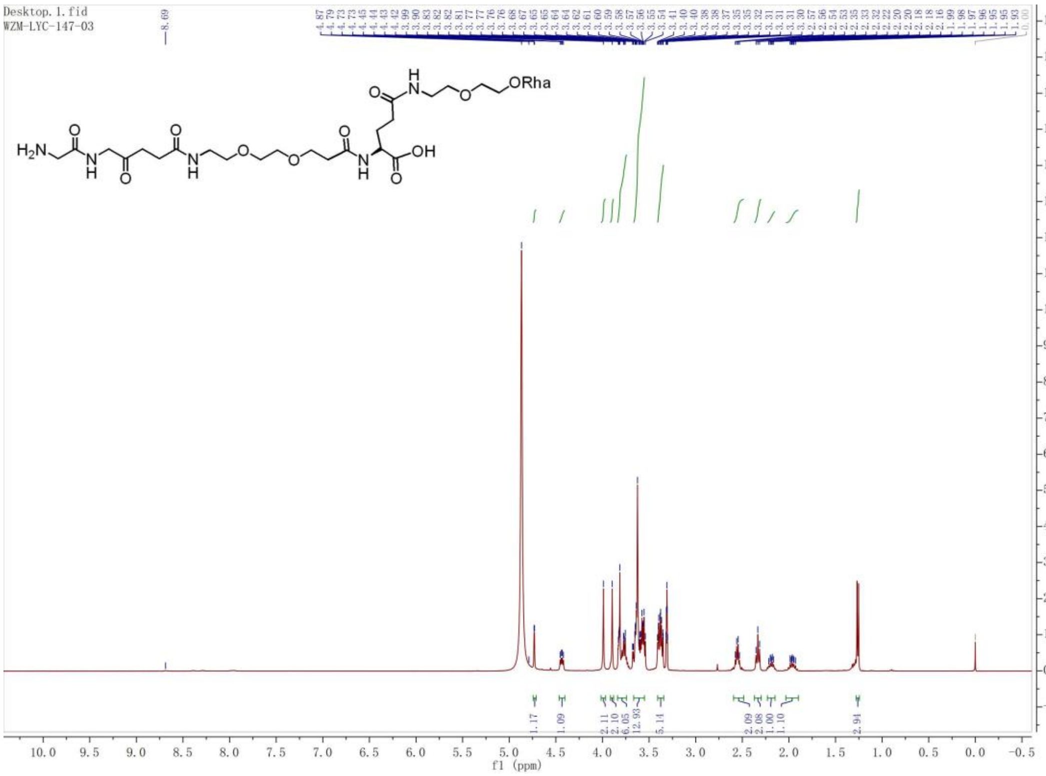

^{1}H-NMR spectrum of compound **1**

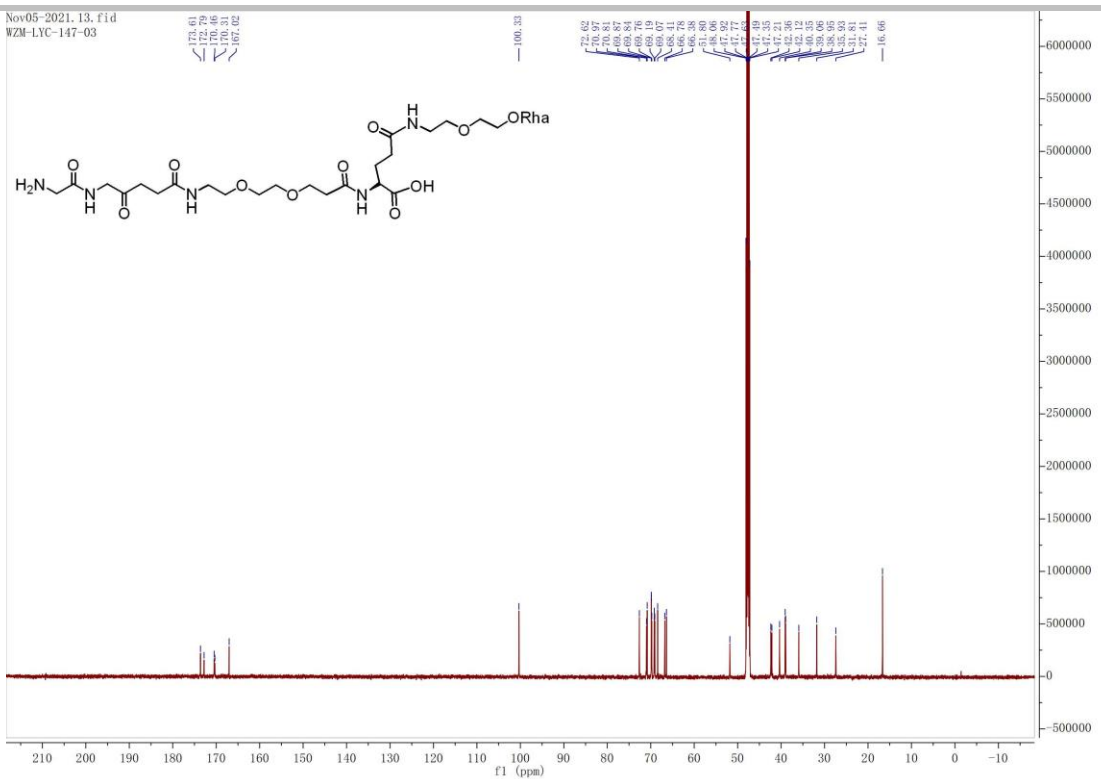

^{13}C-NMR spectrum of compound **1**

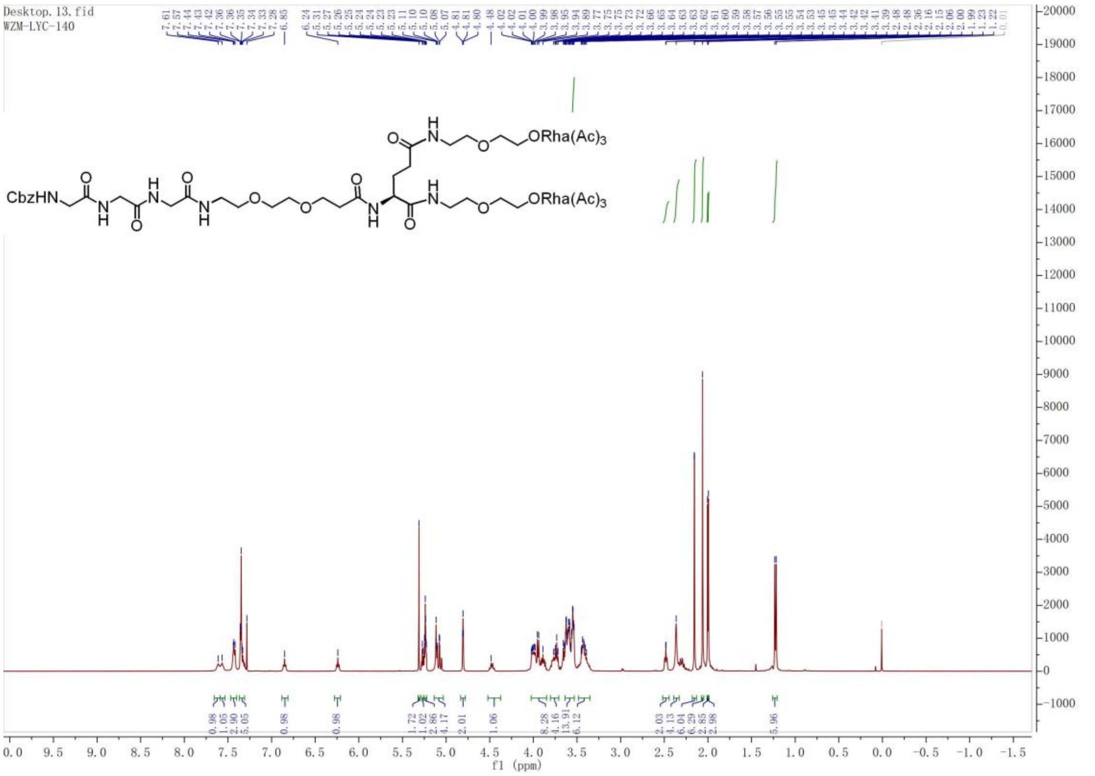

^{1}H-NMR spectrum of compound **S28**

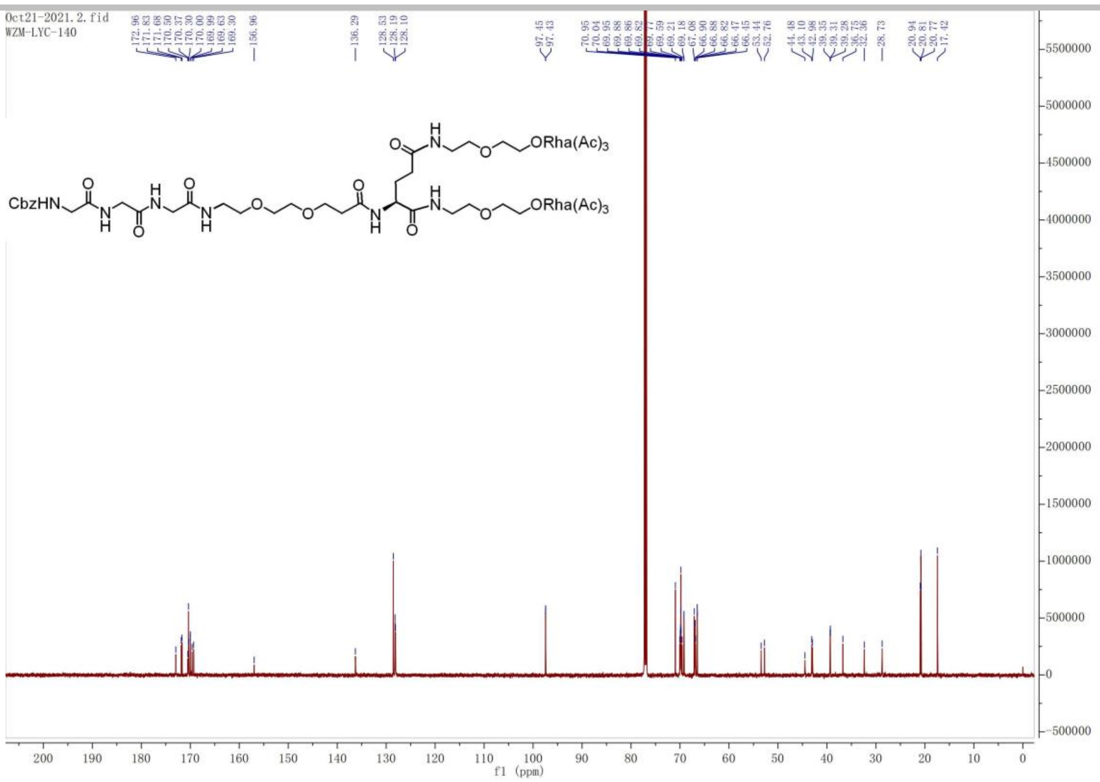

^{13}C-NMR spectrum of compound **S28**

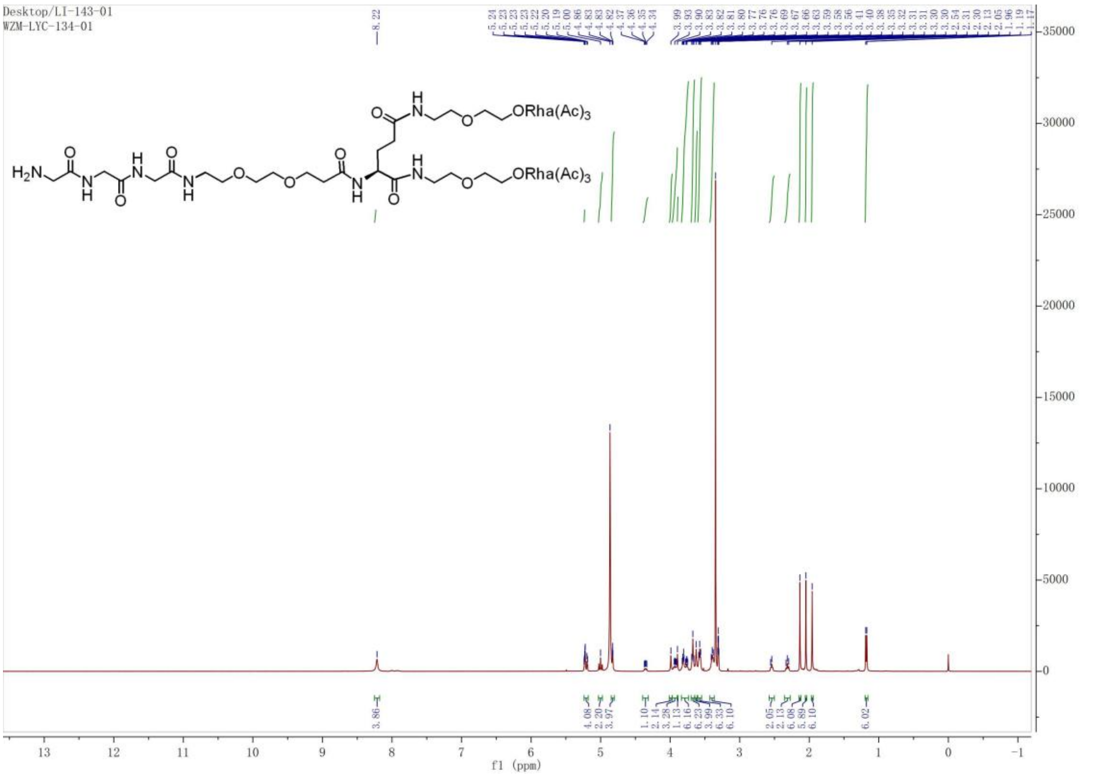

^{1}H-NMR spectrum of compound **S29**

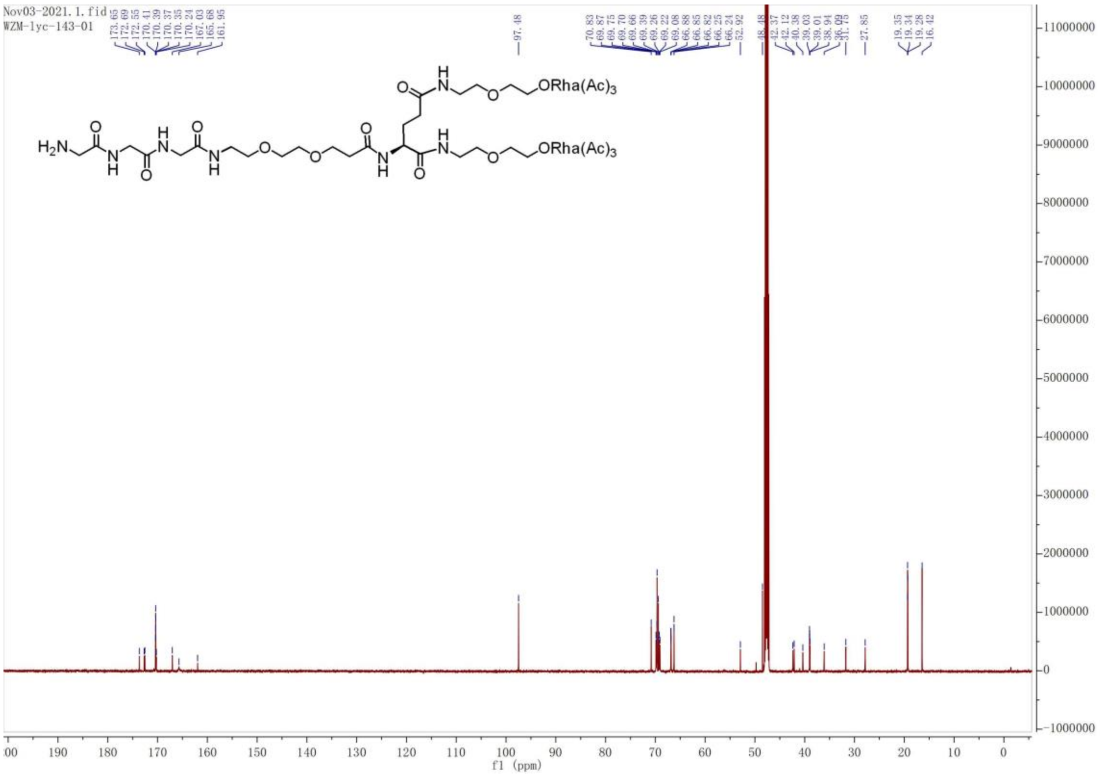

^{13}C-NMR spectrum of compound **S29**

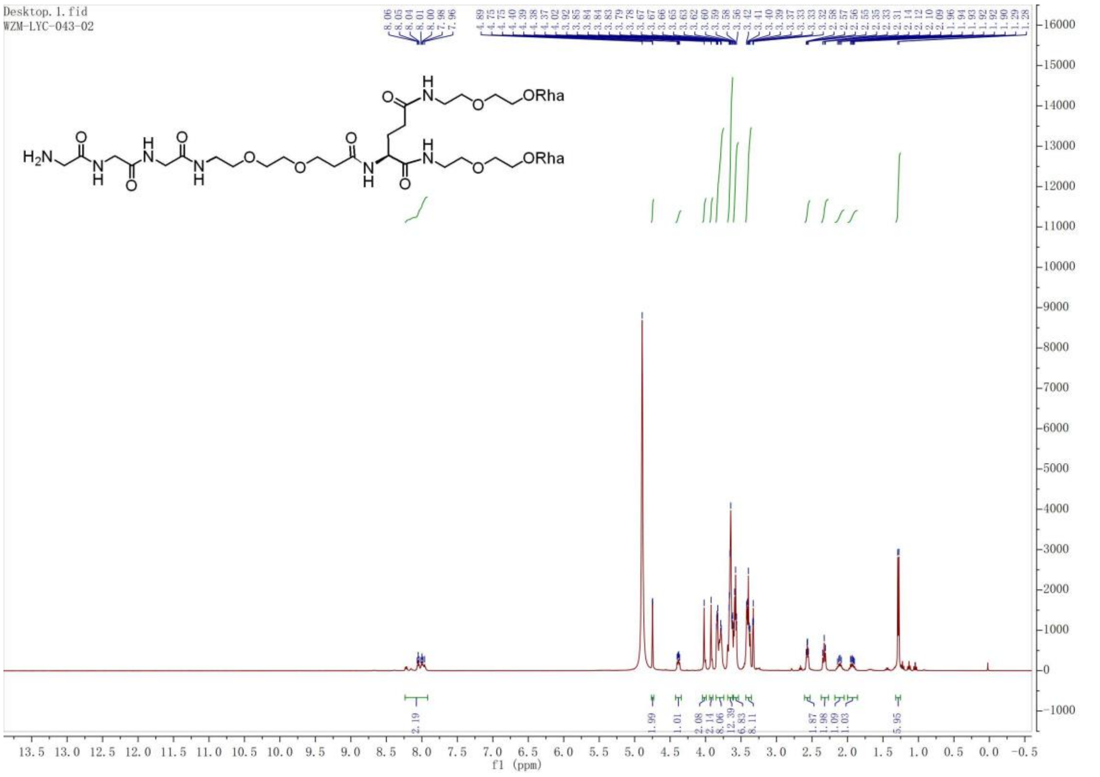

^{1}H-NMR spectrum of compound **2**

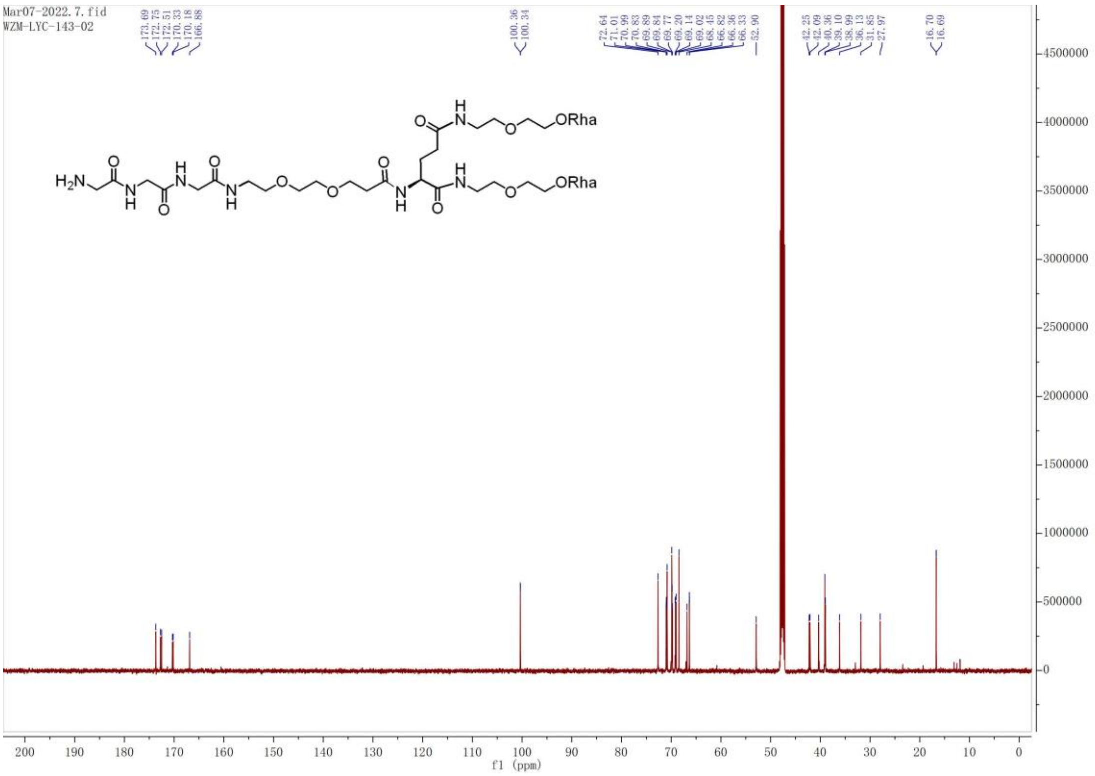

^{13}C-NMR spectrum of compound **2**

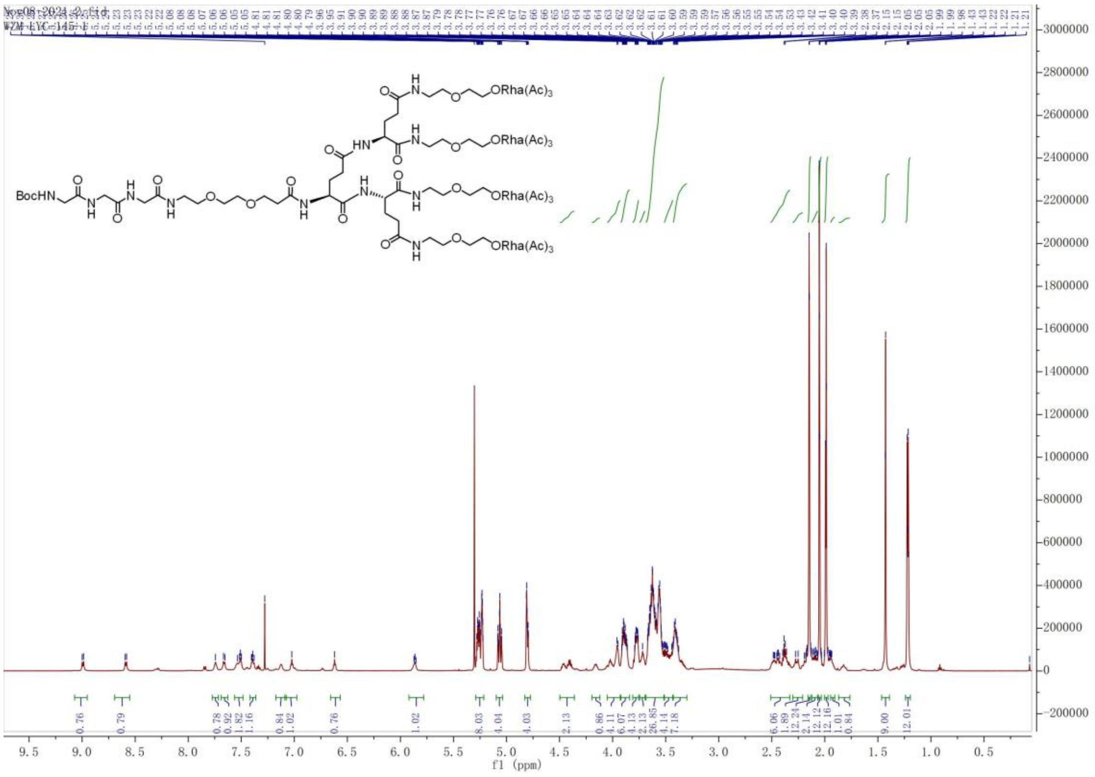

^{1}H-NMR spectrum of compound **S30**

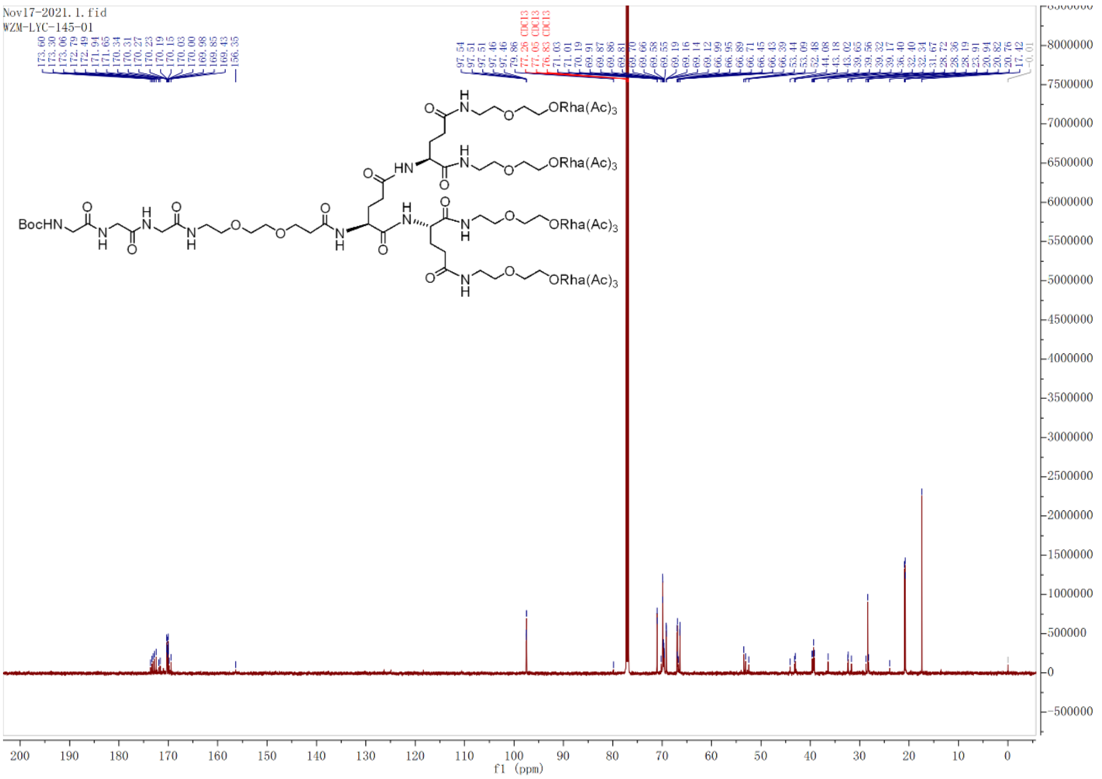

^{13}C-NMR spectrum of compound **S30**

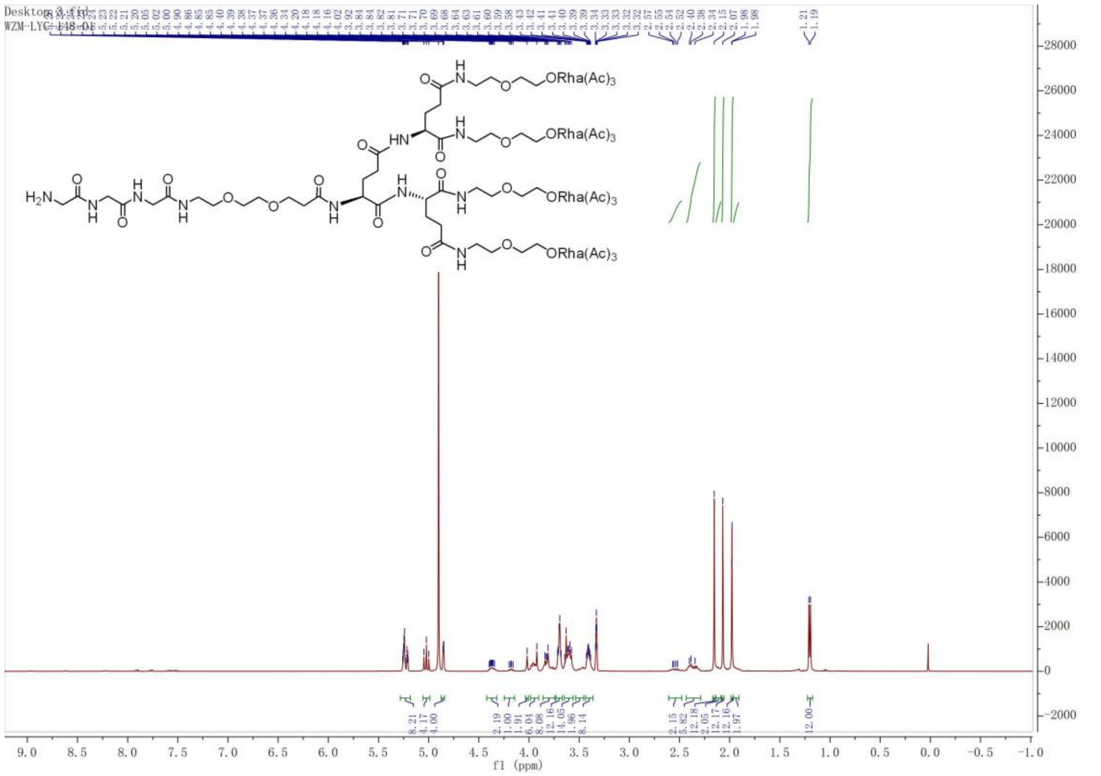

^{1}H-NMR spectrum of compound **S31**

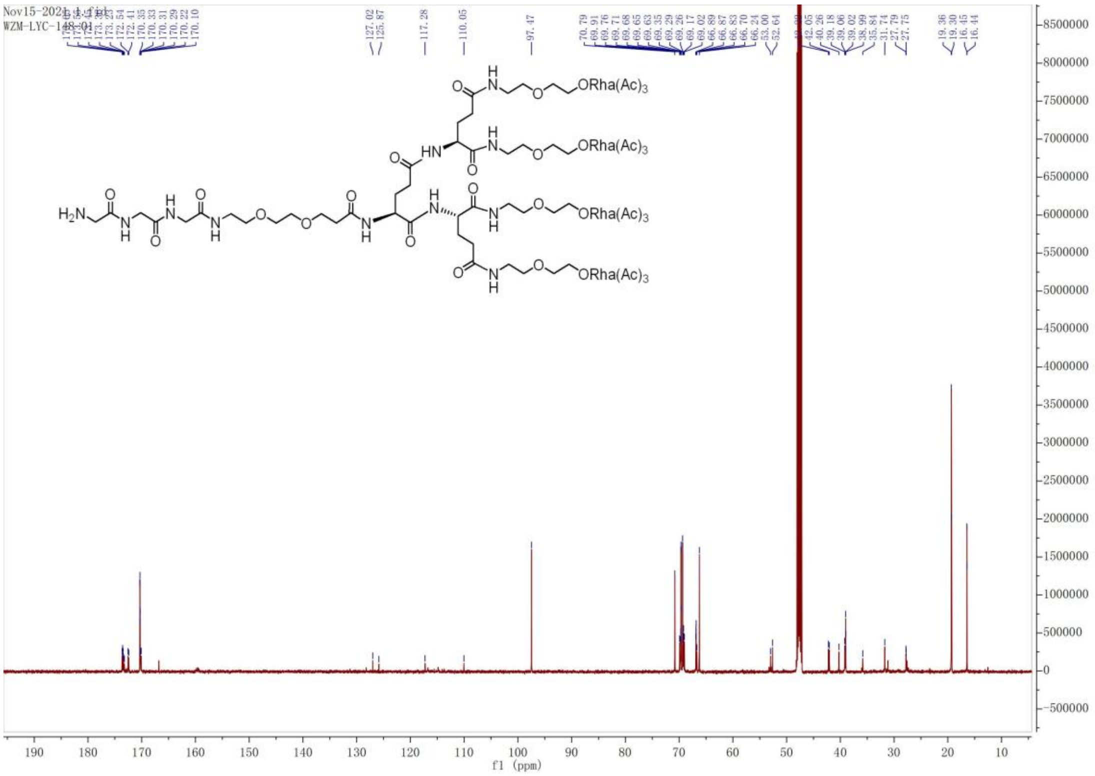

^{13}C-NMR spectrum of compound **S31**

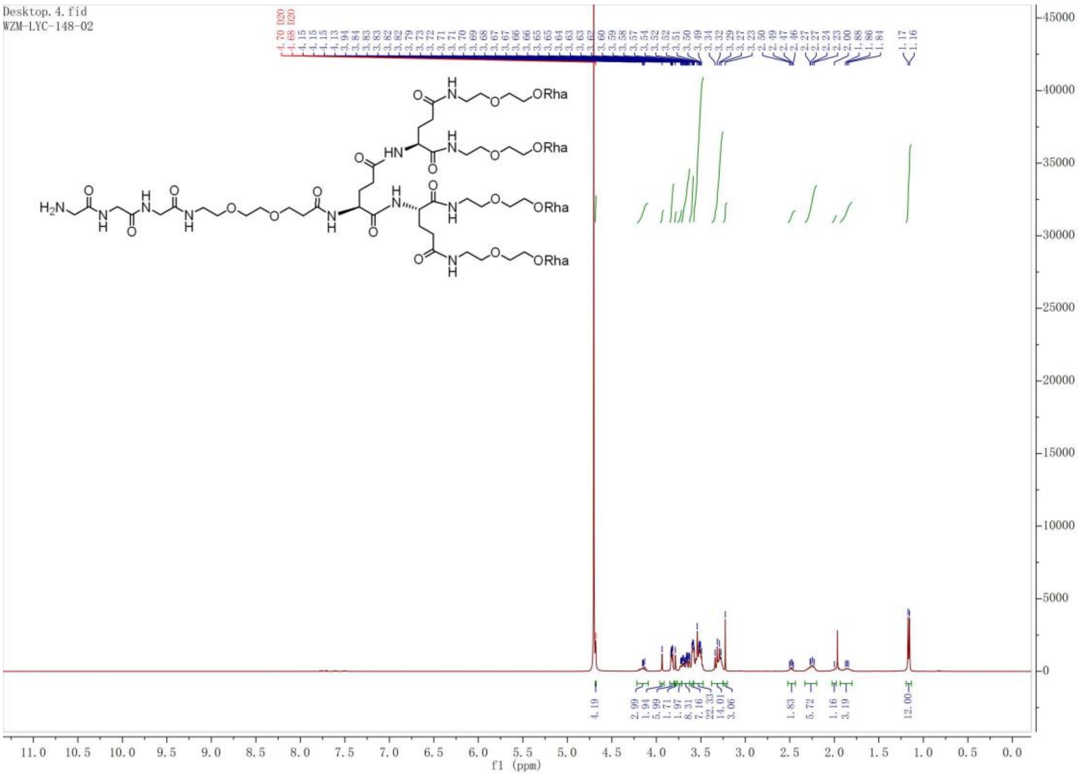

^{1}H-NMR spectrum of compound **3**

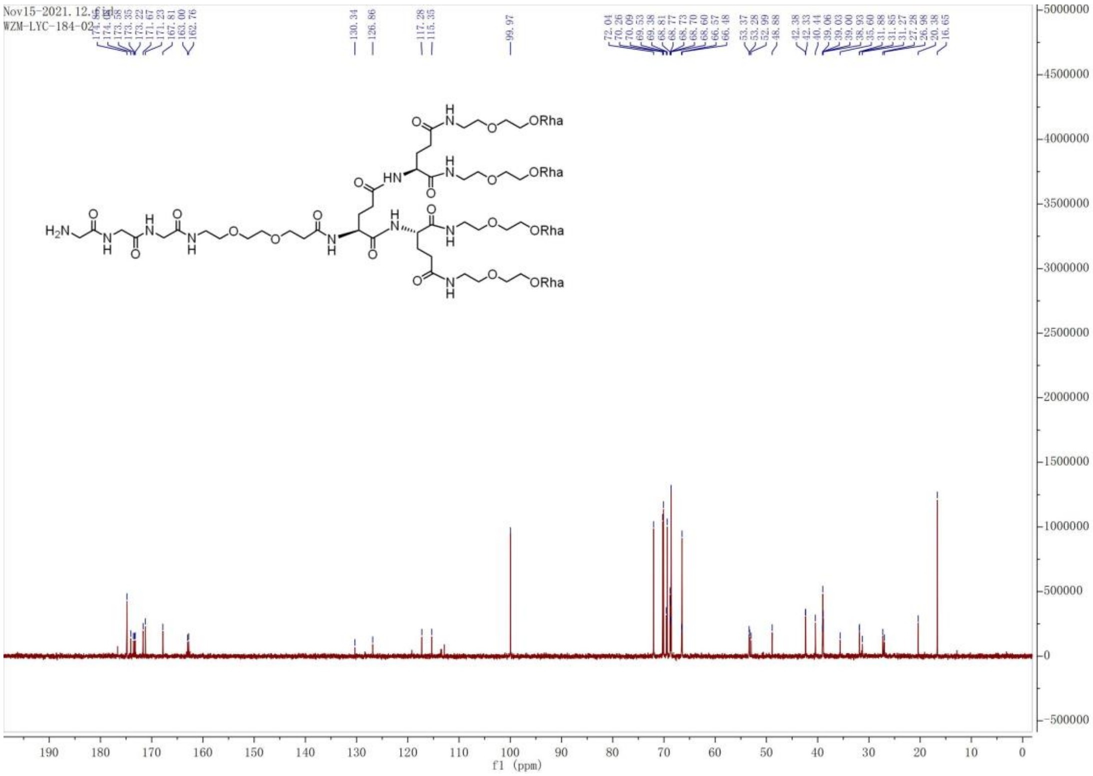

^{13}C-NMR spectrum of compound **3**

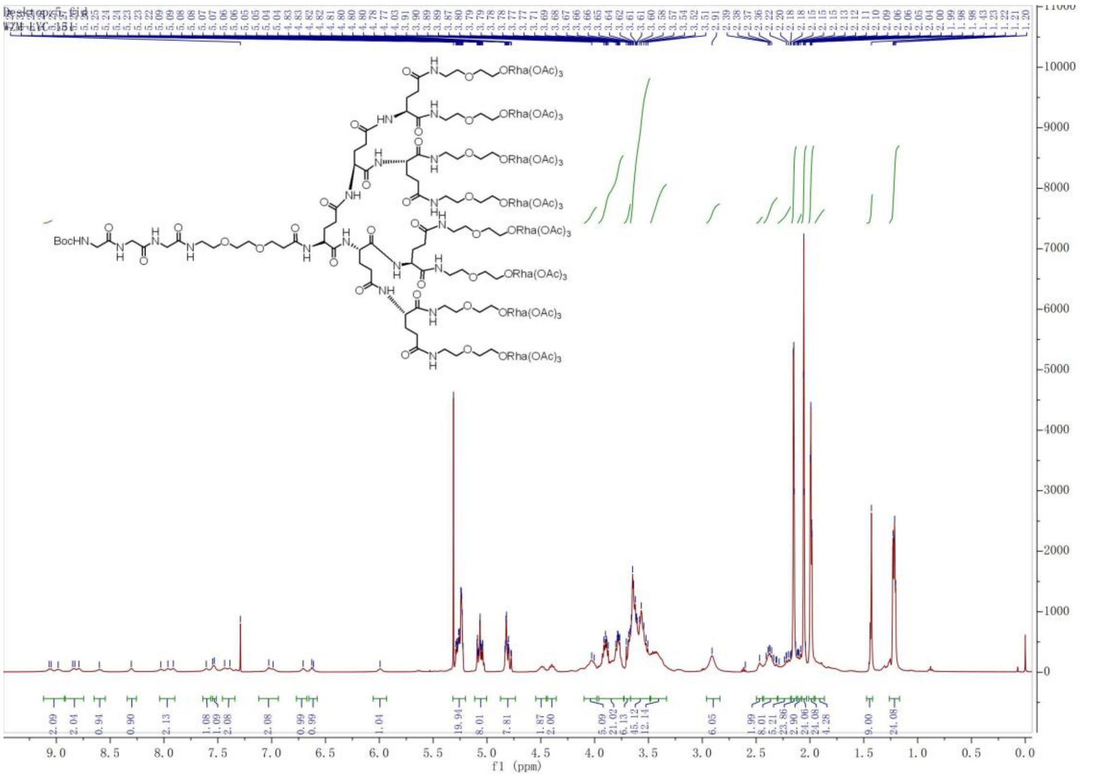

^{1}H-NMR spectrum of compound **32**

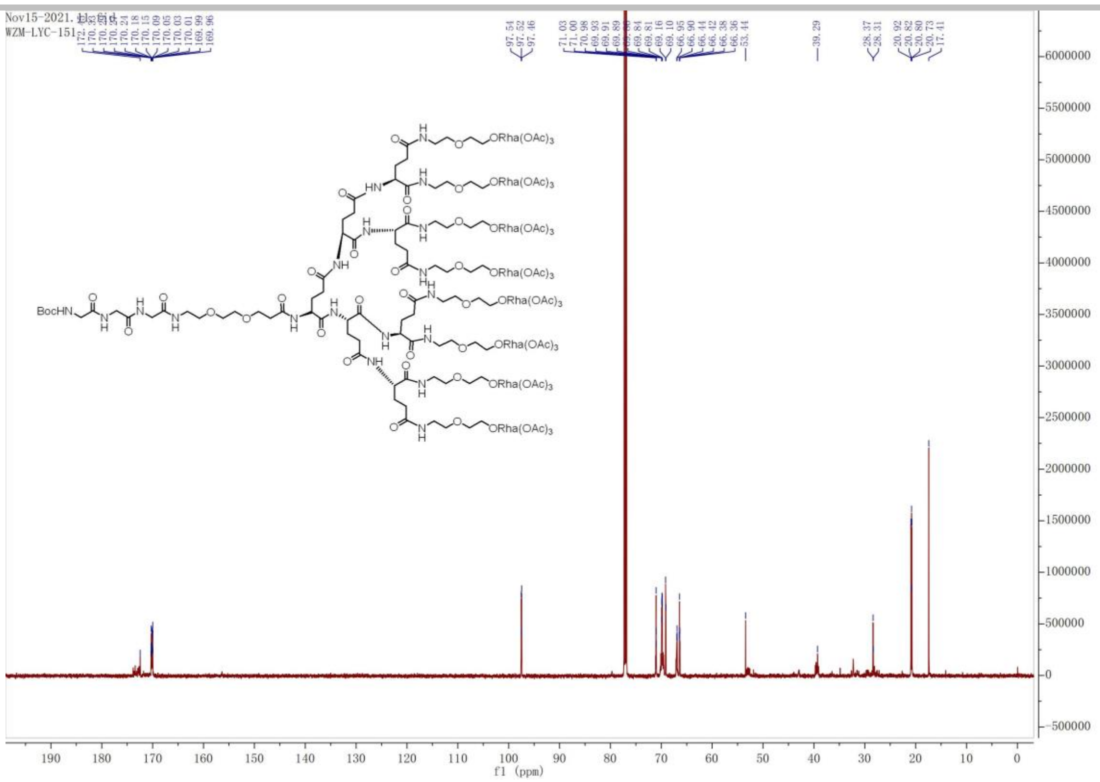

^{13}C-NMR spectrum of compound **S32**

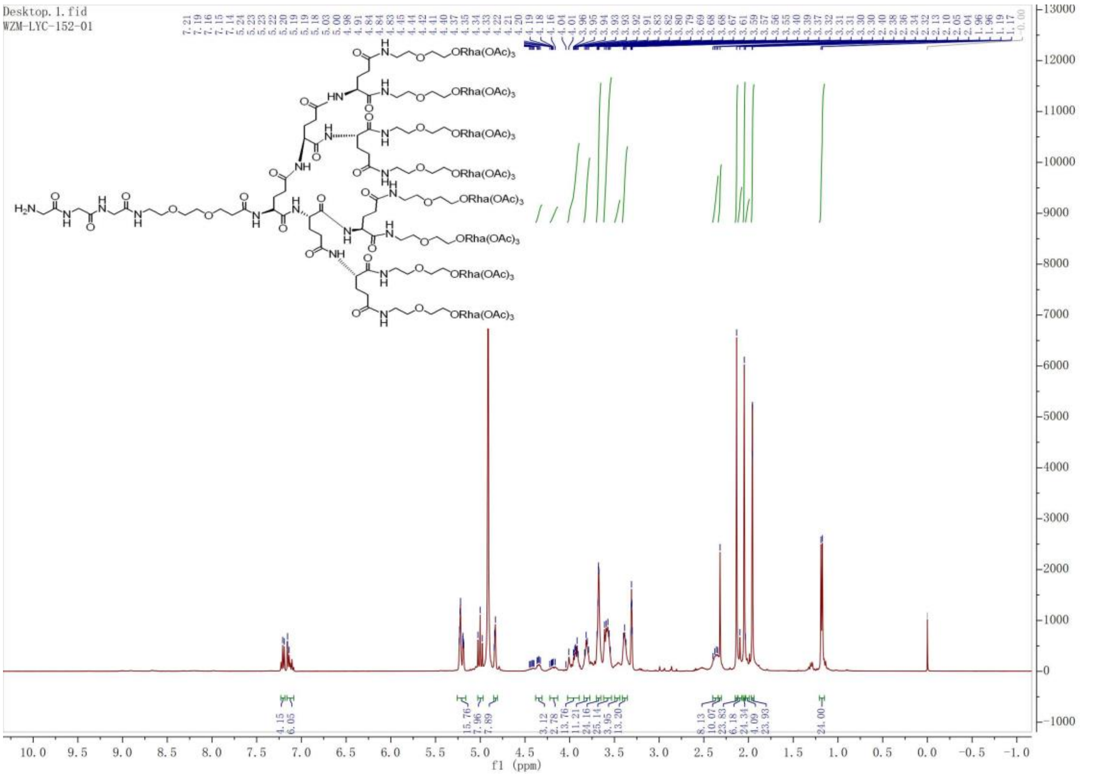

^{1}H-NMR spectrum of compound **33**

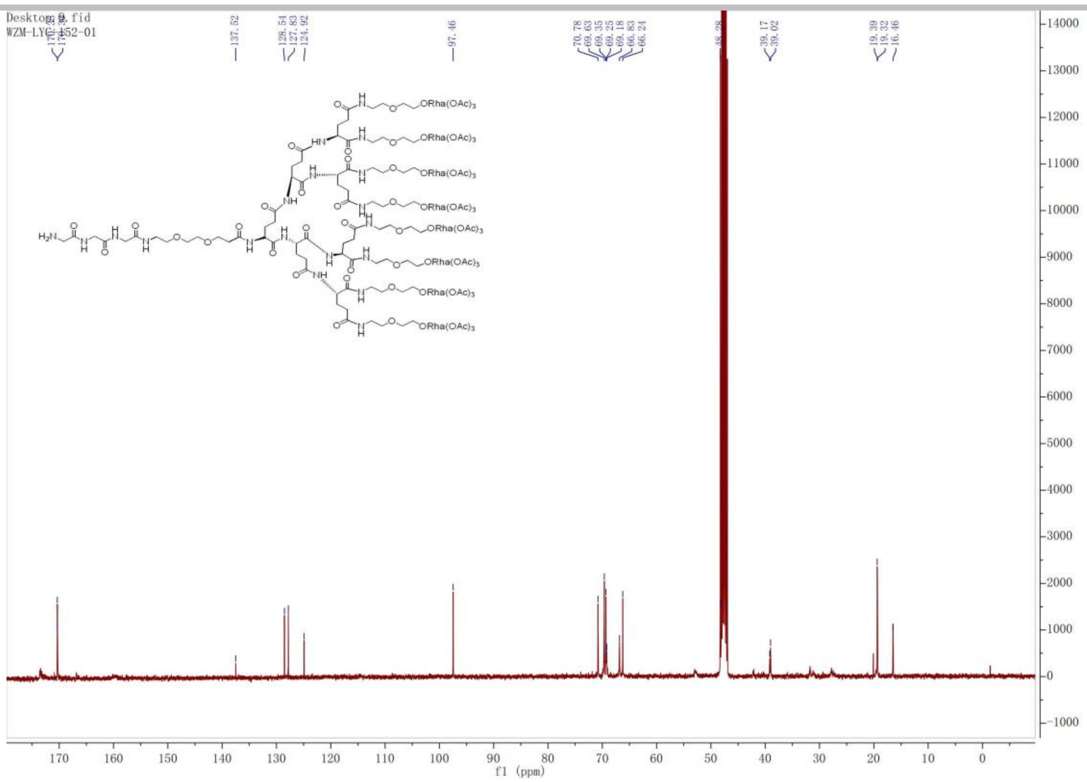

^{13}C-NMR spectrum of compound **S33**

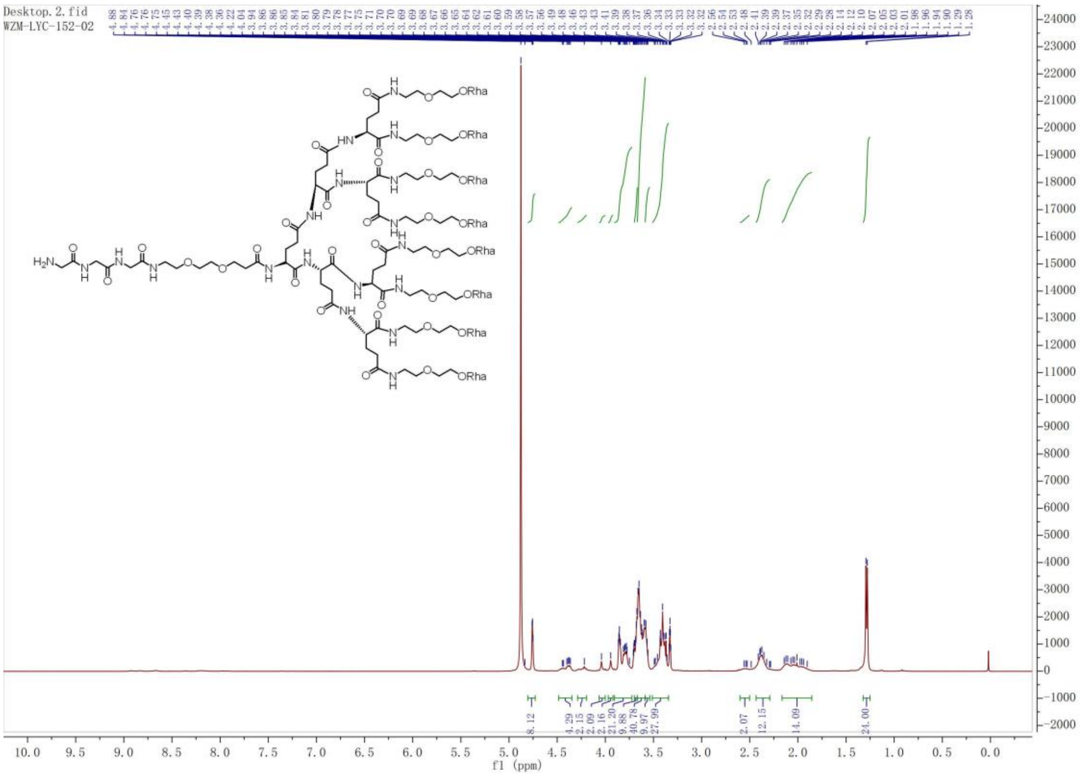

^{1}H-NMR spectrum of compound **4**

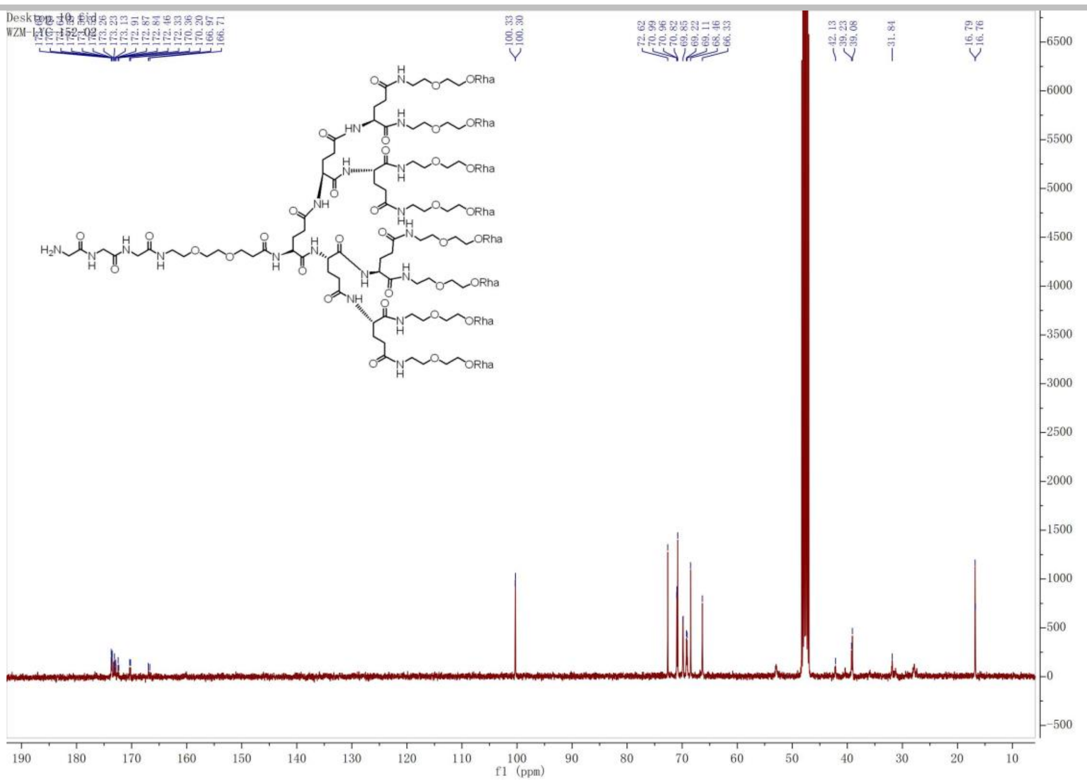

^{13}C-NMR spectrum of compound **4**

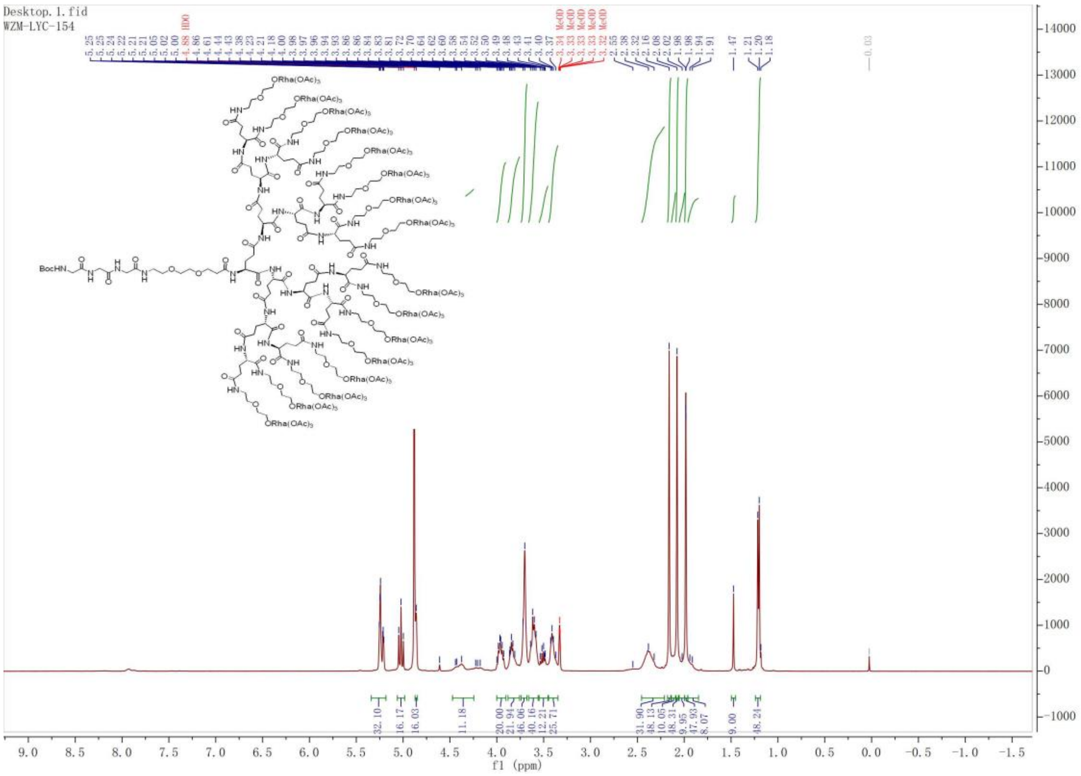

^{1}H-NMR spectrum of compound **S34**

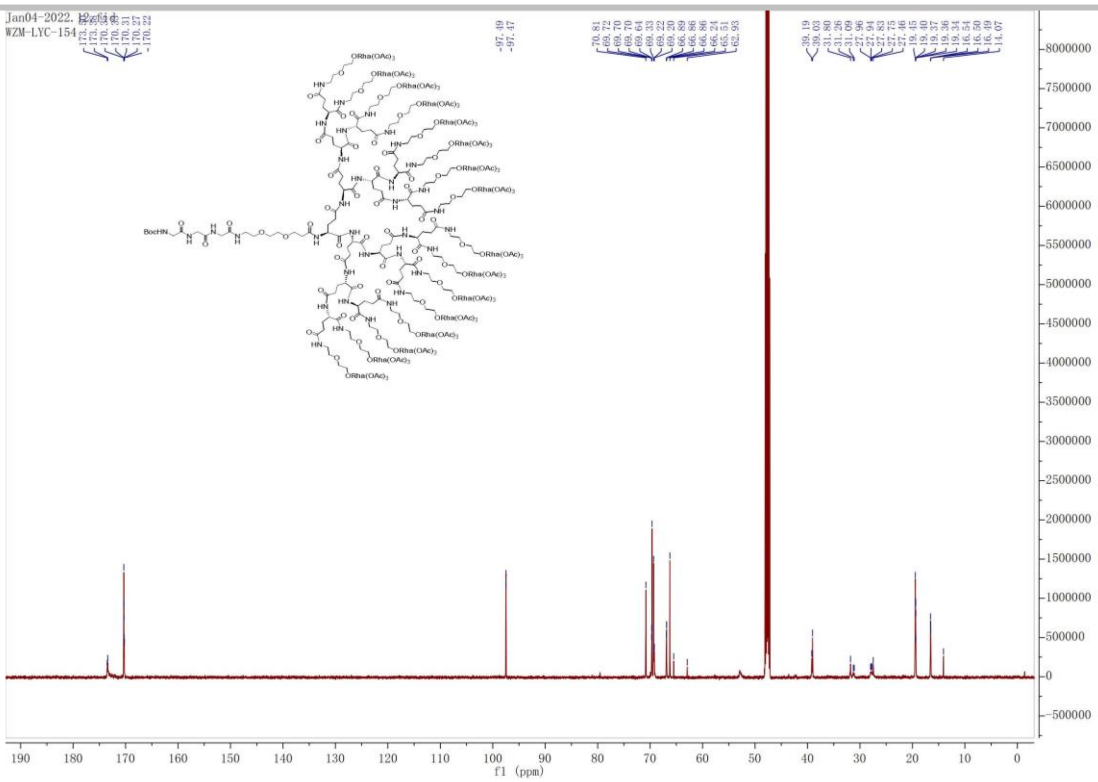

^{13}C-NMR spectrum of compound **S34**

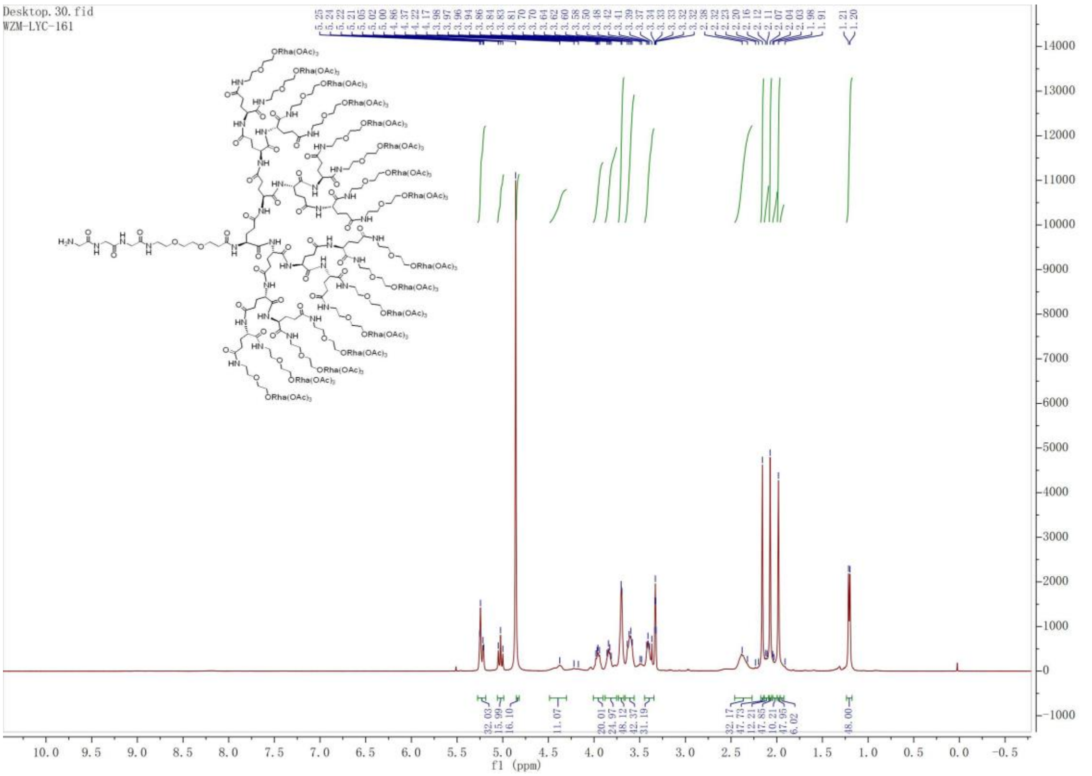

^{1}H-NMR spectrum of compound **S35**

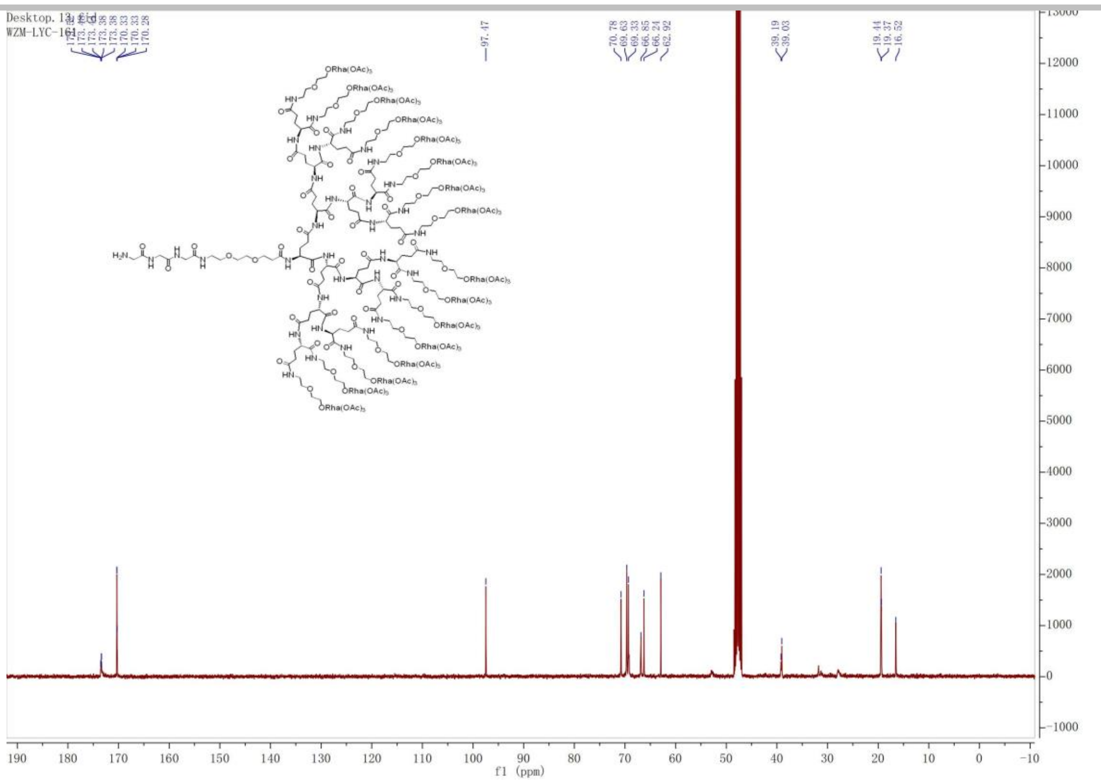

^{13}C-NMR spectrum of compound **S35**

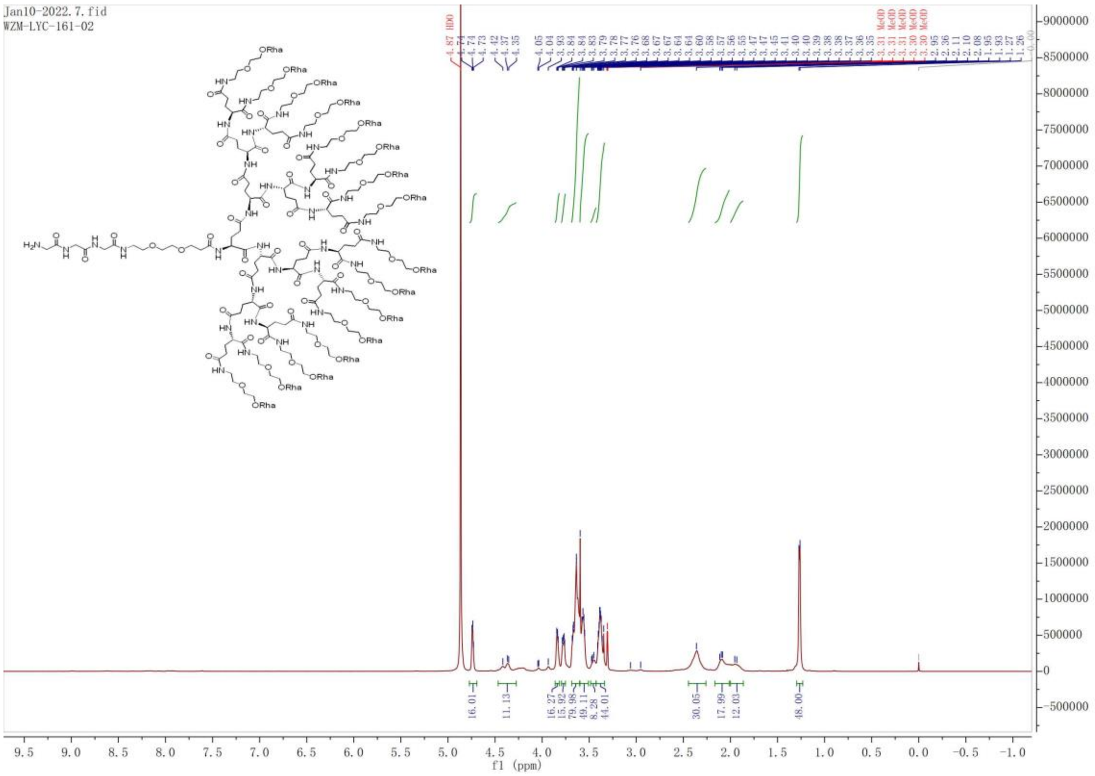

^{1}H-NMR spectrum of compound **5**

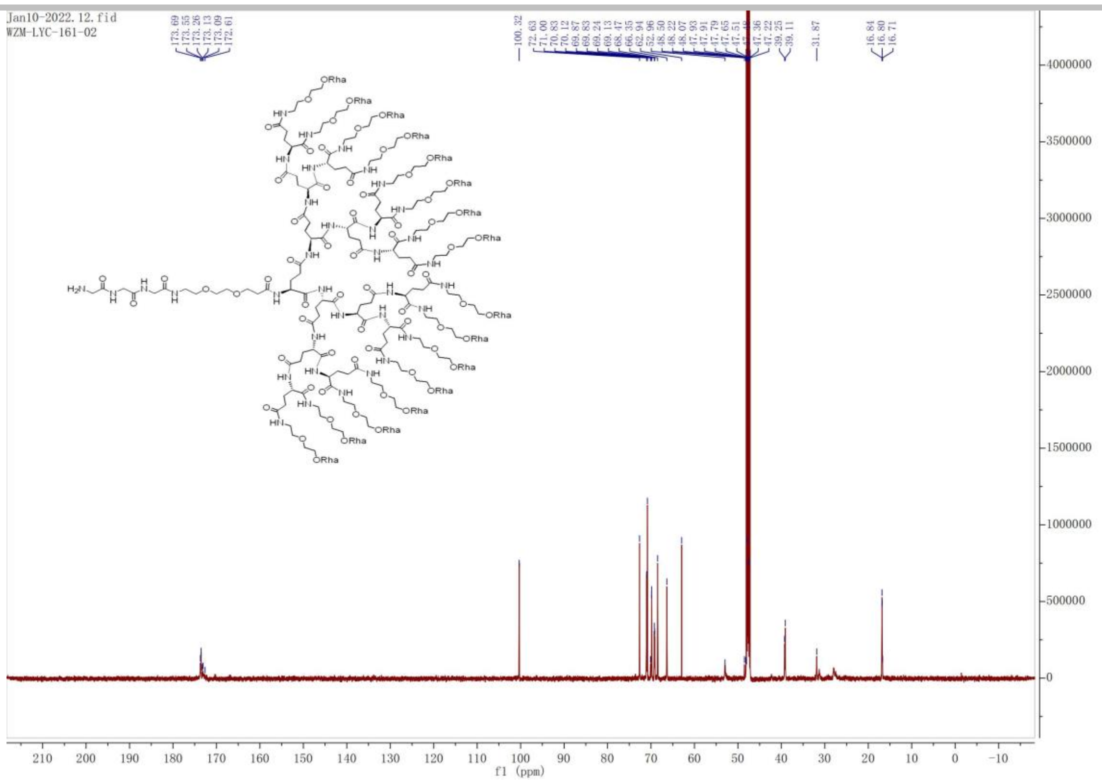

^{13}C-NMR spectrum of compound **5**

References

1. O. Plettenburg, V. Bodmer-Narkevitch and C. H. Wong, *J Org Chem*, 2002, **67**, 4559-4564.
2. P. V. Chang, D. H. Dube, E. M. Sletten and C. R. Bertozzi, *J Am Chem Soc*, 2010, **132**, 9516-9518.
3. K. Zhou, H. Hong, H. Lin, L. Gong, D. Li, J. Shi, Z. Zhou, F. Xu and Z. Wu, *J Med Chem*, 2022, **65**, 323-332.

Author Contributions

Z.W. conceived the modified strategy and supervised the project. Z.W., L.C. and H.H. designed the experiments and wrote a draft of the manuscript. L.C., H.L., L.G., J.Z., and Z.W. performed the experiments. L.C. and H.H. analyzed the data. All authors discussed the results and approved the final manuscript.
